# Exploration, expansion and definition of the atropopeptide family of ribosomally synthesized and posttranslationally modified peptides†

**DOI:** 10.1039/d4sc03469d

**Published:** 2024-09-10

**Authors:** Friederike Biermann, Bin Tan, Milena Breitenbach, Yuya Kakumu, Pakjira Nanudorn, Yoana Dimitrova, Allison S. Walker, Reiko Ueoka, Eric J. N. Helfrich

**Affiliations:** a Institute for Molecular Bio Science, Goethe University Frankfurt Max-von-Laue Strasse 9 60438 Frankfurt am Main Germany eric.helfrich@bio.uni-frankfurt.de; b LOEWE Center for Translational Biodiversity Genomics (TBG) Senckenberganlage 25 60325 Frankfurt am Main Germany; c Senckenberg Gesellschaft für Naturforschung Senckenberganlage 25 60325 Frankfurt am Main Germany; d Department of Chemistry, Vanderbilt University Stevenson Center 7330 Nashville TN 37240 USA; e Department of Biological Sciences, Vanderbilt University VU Station B, Box 35-1634 Nashville TN 37235 USA; f School of Marine Biosciences, Kitasato University 1-15-1 Kitasato, Minami-ku Sagamihara Kanagawa 252-0373 Japan

## Abstract

Ribosomally synthesized and posttranslationally modified peptides (RiPPs) constitute a diverse class of natural products. Atropopeptides are a recent addition to the class. Here we developed AtropoFinder, a genome mining algorithm to chart the biosynthetic landscape of the atropopeptides. AtropoFinder identified more than 650 atropopeptide biosynthetic gene clusters (BGCs). We pinpointed crucial motifs and residues in leader and core peptide sequences, prompting a refined definition of the atropopeptide RiPP family. Our study revealed that a substantial subset of atropopeptide BGCs harbors multiple tailoring genes, thus suggesting a broader structural diversity than previously anticipated. To verify AtropoFinder, we heterologously expressed four atropopeptide BGCs, which resulted in the identification of novel atropopeptides with varying peptide lengths, number and types of modifications. Atropopeptides serve as a proof-of-principle for the versatile genome mining approach developed in this study that can be repurposed for the identification of RiPP and other BGCs that currently evade detection.

## Abstract 2

A machine learning-based algorithm was developed to map the biosynthetic space of a peptide family characterized by intricate 3D shapes, and facilitated the characterization of complex peptides with varying length, complexity and types of modifications.

## Graphical abstract

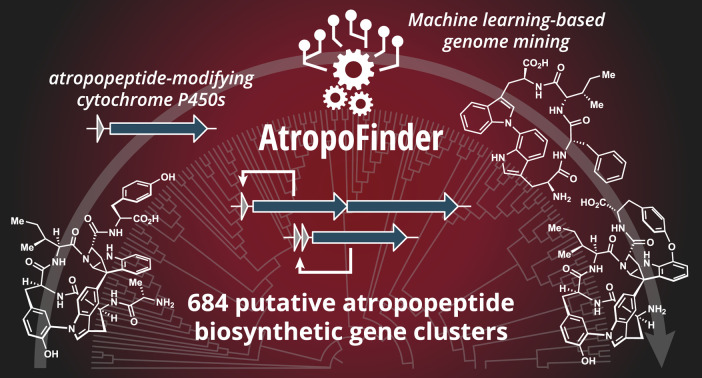

## Introduction

Peptide natural products play a crucial role in drug discovery due to their diverse structures, high potency, selectivity, and strong affinity towards drug targets, as well as favorable pharmacokinetic properties.^1^ Examples of pharmaceutically relevant peptides include vancomycin (antibacterial), cyclosporin (immunosuppressive), bacitracin (antibacterial), and thiostrepton (antibacterial).^2–5^ Historically, complex peptide natural products were believed to be solely biosynthesized by non-ribosomal peptide synthetases (NRPSs).^6,7^ NRPSs are giant multi-enzyme complexes that assemble complex peptides in an assembly line-like fashion independent of the ribosome. However, over the past two decades, numerous complex peptides have been discovered that are biosynthesized by ribosomally synthesized and posttranslationally modified peptide biosynthetic pathways (RiPPs).^8^ The array of characterized posttranslational modifications in RiPP systems is constantly increasing. RiPPs encompass a heterogenous group of natural products, which can be subdivided into more than 40 families.^8^ The corresponding biosynthetic gene clusters (BGCs) can be distinguished by the presence of characteristic tailoring genes (*e.g.*, linear polyazole-containing peptides), conserved precursor genes (*e.g.*, the Nif11-like proteusin precursors) or the intricate structure of the associated peptide (*e.g.*, lasso peptides).^9^ State-of-the-art genome mining tools frequently overlook RiPP BGCs that do not belong to the well-studied RiPP families.^10^ Consequently, RiPP-derived products are sometimes initially misannotated as NRPS products.^7,11,12^ Extensive research into RiPPs over the past two decades has expanded the RiPP biosynthetic repertoire and revealed substantial overlap in the diverse array of modifications observed in peptides from RiPP and NRPS pathways (Fig. S1†).^7,13^ As a result, the annotation of complex peptides as nonribosomal peptides (NRPs) or RiPPs can often be challenging unless peptide class-defining modifications are present in the molecules.^7^ This challenge can be showcased by tryptorubin A which was initially associated with a NRPS BGC.^11^ The proposed BGC was the sole peptide BGC detected by state-of-the-art genome mining platforms to be present in all three reported tryptorubin A producers.^11^ Tryptorubin A (1) is a hexapeptide that features an unusually complex three-dimensional structure.^14^ The extremely rigid three-dimensional shape arises from a biaryl and a carbon–nitrogen bridge between aromatic amino acid side chains and an additional carbon–nitrogen bridge between an aromatic amino acid side chain and the peptide backbone, respectively.^14^ These modifications result in two possible unusual atropisomeric configurations, only one of which has been found in nature.^14^ Unusual atropisomerism (recently also referred to as ansamerism^15^) is a type of stereoisomerism in which both isomers are theoretically interconvertible by multiple nonphysical bond torsions.^14^ Intrigued by the structural complexity of the hexapeptide, we revisited its biosynthesis. A combination of manual genome mining and heterologous expression studies unveiled that tryptorubin A is the first member of a new RiPP-family that we named atropopeptides (also referred to as atropitides^8^).^16^ The biosynthetic blueprint for the production of characterized atropopeptides resides within small BGCs containing only two genes: one encoding a precursor peptide and another a cytochrome P450 monooxygenase.^16^

**Fig. 1:**
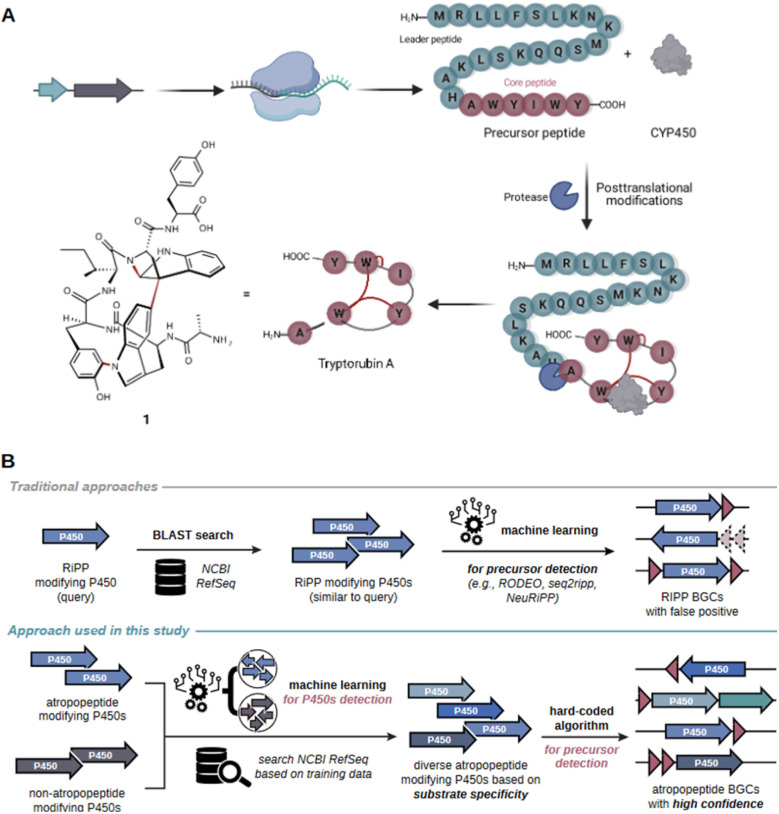
Schematic overview of the proposed model for tryptorubin A (1) biosynthesis and comparison of the genome mining concept used in AtropoFinder to state-of-the-art genome mining tools for the identification of RiPP BGCs. (A) The genes of the *trp* BGC are transcribed and the resulting mRNA ribosomally translated. Subsequently, the precursor peptide, consisting of a core peptide and a leader peptide, is posttranslationally modified by the BGC-encoded cytochrome P450 installing the characteristic atropopeptide crosslinks. Finally, an ubiquitous protease cleaves the leader peptide to release the hexapeptide tryptorubin A (1).^16^ (B) Comparison of existing machine learning approaches for RiPP discovery with the strategy developed in this study. State-of-the-art tools are frequently based on the BLAST or HMM-based search of putative RiPP-modifying tailoring enzymes.^17–20^ In a second step, machine learning algorithms are used to identify putative precursor genes in the genomic vicinity of the genes encoding the identified tailoring enzyme.^17,18,21–23^ In contrast, our approach first uses machine learning to determine atropopeptide-modifying tailoring enzymes with high precision. In a second step, a hard coded algorithm is used to identify the corresponding precursor genes with a high level of confidence.

The precursor peptide, comprising a leader and core peptide, is ribosomally synthesized. The leader peptide recruits the BGC-encoded cytochrome P450 which introduces the distinctive biaryl and carbon–nitrogen bridges in an atropospecific manner (Fig. 1A).^16^ In the final step, the modified core peptide is proteolytically released from the leader peptide. It is believed that an ubiquitous protease catalyzes the release of the mature hexapeptide, which can be further modified through the removal of the N-terminal alanine residue by another unknown peptidase that is not encoded in the BGC to yield the pentapeptide variant.^16^ A similar cytochrome P450 catalyzed biaryl-linkage was also found in biarylitides, a RiPP family described in members of the genus planomorospora.^24^

In recent genome mining efforts, the atropopeptide-modifying P450 encoded in the *trp* BGC was subjected to BLASTp analysis to identify homologs likely involved in atropopeptide maturation. The associated precursor peptide sequence was subsequently discerned through manual analysis of the genomic vicinity of the putative atropopeptide modifying P450s. While this approach has extended the atropopeptide family to encompass the cihunamides1,^8^ kitasatides^25^ and amyxirubins^16^ (also referred to as clavorubins^26^), it is apparent that this BLAST-based approach has limitations in capturing the full biosynthetic diversity of the atropopeptides. The BLASTp algorithm excels at detecting closely related homologs of a query sequence.^10^ However, to identify more distant relatives of tryptorubin A, a more flexible strategy allowing significant deviations from the original sequence is imperative. In light of these challenges, PSI-BLAST has been employed for its capability to detect more distantly related sequences through iterative searches. However, PSI-BLAST also has its limitations, as it additionally identifies a large number of P450 enzymes that are not involved in atropopeptide biosynthesis. In fact, a substantial fraction of the identified P450s are encoded in RiPP BGCs that belong to other RiPP families.^27^ The precise identification of exclusively atropopeptide BGCs might be achieved through the utilization of supervised machine learning (ML).^28^ Supervised learning entails training algorithms on a labeled dataset, which are subsequently used to classify previously unseen data by the trained algorithm.^29^

Herein we comprehensively explore the biosynthetic potential encoded in atropopeptide BGCs. We introduce AtropoFinder, a machine learning-based genome mining tool for the identification of atropopeptide BGCs. The AtropoFinder algorithm identified 683 putative atropopeptide BGCs across publicly accessible genome sequences, thus more than doubling the number of atropopeptide BGCs compared to BLAST analysis (683 putative BGCs found by AtropoFinder *vs.* 282 putative BGCs identified by Liu *et al.*^30^ Fig. S5B†). The identified atropopeptide BGCs revealed unexpected trends in core peptide composition and length, enabling us to refine the definition of atropopeptides and expand their biosynthetic and structural space. The expansion of the atropopeptide biosynthetic space is furthermore showcased by the AtropoFinder-guided identification of four new atropopeptides featuring different core peptide length, number and types of modifications. Moreover, we report the unexpected production of two atropopeptides with the same core peptide sequence but distinct non-overlapping modification patterns from a single atropopeptide BGC. Our machine learning-based approach is versatile and can be applied for the tailoring enzyme-guided discovery of BGCs associated with diverse natural product classes that currently evade unrecognized by genome mining tools.

## Results and discussion

### P450-guided identification of putative atropopeptide gene clusters (AtropoFinder)

Recent studies conducted by our research group and other laboratories describe the BLAST-based identification of atropopeptide biosynthetic pathways using the atropopeptide family-defining P450 as a query sequence.^16,25,27,30–32^ Manual analysis of the obtained results indicated that the identified core peptide sequences in the vicinity of the identified P450 genes exhibit only a moderate level of sequence diversity.^16^ This lack of sequence diversity, suggests that the BLASTp/PSI-BLAST approach might not comprehensively capture the entire biosynthetic diversity of atropopeptides. Furthermore, the workflow is time-intensive and requires manual search to locate the associated precursor peptide gene in the proximity of the P450 gene. The challenge of identifying the precursor sequence is exacerbated with precursor peptide sequences that display only limited similarity to known atropopeptide precursors (the leader peptides within the final dataset show only 4.5% identical sites and 54.3% pairwise identity). To address these limitations, we hypothesized that a machine learning-based approach to identify putative atropopeptide-modifying P450s is more suitable to comprehensively map the atropopeptide biosynthetic landscape. In addition, our approach aims to replace the laborious manual precursor peptide identification with a robust algorithm that detects the precursor and core peptides regardless of their level of homology to characterized atropopeptides. We decided to use the atropopeptide family-defining P450s as bait for the identification of atropopeptide BGCs. After the initial identification, a subsequent step, focused on identifying the ORF coding for the precursor peptide, serves to confirm the identified BGC (CoreFinder). This step also allows for the detailed identification of the primary amino acid sequences of both precursor and core peptides.

RiPP families are frequently characterized through the BLAST or HMM-based identification of putative biosynthetic gene clusters (BGCs) based on the sequence homology of family-defining tailoring enzymes within a RiPP (sub-)family. Algorithms use sequence homology either directly, as in the cases of antiSMASH 7,^19^ PRISM4,^20^ NeuRiPP,^21^ RODEO2,^17^ or indirectly like DeepBGC,^33^ GECCO,^34^ and BiGCARP,^35^ which are trained on the sequences of HHM-derived Pfam domains within known gene clusters. In a subsequent step, machine learning algorithms such as RODEO2,^17^ seq2ripp,^18^ NeuRiPP,^21^ or DeepRiPP^22^ are employed to determine putative precursor sequences. Our approach markedly diverges from this strategy. We utilize machine learning to identify atropopeptide-modifying enzymes with less emphasis on their sequence homology to characterized members of the RiPP family than previous approaches. Following this step, a hard-coded algorithm is applied to pinpoint the precursor, verify the initial machine learning step, and complete the full genome mining process within one tool (Fig. 1B).

To obtain a training data set for the ML classifier, a BLAST search was conducted using the P450 encoded in the *trp* BGC as a query sequence (WP_007820080.1). Through manual curation, we identified 51 sequences putatively encoding tryptorubin A-like precursors (ESI File S5†). After dereplication with a 95% sequence similarity cutoff, 37 sequences remained. To train the ML-classifier to differentiate atropopeptide-modifying P450s from P450s that modify other substrates, we collected a dataset from the antiSMASH database.^36^ This dataset consisted of P450s encoded in BGCs involved in the biosynthesis of products that are unrelated to atropopeptides that were dereplicated at a 95% cutoff, totaling 9065 entries. We simplified the amino acid code based on the amino acids' physico-chemical properties to prevent overfitting (Fig. S2 and S3†). Next, we trained a machine-learning Random Forest classifier on a subset of the training data, which we validated using the remainder of the training dataset. The Random Forest classifier distinguishes relevant from irrelevant features during training. It uses random subsets of training data and features (four-amino-acid motifs in P450 protein functional regions) for each decision tree. Each tree independently selects features that best differentiate ‘atropopeptide-modifying P450’ from ‘non-atropopeptide-modifying P450.’ Thus, this process identifies the specific properties of atropopeptide modifying peptides from the training data.^37^ We then applied the classifier to a dataset of 154 364 protein sequences from NCBI RefSeq that were annotated as “P450” (for a detailed workflow and metrics see ESI Detailed description of AtropoFinder, Fig. S2–S5 and Table S1†). In the initial round of P450 classification, the algorithm identified 202 putative atropopeptide-modifying P450s. However, the associated precursors, predicted by CoreFinder (see below), only showed moderate core peptide sequence variations. To address these limitations, we iterated the process, incorporating the 202 newly identified atropopeptide-modifying P450s into the initial training dataset to refine the algorithm. Using the refined classifier, we identified 440 additional putative atropopeptide-modifying P450s from the dataset of 154 364 putative P450 sequences, thus more than doubling the number of putative atropopeptide-modifying P450s in this round.

### Identification of putative atropopeptide precursor- and core peptides (CoreFinder)

More than a dozen highly sophisticated genome mining tools for the identification of putative RiPP precursor peptides have been developed.^10^ While these tools have led to the identification of countless putative precursor sequences and the targeted discovery of numerous RiPPs, none of the existing tools are capable of identifying atropopeptide precursors and BGCs, respectively (ESI Comparison of AtropoFinder to existing bioinformatic tools†). The manual search process for atropopeptide precursor genes is not only laborious but may also miss out on sequences that deviate significantly from known atropopeptide precursors. To address this problem, we introduced “CoreFinder” (detailed description can be found in ESI Detailed description of CoreFinder†). The command-line tool streamlines the screening for open reading frames (ORFs) in the genomic vicinity of a specified protein-encoding gene (here specifically the gene encoding the characteristic atropopeptide-modifying P450) within all GenBank nucleotide records in which the query protein is annotated. We screened the genetic neighborhood of all putative atropopeptide-modifying P450 genes from NCBI records for atropopeptide-like open reading frames. The identification criteria prioritize precursor ORFs that range between 10 to 40 amino acids in length, consisting of a leader and a core peptide, which contains two consecutive aromatic amino acids and is followed by a stop codon. The latter criterion stemmed from the observation that the atropopeptides we had previously characterized all featured at least one pair of consecutive aromatic acids that are crucial for anchoring the characteristic atropopeptide modifications. CoreFinder detected 803 potential precursor peptide-encoding genes. Out of these, 475 genes from 533 genomes exhibited overall sequence homology to the tryptorubin A precursor.

### Analysis of atropopeptide precursor sequences and recalculation of putative atropopeptide precursor sequences

**Fig. 2:**
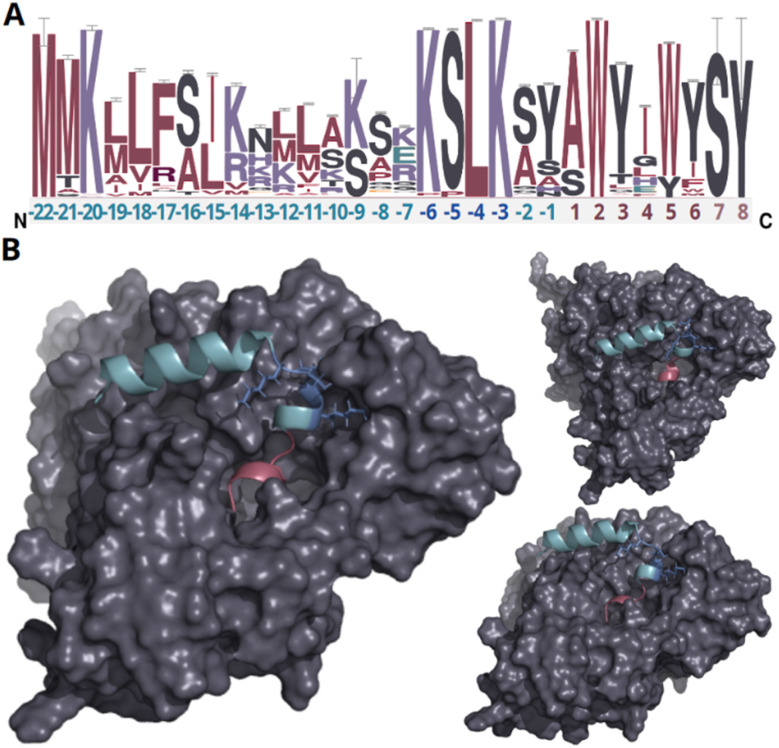
Analysis of putative precursor peptides and modeling of protein interactions of the precursor peptide and P450 encoded in the *trp* BGC. (A) Sequence logo of putative precursor peptide sequences. The amino acids of the leader peptide, KSLK motif and core peptide are depicted in green, blue, and red, respectively. Residues only present in very few peptides are depicted in light red (residue 7–8). The height of each letter in the sequence logo corresponds to the frequency of the respective amino acid at that position, with taller letters indicating higher frequency and conservation. The error bars indicate an approximate Bayesian 95% confidence interval. (B) AlphaFold2 multimer protein model of WP_007820080.1 and the tryptorubin A precursor peptide from different angles (a video showcasing intricate details of the protein model can be found in ESI file S4,† metrics of the modeling can be found in Fig. S7†). The leader peptide, KSLK motif, core peptide, and cytochrome P450 are depicted in green, blue, red, and gray, respectively. The leader peptide forms an α-helix that bends at the KSLK motif. The KSLK motif acts as an anchor of the precursor to the P450 that allows the core peptide to move freely within the active site.

**Fig. 3:**
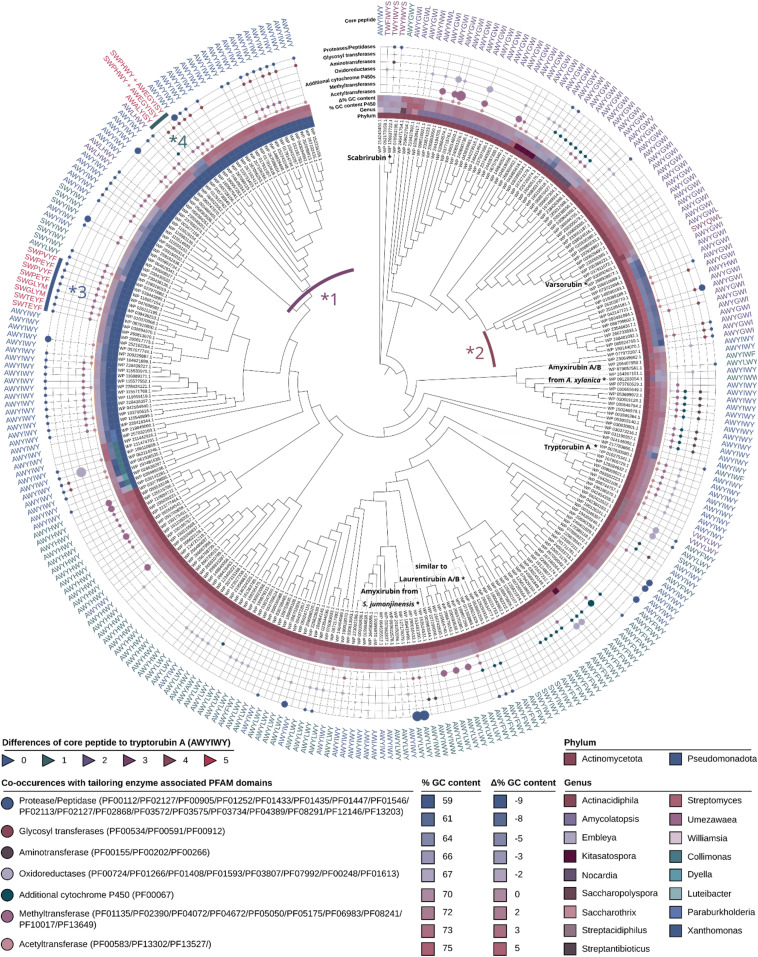
Phylogenetic tree of characteristic atropopeptide-modifying P450s encoded in putative atropopeptide BGCs, putative core peptide sequences as determined by CoreFinder and tailoring enzymes encoded in the associated BGCs. Depicted is the genus and phylum of the producer, the co-occurrence of the atropopeptide-defining P450 with tailoring enzyme-associated PFAM domains, the associated putative core peptide and its similarity to the tryptorubin A sequence. Additionally, the GC content of the P450 (absolute and relative to the GC content of the producer) is shown. The clade marked with *1 contains clusters with significantly higher GC content than the producer genome whereas the clade marked with *2 contains clusters with a significantly lower GC content than the genome of the producer, indicating relatively recent horizontal gene transfer events between the two producer phyla. The clade marked with *3 is associated with core peptides not containing two tryptophans and the clade marked with *4 is associated with two precursor peptides for each BGC.

In our endeavor to characterize conserved motifs within atropopeptide precursors, we aligned all identified putative precursor peptide sequences (ESI file S1†). We used the MEME suite^38^ and identified a conserved KSLK motif (or closely related motifs like ‘RSLK’, ‘ESLK’, ‘KSRK’, ‘KPLK’, or ‘PSLK’) strategically situated between residues 17–20 of the putative precursors (Fig. 2 and S6†). We thus hypothesized that the KSLK motif might be necessary for the recognition of the precursor by the P450. To verify our hypothesis we used AlphaFold2 multimer^39^ to investigate the predicted interaction between the precursor and corresponding P450 associated with tryptorubin A (Fig. 2). With a iptm + ptm score of 0.8395, the model seems to be a good representation of the protein interaction (for details on the pLDDT score for each residue see Fig. S7†). Our analyses underscored the putatively critical role of the KSLK motif for threading the precursor into the P450's substrate binding tunnel. The KSLK motif occupies a strategic junction, marking the transition from the N-terminal α-helix of the leader peptide—which is predicted to bind to the P450 enzyme's surface—to a second, shorter α-helix which transitions into the seemingly flexible core peptide. Based on the AlphaFold model, the KSLK motif can be regarded as a biochemical “anchor”, threading the precursor peptide into the substrate binding tunnel. This stabilization might allow the core peptide to “swirl” around within the enzyme's active site. Consequently, this flexibility likely renders the formation of different bond types at different locations within the core peptide possible. Guided by the lessons learned from the combination of sequence analysis and modeling studies, we refined the classification algorithm of CoreFinder to mandate the inclusion of one of the following motifs within the precursor: ‘KSLK’, ‘RSLK’, ‘ESLK’, ‘KSRK’, ‘KPLK’, or ‘PSLK’. The recomputation unveiled 685 putative precursor peptides dispersed across 683 nucleotide sequences, in the vicinity of 328 of the 440 putative atropopeptide p450s. Among these, 684 exhibited archetypal features of atropopeptide precursors. Only a single sequence deviates from the common precursor architecture. Although it possesses the “KSLK” motif, it significantly diverges from canonical leader peptide sequences and appeared to adopt this motif at an aberrant locus. Such deviation might represent a fusion of an atropopeptide BGC with another RiPP family. Two putative atropopeptide BGCs seemingly harbor two instead of one putative precursor gene (Fig. 3). A BiG-SCAPE^40^ analysis comparing all obtained gene clusters with BGCs from MIBiG^41^ shows that atropopeptide BGCs cluster separately from all BGCs deposited to MIBiG^41^ (ESI file S3 and Fig. S8†). This finding indicates atropopeptide BGCs not being related to any BGCs deposited to MIBiG. In a sequence similarity network of all identified putative precursor peptides (ESI file S2 and Fig. S9†), the precursor sequences group based on their core peptide sequence and phylogenetic origin. Surprisingly, the sequences associated with the tryptorubin A core peptide group into multiple clusters, suggesting that they might have evolved towards this sequence multiple times. A similar evolutionary pattern is observed in the phylogenesis of atropopeptide-modifying cytochrome P450s (Fig. 3).

### Analysis of atropopeptide core sequences

The analysis of all identified putative core peptides revealed that the majority are likely hexapeptides. Nonetheless, there are deviations from this norm. 14 are discerned as octapeptides, while three are classified as heptapeptides. Intriguingly, the octapeptides appear to incorporate additional amino acids interspersed amidst the known modified amino acids (W2, Y3, W6, Y7). In contrast, the heptapeptides exhibit an appended serine at their C-terminus (Fig. 3). Amidst the core peptide sequences (Fig. 2A), the only conserved amino acid residue is W2. We hypothesize that W2 plays a key role in one of two ways: it is either vital for forming the atropopeptide's 3D shape, especially considering all characterized atropopeptides have at least one if not multiple crosslinks anchored to this tryptophan residue. Alternatively, W2 might be crucial for binding to the P450, although our modeling studies (Fig. 2B) do not corroborate this hypothesis. A striking observation is that not all putative core peptides encompass two successive aromatic amino acids, potentially implying varied modification motifs. Moreover, in comparison to a recent report,^31^ not all core peptides exhibit a pair of tryptophan residues. Several core peptides originating from *Xanthomonas* species display an xWxxYx pattern, deviating from the conventional xWxxWx pattern reported before.^31^ This observation suggests that the recently coined bitryptides (xWxxW) refer to a subfamily of atropopeptides. Moreover, our *in silico* studies indicate that atropopeptides can be more adequately classified by the presence of conserved motifs and residue in the precursor peptide than by their complex 3-dimensional shape.

### Phylogenetic distribution of atropopeptides

The phylogenetic distribution of putative atropopeptide BGCs revealed their abundance mainly in *Streptomyces*, *Xanthomonas*, and related genera in the phyla actinomycetota and pseudomonadota (Fig. 3 and S10†). This abundance is surprising because these phyla are not closely related within the bacterial branch of life. To delve deeper into the evolutionary history of P450s encoded in putative atropopeptide BGCs, we constructed a maximum likelihood phylogenetic tree utilizing all putative atropopeptide-modifying P450 amino acid sequences (Fig. 3). Notably, the sequences group based on the phylogeny of their producer organisms and the associated putative core peptide sequences.

Upon contrasting the GC content of the P450s (serving as a proxy for the GC content of the BGC) with the mean GC content of each species' genome, we found a close correlation between the two for most BGCs. The observed pattern suggests that these BGCs have an ancient evolutionary origin and have adapted to the GC content of the producer's genomes. However, two distinct clades of P450s presented significant disparities in their GC content relative to their producer's genome GC content. Within the clade designated as *1, the P450's GC content exceeds that of the associated Pseudomonadota genomes, mirroring instead the GC profile characteristic for Actinomycetota. In contrast, the clade labeled as *2 reveals a GC content of the P450 that is markedly diminished relative to its corresponding Actinomycetota producer strains, but aligns more closely with GC contents characteristic for Pseudomonadota. Such observations might allude to recent horizontal gene transfer events between the two phyla.

### Analysis of the genomic vicinity of minimal atropopeptide BGCs

For an in-depth analysis of the tailoring enzymes encoded in the putative atropopeptide BGCs, we employed RODEO's functionality to track concomitant Pfam domains encoded by a specified gene.^17^ Our findings underscore a frequent association between proteases and atropopeptide P450s within the genus of *Xanthomonas*. This is particularly noteworthy, as previously characterized atropopeptides do not harbor proteases encoded in their associated BGC. This association potentially facilitates the cleavage in instances where an external protease is absent for leader peptide excision or the proteases/peptidases are responsible for a secondary cleavage of the core peptide, reminiscent of the model for tryptorubin B biosynthesis. Other tailoring enzymes that are frequently encoded in atropopeptide BGCs are acetyltransferases, methyltransferases, oxidoreductases, additional P450s, and glycosyltransferases. It is tempting to speculate that the biosynthetic space of atropopeptides includes molecules that are more extensively modified by the identified tailoring enzymes than the previously characterized atropopeptides.

### AtropoFinder-based expansion of the atropopeptide chemical space

To validate the AtropoFinder algorithm and expand the biosynthetic and chemical space of characterized atropopeptides, we selected four putative atropopeptide BGCs for characterization through heterologous expression in *Streptomyces albus* J1074.

**Fig. 4:**
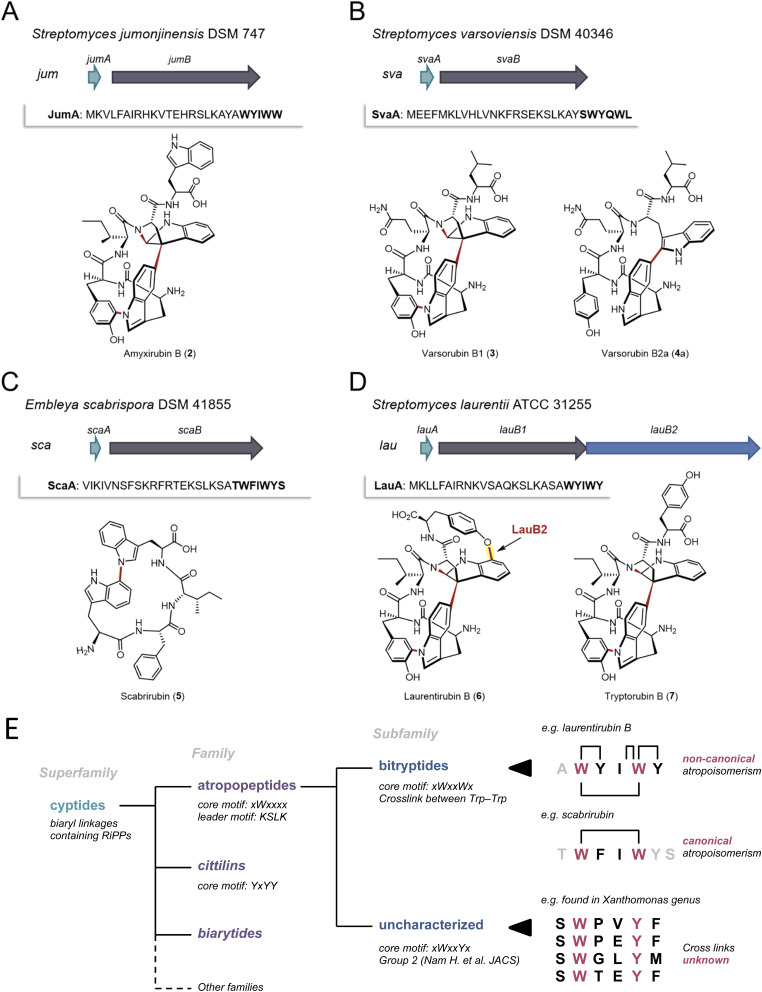
Characterization of four putative atropopeptide BGCs and overview over the landscape of RiPPs that are characterized by P450-mediated crosslinks between amino acid side chains. The precursor genes and genes encoding the atropopeptide family-defining P450s are depicted in green and gray, respectively. The additional P450 is depicted in blue. The precursor peptide sequences are shown and the putative core peptides are highlighted in bold. (A) *jum* BGC and structure of purified amyxirubin B (2) associated with the *jum BGC*. (B) *sva* BGC and the structure of varsorubin B1 (3), varsorubin B2a and varsorubin B2b (4a) obtained by heterologous expression of *svaA* and the P450 encoding gene *svaB* in front of which a RBS was inserted. (C) *sca* BGC and structure of purified scabrirubin (5) obtained by heterologous expression of the *sca BGC*. (D) *lau* BGC and structure of purified laurentirubin B (6) obtained from the heterologous expression of the full *lau* BGC and tryptorubin B (7), the product obtained from coexpressing *lauA* and *lauB1*. For a more detailed overview of the *lau* BGC, see Fig. S55.† (E) Overview over the landscape of RiPPs that are characterized by P450-mediated crosslinks between amino acid side chains and comparison of the characteristics of the individual RiPP families and subfamilies with an emphasis on the atropopeptide family.

We first selected the atropopeptide BGC from *Streptomyces jumonjinensis* DSM 747 (Genbank: GCF_009600885.1, NZ_VCLA01000160.1 22.964:24.216, further referred to as *jum*) which was predicted to harbor the same core peptide sequence as the one reported for amyxirubin A and B produced by *Amycolatopsis xylanica*.^16^ Since both organisms belong to the same class (*Actinomycetes*) but a different order, the identity of the P450s encoded in the *jum* and amyxirubin BGCs was only 59% and the P450 phylogeny suggested a distant relationship of the P450s encoded in the *jum* and amyxirubin BGCs (Fig. 3), we speculated that the resulting atropopeptide might show a different bridging pattern when compared to amyxirubin. Heterologous expression of the *jum* BGC led to the isolation of 1.37 mg of the associated atropopeptide (2) (Fig. 4A and S11†). The comparison of ^1^H NMR chemical shifts and high-resolution electrospray ionization mass spectrometry (HR-ESI-MS) data of 2 and an authentic standard of amyxirubin B (Fig. S12 and S13†), however, revealed that the structure of 2 is identical to amyxirubin B, suggesting that the phylogenetic placement of the atropopeptide-modifying P450 might not be a good indicator for the structural novelty of the atropopeptides.

We next set out to characterize a putative atropopeptide BGC from *Streptomyces varsoviensis* DSM 40346 (Genbank: GCF_000718635.1, NZ_JOBF01000044.1 9.365:10.577, further referred to as *sva*). The predicted core peptide sequence of the putative precursor peptide is SWYQWL (Fig. 4B). The *sva* BGC was heterologously expressed. Extraction of HP20 resin supplemented to the culture supernatant and analysis of the crude extract by high-resolution electrospray ionization mass spectrometry (HR-ESI MS) revealed the presence of two compounds (3 and 4) detected at *m*/*z* 791.3540 [M + H]^+^ (calcd for *m*/*z* 791.3511, C_42_H_47_N_8_O_8_^+^, Δ 3.67 ppm) and *m*/*z* 793.3655 [M + H]^+^ (calcd for *m*/*z* 793.3668, C_42_H_49_N_8_O_8_^+^, Δ 1.64 ppm) (Fig. S14 and S16†) from the culture. The calculated formulae of 3 and 4 are in good agreement with molecular formulae of the predicted pentapeptide variant of the core peptide (WYQWL) that had lost four and two protons, respectively. The proton loss indicated at least one or two modifications, respectively. To increase the yield for subsequent purification, we inserted a ribosomal binding site in front of the gene encoding the P450, which resulted in a 100-fold increase in yield of the putative pentapeptides (Fig. S15†). One of the associated pentapeptides (3), named varsorubin B1 (Fig. 4B), was purified from a 16 L culture using a combination of open column chromatography and semi-preparative HPLC (yield 1.6 mg). Analysis of the ^1^H and ^13^C NMR spectra, with the aid of 2D NMR correlations (Fig. S17–S22 and Table S2†), confirmed the amino acid sequence WYQWL. Compound 3 is a pyrroloindoline-containing bicyclic macrolactam that features a C–C bond bridging two Trp residues, along with two C–N bonds between Trp-1 and Tyr-1, and between Trp-2 and the amide nitrogen. Moreover, NOESY correlations of H-11(Trp-2)/H-38(Trp-1) and H-9(Trp-2)/H-40,41(Trp-1) suggest that 3 features the “bridge-above” configuration of the bicyclic macrolactam ring as previously reported for all complex atropopeptides (Fig. S23†). Based on the recently proposed terminology for the conformational isomerism of bicyclic peptides,^15^ we assigned the configuration of the bridge-above macrolactam for 3 as *P*_ansa_.

Careful analysis of HPLC-HR-ESI-MS data revealed that the second peak contained 4 and an additional compound with the same molecular formula as 4 (Fig. S24 and S25†). Further chromatographic separation resulted in the isolation of 1.81 mg of 4a, which we named varsorubin B2a (Fig. 4B), as well as an additional isomer varsorubin B2b (4b). The structure of the isomer 4b was not unambiguously elucidated by NMR analysis due to its insufficient yield. Meanwhile, 1D and 2D NMR spectra of 4a indicated that the molecule no longer possesses the complex three-dimensional shape that is characteristic for 3 (Fig. S26–S33 and Table S3†). Instead, only a single modification (C–C bond between C-39 of Trp-1 and C-11 of Trp-2) was present in 4a. Interestingly, the position of this C–C bond at Trp-2 differs from the C–C bond in varsorubin B1 which is located at C-10 of Trp-2. 3 and 4a share the same amino acid sequence but are characterized by an unprecedented non-overlapping bridging pattern installed by the same P450 (Fig. 4B). Moreover, the two characterized products show a different degree of structural complexity.

Subsequently, we selected the putative atropopeptide BGC from *Embleya scabrispora* DSM 41855, (Genbank: GCF_000372745.1, NZ_KB889561.1, further referred to as *sca*) for characterization. The precursor ScaA harbors the putative heptapeptide core peptide sequence TWFIWYS (Fig. 4C). Culture extracts of *S. albus* harboring the *sca* BGC were screened for the presence of modified heptapeptides associated with the BGC. To our surprise, however, comparative HPLC-HR-ESI-MS analyses of extracts from *S. albus* cultures harboring the *sca* plasmid with an empty plasmid control revealed the presence of a new peak with an *m*/*z* 649.3132 [M + H]^+^ (calcd for *m*/*z* 649.3133, C_37_H_41_N_6_O_5_^+^, Δ 0.15 ppm) (Fig. S34 and S35†). The observed mass was significantly smaller than the expected precursor ion of the predicted heptapeptide. Nevertheless, we extracted and purified 3.5 mg of the putative atropopeptide (5) from a 10 L culture. Analysis of ^1^H and ^13^C NMR spectra, with the aid of HSQC, HMBC, and NOESY correlations (Fig. S36–S42 and Table S4†), revealed the presence of a tetrapeptide with the sequence WFIW featuring a C–N bond between C-34 of Trp-1 and N-5 of Trp-2 (Fig. S43†) that is in agreement with the N- and C-terminally truncated core peptide sequence. We named the cyclic tetrapeptide scabrirubin (5). The characterization of scabrirubin with the sequence WFIW from the putative heptapeptide core TWFIWYS suggests that peptidases in the heterologous host cleave both the N-terminal and the C-terminal amino acids of the core peptide, indicating that the C-terminus might serve as a follower peptide. While the removal of N-terminal amino acids is a common event during atropopeptide maturation, the removal of C-terminal residues is rare.

We next set out to characterize an atropopeptide BGC harboring an additional gene encoding another P450 to investigate possible additional tailoring reactions. AtropoFinder identified the putative BGC in *Streptomyces* sp. MMG1121, which was not available in any strain collection. We therefore conducted an NCBI BLAST^42^ homology search on the P450 to find a similar BGC in a commercially available strain. To our surprise, we identified a BGC from *Streptomyces laurentii* ATCC 31255 (Genbank: GCA_002355495.1, AP017424.1 5.439.938:5.442.374, further referred to as *lau* BGC) that harbors the characteristic atropopeptide-modifying P450 alongside three additional annotated P450 gene fragments that are significantly shorter than the first P450 encoded in the *lau* BGC (Fig. S44†). This BGC was not originally identified by AtropoFinder because we ran AtropoFinder solely on RefSeq P450s which does not include the *S. laurentii* P450. We initially hypothesized that the three P450 fragments might act together to form one functional P450. Alternatively, we hypothesized that the three P450 fragments are a sequencing artifact and that the three P450 gene fragments indeed encode one contiguous P450 such as in *Streptomyces* sp. MMG1121. To verify the latter hypothesis, we subjected the *lau* BGC to resequencing. Analysis of the sequencing data revealed that the three P450 gene fragments are indeed a sequencing artifact. As a consequence, the *lau* BGC is only predicted to harbor two genes encoding P450s, the atropopeptide-family defining P450 and a second P450 with low identity to TryB (28%) (Fig. 4D). To characterize the *lau* BGC, we cloned and heterologously expressed the BGC in *S. albus.*

HPLC-HR-ESIMS analysis of the crude extract obtained from the *S. albus* culture harboring the *lau* BGC revealed the presence of two candidate atropopeptides detected at *m*/*z* 895.3760 [M + H]^+^ (calc. sum formula C_49_H_51_N_8_O_9_^+^, Δ 1.6 ppm) and 824.3401 [M + H]^+^ (calc. sum formula C_46_H_46_N_7_O_8_^+^, Δ 0.1 ppm) to be associated with the *lau* BGC (Fig. S45 and S46†). The mass difference between the predicted core peptide sequence and the detected compounds are indicative of the candidate hexa- and pentapeptides with a loss of six protons which suggests the presence of at least three modifications. To pinpoint the localization of the modifications, the main product, 1.6 mg of a pentapeptide (laurentirubin B (6)) was isolated from a six liter culture. Based on the ^1^H NMR spectrum and 2D NMR correlations (Fig. S47–S55 and Table S5†), the structure of 6 was determined. 6 is characterized by a highly strained tricyclic macrolactam ring system that is constructed by two C–N bonds (Trp-1–Tyr-1 and Trp-2–peptide backbone), a C–C bond (Trp-1–Trp-2), and a C–O bond (Trp-2–Tyr-2) (Fig. 4). The localization of the unprecedented aryl ether linkage between C-17 of Trp-2 and C-7 of Tyr-2 is supported by density functional theory (DFT) calculations (Table S6†). Moreover, the atropisomeric configuration of *P*_ansa_ for 6 was determined by NOE correlations (Fig. S55†) and ^13^C chemical shift calculations by the DFT method (Table S6†).

We hypothesized that the three common modifications are installed by the characteristic P450 homolog that is conserved in all atropopeptide BGCs and that the aryl ether bridge is formed *via* the second P450. To verify our hypothesis, we coexpressed both the precursor LauA with the first P450 LauB1 and LauA with the second P450 LauB2 in *S. albus* and analyzed the extracts for biosynthetic intermediates. The results showed that coexpressing *lauA* and *lauB2* resulted in the complete abolishment of atropopeptide production. Coexpression of *lauA* and *lauB1*, on the other hand, resulted in the accumulation of tryptorubin B (7) (Fig. S56†), a biosynthetic intermediate with three modifications, indicating that the second P450 is responsible for introducing the fourth modification. Laurentirubin B is one of the most complex atropopeptides reported to date. With two exceptions,^30^ the *lau* BGC is the only characterized BGC that encodes an additional P450 that modifies the peptide beyond the characterized atropopeptide-specific modifications. Our gene coexpression studies furthermore indicate that the introduction of the ether bridge takes place after the formation of the archetypical atropopeptide modifications.

The antimicrobial activity of compounds 2, 3, 4a and 5 were investigated against all ESKAPE pathogens, *Arthrobacter pascens* and *Candida albicans*. Compounds 2, 3 and 4a did not show antibacterial or antifungal activity. Compound 5 displayed weak growth inhibition activity against *A. pascens* and 3 promoted the growth of *P. aeruginosa* and *A. pascens* (Fig. S57†), indicating that 3 may function as a signaling metabolite across phylum borders. This observation provides possible clues to further study the ecological role of the atropopeptides and to identify the molecular target of 3.

## Conclusion

Atropopeptides are one of the recently described RiPP families that feature characteristic P450-catalyzed post-translational modifications that result in crosslinking of amino acid side chains (*i.e.* biaryl, aryl-nitrogen and aryl-ether linkages).^8,16,25^ Other families characterized by P450-mediated posttranslational modifications include the biarylitides, that feature either a P450-catalyzed biaryl or a carbon–nitrogen bridge between histidine and tyrosine residues,^24^ or the cittilins that harbor both a biaryl and an aryl-ether bridge between tyrosine side chains. In contrast, atropopeptides feature biaryl, carbon–nitrogen, and aryl-ether bridges, rendering them the most intricate RiPP family modified by P450s.^14,16^ Recently, a RiPP superfamily, named cyptides, that includes all RiPP families that feature biaryl linkages, has been proposed.^27^ Within this superfamily, the atropopeptides (also referred to as group 1/2 cyptides and atropitides) are the largest family with over 270 previously identified members.^27^ Our efforts to systematically map the biosynthetic space of the atropopeptides resulted in the significant expansion of the number of predicted BGCs associated with the atropopeptide family over previous studies that employed BLAST-based approaches, which are inherently biased towards limited sequence diversity. Moreover, our approach that is based on the unique properties of atropopeptides exclusively detects atropopeptides BGCs and thus outperforms BLAST-based approaches that have been shown to identify RiPP BGCs beyond the atropopeptide family.

Our machine learning-based approach that uses the cytochrome P450 enzymes as a bait for the detection of atropopeptide BGCs, resulted in the identification of 684 putative atropopeptide BGCs. Insights from the comparison of all putative atropopeptide leader peptide sequences revealed a conserved KSLK motif in the leader that is characteristic for all atropopeptide leaders and that can be used to differentiate atropopeptides from other RiPP families that feature biaryl-linkages. Using AlphaFold2 multimer^39^ modeling, we were able to propose a putative role of the conserved motif in interacting with the P450, thus offering insights into its putative functional relevance as an anchor for the core peptide within the active site of the cytochrome P450. Moreover, all identified atropopeptide cores are characterized by the presence of a conserved Trp residue at the second position of the core peptide as the only conserved residue in the core. As a result, the recently proposed bitryptides^31^ that feature a xWxxWx core and the KSLK leader motif are a large subfamily of the atropopeptides (Fig. 4E). In contrast, atropopeptides are defined by the KSLK leader motif in conjunction with the xWxxxx core motif without a conserved W at position 5 in the precursor and core peptide, respectively. This observation parallels recent insights into biarylitide biosynthesis that likewise resulted in the expansion of the biarylitide biosynthetic space.^27^

Our study and recent reports from other labs^25,31^ suggest that not all atropopeptides are characterized by the complex 3-dimensional shape reported for tryptorubin A which results in two possible non-canonical atropisomeric configurations. While most of the characterized atropopeptides (amyxirubin B, varsorubin B1 and laurentirubin B in this study) feature this unusual type of stereoisomerism, the family members that do not (scabrirubin and varsorubin B2a in this study), at least, require canonical atropisomeric assignments.^31^ As a result, we propose to keep the name atropopeptides for the RiPP family. As the complex 3-dimensional shape is not a common characteristic of all atropopeptides, we propose to redefine the atropopeptide family based on their conserved biosynthetic features that include the presence of an atropopeptide class-defining P450, the conserved KSLK leader motif and a W residue at position two of the core peptide (Fig. 4E).

To validate the AtropoFinder algorithm, we characterized four atropopeptide BGCs and elucidated the structures of their associated compounds. Scabrirubin (5) features a single carbon–nitrogen bond between two tryptophan residues of the tetrapeptide core; the *sva* BGC produces varsorubins (3,4a and 4b) varying in complexity with three and one modifications, respectively, and laurentirubin B (6), one of the most complex atropopeptide reported to date, harbors a total of four modifications including an unprecedented ether bond between Trp-2 and Tyr-2. The presence of three different products with the same core peptide sequence and length (varsorubins) is remarkable as previous reports indicate the full conversion of the core peptide sequence into a single product. Moreover, the *lau* BGC one of the few characterized atropopeptide BGCs that does not harbor the minimal BGC architecture made up of precursor and P450 genes. The formation of the ether bond after the atropospecific formation of the bridge-above configuration of a tryptorubin-like intermediate by the second P450 encoded in the BGC impacts the 3-dimensional shape of the molecule. The highly rigid conformation of 6 was confirmed by the presence of asymmetric ^1^H and ^13^C NMR signals for the aromatic ring of Tyr-2 residue, along with DFT calculations (Fig. S58†).

The three conserved bridges that are present in most characterized atropopeptides result in the formation of a highly strained bimacrocyclic ring that shows non-canonical atropisomerism. In accordance with the *P*_ansa_/*M*_ansa_ nomenclature for the stereochemical description of conformationally diastereomeric bismacrocyclic peptides which was recently defined by Süssmuth and co-workers,^15^ we determined the configuration of the bicyclic macrolactam rings for all characterized atropopeptides as *P*_ansa_. This nomenclature is applicable for the configurational description of atropopeptides in case they feature a bicyclic ring system. The only amino acid not involved in the formation of the bicyclic ring system in atropopeptides is the relatively flexible sixth amino acid of the core (*e.g.*, Tyr6) which is not locked into place by the three characteristic modifications. In the case of laurentirubin B, the fourth modification restrains the otherwise conformationally flexible Tyr6 in the form of a cyclophane lactam, making the 3D structure more rigid.

The number of modifications installed into atropopeptides reported here ranges from one to four and the number of bond types from one (scabrirubin), to two (*e.g.*, varsorubins) and three (laurentirubin B) that are installed by a single multi-functional P450 or alternatively four through the joint action of two P450s in the case of laurentirubin B.

In conclusion, our research expands the atropopeptide family by more than 50%, provides a comprehensive understanding of atropopeptide biosynthesis and timing of maturation. The systematic investigation of atropopeptide biosynthetic space lays the foundation for future investigations into this peptide family and showcases the potential of machine learning-based genome mining algorithms for the identification of non-canonical biosynthetic pathways that elude unrecognized by existing genome mining tools. The characterization of the atropopeptide RiPP family serves as a proof-of-concept for the versatile genome mining concept developed in this study. The machine learning-based identification of unique features in tailoring enzymes complements current genome mining strategies to chart biosynthetic dark matter. We believe that our approach can be adapted by retraining the classifier on different training data sets to chart the biosynthetic space of other RiPP families, to identify overlooked RiPP BGCs beyond family borders and other currently overlooked natural product BGCs.^28,43^

## Experimental

### Materials

All chemicals, reagents and solvents were purchased from Sigma-Aldrich or Carl Roth (Germany), unless stated otherwise. Antibiotics and medium components were purchased from Carl Roth (Germany). Oligonucleotide primers were synthesized by Microsynth AG (Germany). All restriction enzymes, Q5 High Fidelity DNA polymerase, HiFi DNA Assembly master mix, T4 DNA ligase, deoxynucleotides (dNTPs) and DNA Ladder were purchased from New England Biolabs (UK). Monarch™ DNA gel extraction kit, Monarch™ Genomic DNA Purification Kit and Monarch™ Plasmid Miniprep kit from New England Biolabs (UK) were used to purify DNA.

### Methods

#### Generation of the training data set

For the development of the machine learning (ML) classifier, a negative dataset was assembled from the antiSMASH database,^36^ comprising 14 665 proteins annotated as ‘cytochrome p450.’ Concurrently, a positive dataset was meticulously curated by deploying the tryptorubin A cytochrome P450 WP_007820080.1 as a query for Blastp analysis against the NCBI non-redundant protein sequences (nr) database. From this analysis, 51 putative biosynthetic gene cluster (BGC) sequences were identified through a manual search for precursors situated in the genetic neighborhood of each Blast hit. To dereplicate both datasets, cdhit V4.8.1 ^44^ was employed. Parameters were configured to cluster sequences with a minimum of 95% sequence similarity and to utilize a word size of 5. As a result of this dereplication process, the negative and positive datasets were refined to 9065 and 37 sequences, respectively.

#### Assembly of classification dataset

For the classification, a dataset comprising 154 364 protein sequences was derived from the NCBI Identical Protein Groups database (25.01.2023).^45^ These sequences, which encompassed the term “p450” in their descriptions and which had lengths ranging from 300 to 450 amino acids, were procured using the following query parameters: p450[All Fields] AND (refseq[filter] AND prokaryotes[filter] AND (“300”[SLEN]:“450”[SLEN])).^45^ The method employed to assemble the dataset mirrored that of the training dataset.

#### Data preprocessing

Sequences were aligned using CLUSTALW (version 1.2.3 ^46^) with the default settings (clustalo-1.2.3-Ubuntu-x86_64 -i input.fasta -o clustal.aln -v --outfmt=clustal --output-order=tree-order --auto -t Protein) with a reference cytochrome P450 with known annotation of functional regions from *Mycobacterium tuberculosis* H2102 (GenBank: KBE51585.1) in a multiple sequence alignment. The sequences were fragmented at positions 92, 192, 275, and 395 relative to the reference sequence. The resulting fragments represent the different functional regions of the cytochrome obtained from the annotations in the Cytochrome P450 Engineering Database record 10 800 (N-terminus, substrate binding region 1, substrate binding region 2, core region and C-terminus).^47^

To prevent overfitting, the sequences were translated into a simplified amino acid code (Fig. S3†).

#### Training of the classifier and hyperparameter optimization

The machine learning model was implemented using scikit-learn version 1.0.2 ^48^ for python. The dataset was balanced using the Random Over Sampler from the Python package Imbalanced-learn 0.9.0.^49^ A 60 : 40 split for the training dataset and internal validation set was used. The number of overlapping *k*-mers of motifs with the length of four that occurred in at least half of the 37 putative atropopeptide sequences in each segment were used as features. Different classifiers were compared and the Random Forest classifier was chosen because of its high f1 score for atropopeptide P450s (0.97). Hyperparameter tuning was performed to optimize the maximum amount of samples per leaf and the maximum tree depth assessed on the obtained maximum balanced accuracy (balanced accuracy = (recall + specificity)/2) (optimal parameters: 1 and 10, respectively). Every hit with a score >0.15 was considered “positive”. After the first run on the classification dataset, 202 putative atropopeptide cytochrome p450s were curated from the results using CoreFinder and sequence alignments. These additional putative atropopeptide cytochrome P450s were chosen to supplement the training dataset, leading to a size of 113 for the positive training data set after deduplication with cd-hit V4.8.1 ^44^ (cutoff 0.95, word size = 5). The classifiers underwent training on the newly acquired dataset, according to the previous methodology (f1 score = 0.97). Parameter optimization yielded optimal values of a singular leaf (maximum leaves = 1) and a tree depth constrained to 14 (maximum tree depth = 14).

#### Corefinder

Potential genes encoding precursor and core peptides were investigated in the 3 kb genetic neighborhood, both upstream and downstream of the putative atropopeptide cytochrome P450 genes in all relevant nucleotide records. Open reading frames (ORFs) ranging between 10 to 40 amino acids that contained either two consecutive aromatic amino acids within their six amino acid core peptide (first round) or one of the motifs ‘KSLK’, ‘RSLK’, ‘ESLK’, ‘KSRK’, ‘KPLK’, or ‘PSLK’ in the precursor peptide (second round) were selected. ORFs that overlapped with annotated coding sequences (CDS), with the exception of those labeled as “tryptorubin family RiPP precursor CDS” were ignored. Overlapping cores were sorted out to remove duplicates with slightly longer putative leader peptide encoding genes. It is noteworthy, that the exact start and thus length of the precursor peptides cannot be determined by Corefinder if multiple putative start codons are present. Corefinder uses fasta files containing protein identifiers and outputs a genbank file containing the region 3 kb upstream and downstream with annotated precursor peptides.

#### Comparison with other tools

To compare AtropoFinder to state-of-the-art genome mining tools, all Genbank files of putative atropopeptide regions from the AtropoFinder output were converted to fasta files and merged to create a record that could then be analyzed by the respective tool. We used GECCO version 0.9.6,^34^ DeepBGC version 0.1.23,^33^ the antiSMASH version 7 webserver^19^ (strictness: loose), the PRISM 4 webserver^20^ and RODEO 2 ^17^ all on default settings. The plot in Fig. S5† was created using BGCViz.^50^

#### Sequence logos

The sequence logos were created using WebLogo 3 ^51^ from sequence alignments of core peptides and precursor peptides using CLUSTALW version 1.2.3 ^46^ with the default settings.

#### Sequence similarity networks

A sequence similarity network was generated utilizing the EFI-EST^52^ platform from all putative atropopeptide precursors that contain the “KSLK” or a related motif. For both the precursors and P450s, an e-value of 5 and a threshold of 8 were set.

The generated sequence similarity network was then visualized using Cytoscape 3.10.1,^53^ and annotations were added using the AutoAnnotate 1.4 plugin.^54^

#### Phylogenetic analysis of atropopeptide-modifying P450s

To build a maximum likelihood tree of all putative atropopeptide-modifying cytochrome P450s, all P450s detected by Atropofinder that had a Corefinder hit were aligned using ClustalW version 1.2.3 ^46^ with the default settings. The alignment was trimmed using ClipKIT version 1.4.0 ^55^ using the default settings. The tree was assembled using IQ-TREE multicore version 1.6.12 ^56^ with default settings and visualized using iTOL version 5.^57^ To perform the co-occurrence analysis, the RODEO2 web tool^17^ was used with all putative P450s determined by AtropoFinder as input. The GC content of the P450 was compared to the average GC content of the genome of the species queried from the NCBI taxonomy browser.^58^

#### BiG – SCAPE analysis

The gene cluster family analysis was conducted using BiG-SCAPE.^40^ The tool was run from the command line using the following parameters: ∼/bin/run_bigscape. Output_bigscape/ --include_gbk_str * --include_singletons --mix --cutoffs 0.1 0.25 0.5 0.7 0.8 1.0 --mibig -v.

#### Protein structure modeling

Protein structure modeling was performed using AlphaFold 2 ^39^ in multimer mode with standard parameters. The structures were visualized in pymol.^59^

#### Culture conditions

*Escherichia coli* strains were cultured in Luria–Bertani (LB) broth or on LB agar (10 g per L tryptone, 5 g per L yeast extract, 10 g per L NaCl, 15 g per L agar) overnight at 37 °C. Tryptic soy broth medium (TSB) was used to grow *Streptomyce*s strains for routine applications and Mannitol-soy flour agar (MS agar) (20 g per L mannitol, 20 g per L soya flour, 20 g per L agar, and 10 mM MgCl_2_) or ISP2 (4.0 g per L glucose, 4.0 g per L yeast extract and 10.0 g per L malt extract and 20 g per L agar) was used for sporulation. *Streptomyces* strains were grown at 30 °C for 4–8 days. For the cultivation of plasmid containing clones, the medium was supplemented with the appropriate antibiotics at the following final concentrations: 50 μg per mL kanamycin and 50 μg per mL apramycin.

#### Molecular cloning

All polymerase chain reactions (PCRs) were conducted on a C1000 Touch Thermal Cycler (Bio-Rad) using Q5 High Fidelity DNA polymerase according to the manufacturer's instructions. PCR reactions were performed using the following conditions: initial denaturation (10 min, 98 °C) followed by 30–35 cycles of denaturation (20 s, 98 °C), annealing (30 s, 53–72 °C, depending on the melting temperature of primers) and elongation (based on PCR product length 30 s/1 kb, 72 °C); and final extension (10 min, 72 °C). DNA fragments from restriction digestions or PCR reactions were purified by agarose gel electrophoresis and isolated using the kits mentioned above. All plasmids constructed were verified by Sanger sequencing.

#### *jum* BGC

The plasmid backbone pUWL201-oriT was PCR amplified with the primer pair pUWL_del_fwd and pUWL_del_rev to minimize space between ribosomal binding site and multiple cloning site (Table S8†). The PCR product was subjected to KLD treatment and the resulting plasmid pUWL201-OriT-deletion was amplified with the primer pair bb_jumo_fwd and bb_jumo_rev (Table S8†). A 1252 bp DNA fragment containing the jumorubin BGC from *Streptomyces jumonjinensis* DSM 747 was PCR amplified with the primer pair jumo_fwd and jumo_rev (Table S8†) using extracted genomic DNA as a template. The two resulting DNA fragments were assembled using HiFi assembly to generate pUWL201-OriT-*jum*.

#### *sva* BGC

The sva BGC was amplified from *S. varsoviensis* DSM 40346 genomic DNA using primer pairs Sva_fwd and Sva_2_rev (Table S8†). The plasmid backbone (pUWL201-OriT) was linearized by PCR using primer pairs pUWL-OriT_fwd and pUWL_RBS_Rev (Table S8†). The two resulting DNA fragments were assembled using HiFi assembly to generate pUWL201-OriT-Sva.

To insert a ribosomal binding site in front of svaB, the plasmids pUWL201-OriT-Sva was used as template for reverse amplification by PCR using primer pairs Svar_RBS_CYP450_Fw and Svar-CYP450_Rev (Table S8†) to afford the linearized plasmid, which was then incubated with KLD mix. 1 μL of the KLD reaction mix was transformed into *E. coli* DH5α. The generated plasmid was named pUWL201-OriT-SvaR.

#### *sca* BGC

A 1364 bp DNA fragment containing the *sca* BGC from the *Embleya scabrispora* DSM 41855 was PCR amplified using the primer pairs sca-pp-F/R (Table S8†) with genomic DNA as template. The pUWL201-OriT backbone was linearized by PCR using primer pairs pUWL201-OriT-F/R (Table S8†). The two resulting DNA fragments were assembled using HiFi assembly to generate pUWL201-OriT-*sca* after sequencing confirmation.

#### *lau* BGC

A 2436 bp DNA fragment containing the *lau* BGC from *S. laurentii* ATCC 31255 was first cloned into pIJ10257. The *lau* BGC fragment was amplified from genomic DNA with primer pairs TrypLaurentii_Fwd_NdeI and TrypLaurentii_RV_XhoI (Table S8†). The plasmid pIJ10257 was linearized with *Nde*I and *Xho*I. The linearized pIJ10257 and the amplified *lau* BGC fragment were ligated using T4 DNA ligase to generate pIJ10257-*lau* after sequencing confirmation.

To clone *lau* BGC into pUWL201-OriT, the plasmid pUWL201-OriT was linearized with *Kpn*I and *Bam*HI. The fragment containing the *lau* BGC was excised from pIJ10257-*lau* using *Kpn*I and *Bam*HI. The linearized pUWL201-OriT and *lau* BGC fragments were ligated using T4 DNA ligase to generate pUWL201-OriT-*lau* after sequencing confirmation.

#### Coexpression of *lauA* and *lauB1*

To coexpress the *lauA* and *lauB1* genes, the plasmid pUWL201-OriT-*lau* was amplified using the prime pairs lauAB1-F/R (Table S8†) to afford the linearized plasmid, which was then treated with KLD mix. 1 μL of the KLD reaction mix was transformed into *E. coli* DH5α. The generated plasmid was named pUWL201-OriT-lauA + lauB1.

#### Coexpression of *lauA* and *lauB2*

To coexpress the *lauA* and *lauB2* genes, the fragment containing *lauA* and fragment containing *lauB2* were PCR amplified with the primer pairs lauA-F/R and lauB-F/R (Table S8†), respectively, using plasmid pUWL201-OriT-*lau* as template. The pUWL201-OriT backbone was linearized by PCR using primer pairs pUWL-LF/LR (Table S8†). The three resulting DNA fragments were assembled using HiFi assembly to generate plasmid pUWL201-OriT-lauA + lauB2.

#### Heterologous expression of selected BGCs

For heterologous expression of *jum* BGC, the plasmid pUWL201-OriT-*jum* was transformed into the heterologous host *S. albus* J1074 by conjugation. Briefly, the plasmid pUWL201-OriT-*jum* was transformed into *E. coli* ET12567/pUZ8002 to afford ET12567/pUZ8002/pUWL201-OriT-*jum* as the donor strain. 20 mL of LB medium was prepared to culture the donor strain at 37 °C, 180 rpm with appropriate antibiotics to an OD_600_ of 0.6–0.8. The cells were harvested, washed twice with 20 mL antibiotic-free LB broth and resuspended in 400 μL of LB medium. The *S.albus* J1074 was streaked on MS agar plates and cultivated at 30 °C for 5–7 days for sporulation. Then the spores of *S. albus* J1074 (recipient strain) were harvested, resuspended in 600 μL TSB medium and heated at 50 °C for 10 min. After heating, the spore suspensions were incubated at 30 °C for 0.5–1 h. The donor strain and the recipient strain were mixed and then diluted 1000 fold. After dilution, 200 μL of the mixed strains were spread onto MS agar plates containing 10 mM MgCl_2_. After incubation at 30 °C for 15–18 h, the plates were overlayed with apramycin (50 μg mL^−1^) and trimethoprim (100 μg mL^−1^) or apramycin (50 μg mL^−1^) and nalidixic acid (50 μg mL^−1^) solutions and incubated at 30 °C for 3–4 days or until exconjugants were visible. The exconjugants were individually picked and re-streaked onto MS agar or ISP2 agar plates containing apramycin (50 μg mL^−1^) and trimethoprim (100 μg mL^−1^) or apramycin (50 μg mL^−1^) and nalidixic acid (50 μg mL^−1^) and incubated at 30 °C. Three positive clones were randomly selected for small scale fermentation and subjected to metabolite analysis by LC-MS.

For heterologous expression of *sva* (Sva and SvaR), *sca*, *lau*, *lauA* + *lauB1* and *lauA* + *lauB2*, the corresponding plasmids pUWL201-OriT-Sva, pUWL201-OriT-SvaR, pUWL201-OriT-*sca*, pUWL201-OriT-*lau*, *pUWL201-OriT-lauA* + *lauB1*, pUWL201-OriT-*lauA* + *lauB2* were transformed into *S. albus* J1074 by conjugation as described above.

#### Small-scale fermentation and LC-MS analysis

For the small scale fermentation, the heterologous recombinant strains were streaked on MS agar plates with apramycin (50 μg mL^−1^) and cultivated at 30 °C for 5–7 days for sporulation. Then an aggregate of spores of the recombinant strain was collected and transferred into a 250 mL Erlenmeyer flask containing 50 mL of TSB medium with apramycin (50 μg mL^−1^) and incubated at 30 °C, 180 rpm for 6 days. On the fifth day, 5% (w/v) Diaion HP-20 resin (Sigma-Aldrich) was added to the culture. The resin was harvested by centrifugation (3900 rpm, 10 min) and extracted with 20 mL of acetone under sonication for 15 min. The extracts were dried under reduced pressure and dissolved in methanol for LC-MS analysis.

LC-MS measurements were carried out on an Ultimate 3000 LC system (Thermo Fisher) coupled to an AmaZonX (Bruker) electrospray ionization (ESI) mass spectrometer. Separation was achieved on a C18 column (ACQUITY UPLC BEH, 130 Å, 1.7 μm particle size, 2.1 × 100 mm, Waters) at a flow rate of 0.4 mL min^−1^ at 40 °C, using acetonitrile and Milli-Q water supplemented with 0.1% (v/v) formic acid in a gradient ranging from 5 to 95% acetonitrile over 16 min. HPLC-ESI-QTOF-MS analyses were conducted on an Ultimate 3000 LC system (Thermo Fisher) coupled to an Impact II QTOF mass spectrometer (Bruker). Separation was achieved on a C18 column (ACQUITY UPLC BEH, 130 Å, 1.7 μm particle size, 2.1 mm × 100 mm, Waters) at a flow rate of 0.4 mL min^−1^ at 40 °C, using acetonitrile and Milli-Q water supplemented with 0.1% (v/v) formic acid in a gradient ranging from 5 to 95% acetonitrile over 16 min. Data were acquired in positive mode at a scan range between 100 to 1200 *m*/*z* to detect atropopeptides. The software DataAnalysis 4.3 (Bruker) was used to evaluate the measurements.

#### Large-scale production and purification of atropopeptides

For large-scale production of jumorubin, seed cultures of *Streptomyces* strains carrying pUWL201-OriT-*jum* were prepared by fermentation in a culture tube containing 5 mL TSB medium with 50 μg mL^−1^ apramycin at 30 °C, 200 rpm, for 4 days. After incubation, 1 mL of the seed cultures was used to inoculate (6 × 100 mL) TSB medium containing 50 μg per mL apramycin in 500 mL Erlenmeyer flasks. After 3 days, 100 mL was used to inoculate (6 × 1000 mL) TSB medium containing 50 μg per mL apramycin in a 5000 mL Erlenmeyer flask. The cultures were incubated at 30 °C, 180 rpm, for 4 days. 5% (w/v) Diaion HP-20 resin (Sigma-Aldrich) was added to the culture and incubation was continued for one day. The Diaion HP-20 resin in the cultures was recovered by filtration using a metal sieve (40 mesh). The Diaion HP-20 resin was subsequently washed with H_2_O and then extracted twice with one culture volume of acetone. The crude extract was dried under reduced pressure.

The obtained crude extracts were fractionated by preparative chromatography (Büchi Pure C-850 Flash/Prep) using water and acetonitrile supplemented with 0.1% (v/v) formic acid as mobile phases A and B, respectively. Atropopeptides were purified from crude extracts using a Xbridge Prep C18 column (5 μm particle size, 250 × 19 mm, Waters) and eluted with a flow rate of 20 mL min^−1^ and a gradient from 10 to 45% solvent B over 40 minutes. Eluting compounds were detected with a UV-detector (254–400 nm). Fractions were screened for atropopeptides by LC-MS analysis, and the fractions containing the desired compounds were dried under reduced pressure, re-dissolved in methanol and processed further by semi-preparative HPLC on an Agilent 1260 Infinity II UV-Vis system, equipped with a phenyl–hexyl column (100 Å, 5 μm particle size, 250 × 10 mm, Phenomenex). Solvents used were Milli-Q water and acetonitrile as mobile phases A and B, respectively. Elution was achieved with a method containing three isocratic steps, 25% solvent B for 10 minutes, 28% solvent B for 20 minutes and 35% solvent B for 2 minutes using a flow rate of 5 mL min^−1^. After LC-MS analysis of the fractions, fractions containing the compound of interest were collected, dried under reduced pressure and re-purified on the same semi-preparative HPLC system. A gradient was set from 30 to 39% solvent B over 30 min and then 100% solvent B for 5 min, giving a total run time of 35 min, with a flow rate of 3 mL min^−1^. The purified compound was weighed (1.37 mg), dissolved in DMSO-*d*_6_ and then subjected to NMR analysis.

For large-scale production of varsorubin, seed cultures of *Streptomyces* strains carrying pUWL201-OriT-SvaR were prepared by fermentation in a culture tube containing 5 mL TSB medium with 50 μg mL^−1^ apramycin at 30 °C, 200 rpm, for 2–3 days. After incubation, 1 mL of the seed culture was used to inoculate (6 × 100 mL) TSB medium containing 50 μg per mL apramycin and 5% (w/v) Diaion HP-20 resin (Sigma-Aldrich) in 500 mL Erlenmeyer flasks. After 3 days, 100 mL was used to inoculate (6 × 1000 mL) mL TSB medium containing 50 μg mL^−1^ apramycin and 5% (w/v) Diaion HP-20 resin (Sigma-Aldrich) in a 5000 mL Erlenmeyer flask. The cultures were incubated at 30 °C, 180 rpm, for 4–6 days. The Diaion HP-20 resin in the TSB cultures was recovered by filtration with Miracloth (Milipore, MA, USA). The Diaion HP-20 resin was subsequently washed with H_2_O and then extracted twice with one culture volume of acetone. The crude extract was dried under reduced pressure. The obtained crude extracts were dissolved in DMSO and used to purify varsorubins by preparative HPLC on an Agilent 1260 Infinity II UV-Vis system, equipped with a XBridge BEH C18 OBD Prep Column (130 Å, 10 μm particle size, 30 mm x 250 mm, Waters). Solvents used were Milli-Q water and acetonitrile supplemented with 0.1% (v/v) formic acid as mobile phases A and B, respectively. A gradient was set from 10 to 45% solvent B over 35 min and then 100% solvent B for 5 min, giving a total run time of 40 min, with a flow rate of 20 mL min^−1^. The fractions containing the desired compounds were dried under reduced pressure, redissolved in DMSO and processed further by semi-preparative HPLC on an Agilent 1260 Infinity II UV-Vis system, equipped with a Luna phenyl–hexyl column (100 Å, 5 μm particle size, 250 × 4.6 mm, Phenomenex). Solvents used were Milli-Q water and acetonitrile supplemented with 0.1% (v/v) formic acid as mobile phases A and B, respectively. A gradient was set from 20 to 60% solvent B over 30 min and then 100% solvent B for 5 min, giving a total run time of 35 min, with a flow rate of 3 mL min^−1^. Fractions containing the desired compounds were subjected to LC-MS to check for purity. The purified compounds were weighed (1.6 mg for varsorubin B1 and 1.8 mg for varsorubin B2a) and redissolved in DMSO-*d*_6_. After that, the purified compounds were subjected to NMR analysis (1D and 2D NMR).

For the large-scale fermentation of *S. albus*/pUWL201-OriT-*sca* leading to production of scabrirubin, 6 L cultures were prepared as follows: the spores collected from one MS agar plate were incubated into a 5 L Erlenmeyer flask containing 1 L of the TSB medium with apramycin (50 μg mL^−1^) and incubated on a rotary shaker (180 rpm) at 30 °C for 6 days. 5% (w/v) of sterilized resin (Diaion HP-20) was added into each flask after 4 days of incubation, and the flasks were incubated for another 2 days. 4 L cultures were prepared as follows: the spores collected from one MS agar plate were inoculated into five 1 L Erlenmeyer flasks containing 200 mL of the TSB medium with apramycin (50 μg mL^−1^). The incubation conditions were the same as described above. The resins from the 10 L cultures were harvested by filtration through a metal sieve (40 mesh). The harvested resins were washed with water, transferred to a separatory funnel and eluted with 5 L of acetone. After evaporation of the organic solvents under reduced pressure, the crude extracts were subjected to normal phase silica gel column chromatography (230–400 mesh) and eluted with CHCl_3_/CH_3_OH (1 : 0, 97 : 3, 95 : 5, 90 : 10, 8 : 1, 4 : 1, 2 : 1, 0 : 1, v/v, 600 mL) to yield 8 fractions (Frs.1–8). Fr.6 and Fr.7 were combined and separated by Sephadex LH-20, eluted with CHCl_3_/CH_3_OH (1 : 1, v/v) to obtain 4 sub-fractions (Fr.1.1 to Fr.1.4). Fr.1.2 was further separated by semi-preparative HPLC using a reverse-phase column (Luna phenyl–hexyl, 250 × 4.6 mm, 5 μm particle size, Phenomenex) with UV detection at 300 nm to afford compound 5 (3.5 mg) using the following gradient: solvent system (solvent A, water supplementing with 0.1% formic acid; solvent B, acetonitrile); 30–39% B (0–25 min), 39–100% B (25–26 min), 100% B (26–30 min), 100–30% B (30–31 min), 30% B (31–35 min); flow rate at 2.5 mL min^−1^. The purified compound was weighed (3.5 mg), redissolved in DMSO-*d*_6_ and subjected to NMR analysis.

For large-scale production of laurentirubin B, seed cultures of *Streptomyces albus* strains carrying pUWL201-OriT-Precursor-laurentii were prepared by fermentation in a culture tube containing 5 mL TSB medium with appropriate antibiotic(s) at 30 °C, 200 rpm, for 2–3 days. After incubation, 1 mL of the seed culture was used to inoculate (6 × 100 mL) TSB medium containing appropriate antibiotic(s) and 5% (w/v) Diaion HP-20 resin in 500 mL Erlenmeyer flasks. After 3 days, 100 mL was used to inoculate (6 × 1000 mL) mL TSB medium containing appropriate antibiotic(s) and 5% (w/v) Diaion HP-20 resin in a 5000 mL Erlenmeyer flask. The cultures were incubated at 30 °C, 180 rpm, for 4–6 days. The Diaion HP-20 resin in the cultures was recovered by filtration with Miracloth. The Diaion HP-20 resin was subsequently washed with H_2_O and then extracted twice with one culture volume of acetone. The crude extract was dried under reduced pressure. The obtained crude extracts were dissolved in DMSO and then purified by preparative HPLC on an Agilent 1260 Infinity II UV-Vis system, equipped with a XBridge BEH C18 OBD Prep Column (130 Å, 10 μm particle size, 30 mm × 250 mm, Waters). Solvents used were Milli-Q water and acetonitrile supplemented with 0.1% (v/v) formic acid as mobile phases A and B, respectively. A gradient was set from 10 to 45% solvent B over 35 min and then 100% solvent B for 5 min, giving a total run time of 40 min, with a flow rate of 20 mL min^−1^. The fractions containing the desired compound were dried under reduced pressure, redissolved in DMSO and processed further by semi-preparative HPLC on an Agilent 1260 Infinity II UV-Vis system, equipped with a Luna phenyl–hexyl column (100 Å, 5 μm particle size, 250 × 4.6 mm, Phenomenex). Solvents used were Milli-Q water and acetonitrile supplemented with 0.1% (v/v) formic acid as mobile phases A and B, respectively. A gradient was set from 20 to 60% solvent B over 30 min and then 100% solvent B for 5 min, giving a total run time of 35 min, with a flow rate of 3 mL min^−1^. Fractions containing the desired compound were dried under reduced pressure. The purified compound was weighed (1.6 mg), redissolved in DMSO-*d*_6_ and subjected to NMR analysis.

#### Physical and spectroscopic data of isolated compounds

Varsorubin B1 (3): yield 1.6 mg; yellow powder; ^1^H and ^13^C NMR data, see Table S2;† HRMS (ESI-QTOF) *m*/*z* [M + H]^+^ calcd for C_42_H_47_N_8_O_8_^+^ 791.3517, found 791.3540.

Varsorubin B2a (4): yield 1.8 mg; orange powder; ^1^H and ^13^C NMR data, see Table S3;† HRMS (ESI-QTOF) *m*/*z* [M + H]^+^ calcd for C_42_H_49_N_8_O_8_^+^ 793.3668, found 793.3655.

Scabrirubin (5): yield 3.5 mg; white powder; ^1^H and ^13^C NMR data, see Table S4;† HRMS (ESI-QTOF) *m*/*z* [M + H]^+^ calcd for C_37_H_41_N_6_O_5_^+^ 649.3133, found 649.3132.

Laurentirubin B (6): yield 1.6 mg; orangewooder; ^1^H and ^13^C NMR data, see Table S5;† HRMS (ESI-QTOF) *m*/*z* [M + H]^+^ calcd for C_46_H_46_N_7_O_8_^+^ 824.3402, found 824.3401.

#### Structure elucidation

Varsorubin B1 (3) was isolated as a yellow powder. Its molecular formula was determined to be C_42_H_46_N_8_O_8_ based on a protonated ion at *m*/*z* 791.3540 (calcd for C_42_H_47_N_8_O_8_^+^, 791.3517, Δ +2.9 ppm) in HR-ESI-QTOF-MS data (Fig. S16†). Analysis of the ^1^H and ^13^C NMR spectra (Fig. S17 and S18†), with the aid of the HSQC spectrum (Fig. S19†), revealed the presence of 40 carbons including four carbonyl carbons, 11 sp^2^ protonated carbons, nine sp^2^ unprotonated carbons, a quaternary carbon, seven methines, six methylenes, and two doublet methyls. These signals and signals for exchangeable NH protons (*δ*_H_ 7.83, 7.21, 5.89) are characteristic for peptides (Table S2†). The ^1^H–^1^H COSY spectrum (Fig. S20†) showed a constituted spin system of signals at *δ*_H_ 7.21, 3.87, 1.57, 1.39, 1.33, 0.76, and 0.74, indicating the presence of a Leu residue. The typical AMPX spin system (*δ*_H_ 7.09, 6.78, 6.72, 6.64) (Table S2†) and the HMBC correlations from H-9 to C-11 and C-12 and from H-11 to C-8 (Fig. S21†) indicated the presence of a Trp residue (Trp2) that forms pyrroloindoline by a C–N bond between C-11 and N-8 (Fig. S23†). Another constituted spin system (*δ*_H_ 7.83, 4.00, 2.36, 2.32, 1.90, 1.64) and the core peptide sequence obtained from genomic data suggested the presence of a Gln residue. The presence of C-28-substituted Tyr was determined by HMBC correlations from H-25 to C-23, C-26, C-27, and C-31, from H-27 to C-29 and C-31, from H-30 to C-26 and C-28, and from H-31 to C-29 (Fig. S23†). Furthermore, C-39-substituted Trp (Trp1) was determined based on COSY correlations of H-33/H-34 and H-40/H-41 as well as HMBC correlations from H-36 to C-37 and C-42, from H-41 to H-37 and H-39, and from H-38 to C-40 and C-42. The amino acid sequence of 3 was determined to be NH_2_-Trp1-Tyr-Gln-Trp2-Leu-CO_2_H based on the COSY and HMBC correlations (Fig. S23†), which is consistent with the amino acid sequence of the core peptide from *svaA2*. The C–C bond between C-39 of Trp-1 and C-10 of Trp-2 was determined by HMBC correlations from H-38 and H40 of Trp-1 to the quaternary carbon C-10 of Trp-2. In addition, the NOESY correlations of H-27/H-36 and H-27/H-33 (Fig. S22 and S23†) suggested the presence of a C–N bond between N-36 of Trp-1 and the quaternary carbon C-28 of Tyr. The stereochemistry of the α-carbons for each amino acid was deduced as l-configured based on the genomic data. The NOESY correlations of H-8/H-40, H-9/H40, H-9/H-41, and H-11/H-38 (Fig. S23†) suggest the relative configuration of the pyrroloindoline moiety to be (10S*,11R*) and the ansameric configuration^60^ of the bicyclic macrolactam ring in 3 to be *P*_ansa_. The ansameric configuration *P*_ansa_ of 3 is the same as that of “bridge-above”-type tryptorubin A.^14^

Varsorubin B2a (4a) was isolated as an orange powder. Its molecular formula was determined to be C_42_H_48_N_8_O_8_ based on a protonated ion at *m*/*z* 793.3655 (calcd for C_42_H_49_N_8_O_8_^+^, 793.3668, Δ −1.6 ppm) in HR-ESI-QTOF-MS data (Fig. S25†). The ^1^H and 2D NMR spectra (Fig. S26–S28, S30 and S31†) showed similarity to those of 3 except for the presence of the AA′XX′ spin system (*δ*_H_ 6.95, 6.63) for a Tyr residue, a secondary amine proton signal (*δ*_H_ 7.24) for Trp-1, and an amide proton signal (*δ*_H_ 6.95) for Trp-2, and the absence of a signal for H-11 of Trp-2. These differences suggested that 4a is a peptide analog of 3 without the pyrroloindoline moiety at Trp-2 and a C–N bond between N-36 of Trp-2 and C-28 of Tyr. The HMBC correlations from H-38 and H-40 to C-11 (Fig. S29†) indicated the presence of a C–C linkage between C-39 of Trp-1 and C-11 of Trp-2. The presence of this C–C bond was further confirmed by NOESY correlations of H-8/H-38, H-8/H-40, H-9/H-38, H-9/H-40, and H-9/H-41 (Fig. S32 and S33†). The stereochemistry of the α-carbons for each amino acid was deduced as l-configuration based on the genomic data. The axial chirality between C-39 of Trp-1 and C-11 of Trp-2 was not determined in this study.

Scabrirubin (5) was obtained as a white powder. Its molecular formula was determined to be C_37_H_40_N_6_O_5_ (*m*/*z* 649.3132 [M + H]^+^, calcd for C_37_H_41_N_6_O_5_^+^, 649.3133, Δ −0.15 ppm) by HR-ESI-QTOF-MS data (Fig. S35†), suggesting the index of hydrogen deficiency to be 21. Analysis of the ^1^H and ^13^C NMR spectra, with the aid of the HSQC spectrum (Table S4 and Fig. S36–S39†), revealed the presence of 37 carbons assignable to four carbonyl carbons, eight sp^2^ nonprotonated carbons, 14 sp^2^ methines, five sp^3^ methines, four sp^3^ methylenes, and two methyls. These signals and signals for exchangeable NH protons (*δ*_H_ 8.04, 7.81, 7.35) are characteristic for peptides. The presence of a Phe residue was determined based on the ^1^H–^1^H COSY correlations between H-23/23′ and H-24 and the HMBC correlations of H-22/H-24, H-20/H-22, and H-20/H-21 (Fig. S43†). A continuous spin system in the aromatic region (*δ*_H_ 7.66, 7.52, 7.19, 7.12), a singlet signal for an aromatic proton (*δ*_H_ 7.85), and unequivalent methylene signals (*δ*_H_ 3.24, 3.15) suggested the presence of a Trp residue (Trp-2). The ^1^H–^1^H COSY spectrum (Fig. S40†) showed another constituted spin system region including resonances for two terminal methyls (*δ*_H_ 7.81, 4.06, 1.68, 1.66, 1.36, 0.94, 0.91), indicating the presence of an Ile residue. Furthermore, the C-34-substituted Trp (Trp-1) was assigned based on the ^1^H–^1^H COSY correlations of NH-29/H-29 and H-31/H-32 along with HMBC correlations from H-27 to C-29 and C-30, and from H-31 to C-30, C-33, and C-35, from H-32 to C-34, and from H-33 to C-35 (Fig. S43†). The amino acid sequence of 5 was determined to be NH_2_-Trp-Phe-Ile-Trp-CO_2_H based on the HMBC correlations from amino protons to carbonyl carbons (Fig. S41†). The stereochemistry of the α-carbons for each amino acid was deduced as l-configured based on the genomic data. The HMBC correlation from H-5 of Trp-2 to a quaternary carbon C-34 of Trp-1 suggested the presence of a C–N bond between C-34 of Trp-1 and N-5 of Trp-2. This bond was further confirmed by the NOESY correlations between the H-10/H-33, H-5/NH-29 (Fig. S42†).

Laurentirubin B (6) was isolated as an orange powder. Its molecular formula of C_46_H_45_N_7_O_8_ was determined by a protonated ion at *m*/*z* 824.3401 (calcd for C_46_H_46_N_7_O_8_^+^, 824.3402, Δ −0.1 ppm) in HR-ESI-QTOF-MS data (Fig. S46†). The ^1^H and 2D NMR spectra were akin to those of tryptorubin B^16^ except for the absence of an aromatic proton H-17 at a Trp-2 and signals of a Tyr-2 residue detected at *δ*_H_ 7.45, 7.10, 7.00, and 6.58 (Table S5, Fig. S47–S50 and S52†). These NMR data combined with the genomic data suggested that the amino acid sequence of 6 is identical to tryptorubin B (NH_2_-Trp-Tyr-Ile-Trp-Tyr-CO_2_H), but the modification of the amino acid sequence is different. The formation of the pyrroloindoline moiety at a Trp-2 residue was assigned from the COSY correlation between NH-12 and a sp^3^ methine H-12 as well as the HMBC correlations from H-12 to an α-carbon C-9, a methylene carbon C-10, and a quaternary carbon C-11. The HMBC correlation of H-10/C-41, H-12/C-41, H-40/C-11, and H-42/C-11 indicated the presence of a C–C bond between a quaternary carbon C-11 of Trp-1 and a nonprotonated sp^2^ carbon C-41 of Trp-1 (Fig. S52†). The presence of a C–N bond between C-30 of Tyr-1 and N-38 of Trp-1 was assigned from the ^1^H–^15^N HMBC correlations of H-28/N-38 and H-38/N-38 (Fig. S53†). This assignment was further confirmed by the NOESY cross peak between H-38 at Trp-1 and H-29 at Tyr-1 (Fig. S54†). The aryl ether bond between C-7 of Tyr-2 and C-17 of Trp-2 was deduced from the NOESY correlations of H-5′/H-10α and H-6/NH-12 (Fig. S53 and S54†), as well as asymmetric ^1^H and ^13^C signals for Tyr-2 residue (C-5, C-5′, C-6, C-6′) and the presence of a 1,2,3-trisubstituted aromatic ring in the pyrroloindoline moiety. The stereochemistry of the α-carbon for each amino acid residue was suggested as l-configuration based on the genomic data. Besides, the NOESY correlations of H-9/H-43, H-10/H-43, and H-12/H-40 (Fig. S53 and S54†) indicated the relative configuration of a quaternary carbon C-11 and an aminal carbon C-12 as (11S*,12R*) and the ansameric configuration of a bicyclic macrolactam ring as *P*_ansa_. To confirm the location of the aryl ether bridge and stereochemical configuration of the ansameric bismacrocycle, theoretical ^13^C NMR chemical shifts were calculated for possible conformers *P*_ansa_-6, *M*_ansa_-6, along with a possible isomer *P*_ansa_-6a that features an aryl ester bond between C-1 and C-17 instead of the aryl ether bond between C-7 and C-17 using the GIAO method. The calculated ^13^C NMR chemical shifts of *P*_ansa_-6a with the lowest values of the mean absolute error and mean squared error (Table S6†) were more favorable than those of *M*_ansa_-6 and *P*_ansa_-6a. Furthermore, the lowest energy conformer of *P*_ansa_-6 at the B3LYP/6-31G(d,p) level of theory (Fig. S57†) showed a rational geometry that was in agreement with the observed NOESY correlations in 6, except for the NOESY correlation of H-9/H-43. These DFT calculations supported the NMR analysis-based assignment of the ansameric configuration as *P*_ansa_, as well as the location of the aryl ether bond at C-7–C-17.

#### DFT calculations

3-Dimensional structures for *P*_ansa_-6, *P*_ansa_-6a, and *M*_ansa_-6 were modeled in the Spartan '18 V1.4.5 modeling software. The models were minimized using molecular mechanics with Spartan MMFF force field and subsequently subjected to conformational search using molecular mechanics again with the MMFF force field and using a Monte Carlo search method (specified by the option SEARCHMETHOD = MC). For each structure, the ten conformers with the lowest energy, as determined by MMFF, were selected for DFT calculations. All DFT calculations were performed with Gaussian 16 using the B3LYP function with a 6-31G(d,p) basis set and in the gas phase. Each conformer was energy minimized in Gaussian and checked for convergence with a frequency calculation. If the minimization had not converged, we repeated the minimization until convergence. We then performed an NMR GIAO calculation on the minimized structure. We used regression coefficients described by Konstantinov and Broadbelt^61^ to calculate the predicted chemical shifts for all carbons. We then averaged over the ten predicted spectra for each structure using a Boltzmann-weighted average. We determined and visualized the distances in the lowest energy structures corresponding to key NOESYs using Pymol.

#### Antimicrobial assay

Disk diffusion assays were performed to determine the antimicrobial activity of amyxirubin B (2), varsorubin B1 (3), varsorubin B2a (4a) and scabrirubin (5). The ESKAPE pathogens (*Enterococcus faecium*, *Staphylococcus aureus*, *Klebsiella pneumonia*, *Actinetobacter baumannii*, *Pseudomonas aeruginosa*, *Enterobacter cloacae*) were cultured in LB medium at 37 °C (130 rpm), *Arthrobacter pascens* DSM 20545 was cultured in LB medium at 30 °C (130 rpm) and *Candida albicans* CAF4-2 was cultured in potato dextrose broth (PDB) at 32 °C (130 rpm) to an OD_600_ value of ∼0.6. Cultures were mixed with LB agar or potato dextrose agar (PDA) in a ratio of 1 : 100 to prepare the agar plates. Compounds were dissolved in DMSO at a concentration of 2.56 mg mL^−1^. Trimethoprim and ampicillin were used as positive controls for the antibacterial assays. Nystatin and cycloheximide were used as positive control for the antifungal assays. 6 mm paper disks were loaded with 5 μL of each compound, respectively, and then placed onto the agar plates. The plates were incubated at 37 °C for 16 h (ESKAPE pathogens) or for 24 h (*C. albicans* CAF-2), or at 30 °C for one to five days (*A. pascens* DSM 20545). The antimicrobial activity was determined by the size of the zone of inhibition.

## Data availability

All code and raw data pertaining to the machine learning analyses presented in this study can be accessed at the following GitHub repository https://github.com/FriederikeBiermann/AtropoFinder and is publicly available as of the date of publication. Furthermore, all biosynthetic gene clusters (BGCs) and associated compounds derived from this research are available in the ESI† in the form of Genbank files and have been submitted to the MiBIG (Minimum Information about a Biosynthetic Gene Cluster) database for public access and reference. NMR spectra can be found in the ESI.† The raw data used to create Fig. 3 can be found in the ESI files (BGC_data_only_positives.csv, atropopeptide_p450s_newick.txt).† All AtropoFinder results can be found in the ESI file BGC_data.csv.†

## Author contributions

FB, PN, EJNH conceptualized the project, FB developed AtropoFinder and CoreFinder, performed analysis of atropopeptide BGCs, and conducted modeling studies, BT, MB, PN, YD designed and constructed plasmids, conducted heterologous expression studies, and isolated compounds, BT, YK, RU analyzed NMR data, ASW performed DFT calculations, BT conducted bioactivity assays, FB, BT, YK and EJNH wrote manuscript with contributions from all co-authors.

## Conflicts of interest

There are no conflicts to declare.

## Supplementary Material

SC-OLF-D4SC03469D-s001

SC-OLF-D4SC03469D-s002
