# Supplementary material for: Exploration, expansion and definition of the atropopeptide family of ribosomally synthesized and posttranslationally modified peptides
Source: Chem Sci. 2024 Sep 10;15(42):17506–23. doi: 10.1039/d4sc03469d (PMC11450843; doi:10.1039/d4sc03469d)
Supplement: SC-015-D4SC03469D-s001 [file SC-015-D4SC03469D-s001.pdf]

Supplementary Information (SI) for Chemical Science.
This journal is © The Royal Society of Chemistry 2024

Exploration, expansion and definition of the atropopeptide family of ribosomally synthesized and posttranslationally modified peptides

Friederike Biermann[a,b], Bin Tan[a,b], Milena Breitenbach[a,b], Yuya Kakumu[a,b], Pakjira Nanudorn[a,b], Yoana Dimitrova[a], Allison S. Walker[d,e], Reiko Ueoka[f], Eric J. N. Helfrich[a,b,c*]

[a] Institute for Molecular Bio Science
Goethe University Frankfurt, Max-von-Laue Strasse 9, 60438 Frankfurt am Main, Germany
[b] LOEWE Center for Translational Biodiversity Genomics (TBG)
Senckenberganlage 25, 60325 Frankfurt am Main, Germany
[c] Senckenberg Gesellschaft für Naturforschung
Senckenberganlage 25, 60325 Frankfurt am Main, Germany
[d] Department of Chemistry
Vanderbilt University, Stevenson Center 7330, Nashville, TN 37240, USA
[e] Department of Biological Sciences
Vanderbilt University, VU Station B, Box 35-1634, Nashville, TN 37235, USA
[f] School of Marine Biosciences
Kitasato University 1-15-1 Kitasato, Minami-ku, Sagamihara, Kanagawa, 252-0373, Japan

*corresponding author: Eric J. N. Helfrich (E-mail: eric.helfrich@bio.uni-frankfurt.de)

Table of contents

Detailed description of AtropoFinder S6

Detailed description of CoreFinder S7

Comparison of AtropoFinder to existing bioinformatic tools S7

Figure S1 Overview over characterized peptide modifications in non-ribosomal peptides and ribosomally synthesized and posttranslationally modified peptides. S8

Figure S2 Featurisation of the amino acid sequences of P450s to feed into the machine learning classifier. S9

Figure S3 Simplified amino acid code that reflects the physicochemical properties of the proteinogenic amino acids. S10

Figure S4 Confusion matrix of atropopeptide-modifying P450s for conditional random forest classifier utilized for the prediction of atropopeptide-modifying P450s on the internal validation set. S11

Figure S5 Comparison of BGC predictions of different state-of-the-art genome mining tools on a nucleotide sequence containing the atropopeptide BGCs identified by AtropoFinder. S12

Figure S6 Meme conserved motif search. S13

**Figure S7**	Metrics of the AlphaFold predicted model of precursor peptide and P450 encoded in the *trp* BGC.	S14
**Figure S8**	BiG-SCAPE analysis of all putative atropopeptide-containing gene clusters (red) and all characterized BGC deposited to the MiBIG database (blue) at threshold of 0.5.	S15
**Figure S9**	Sequence similarity network of all putative atropopeptide precursors and their corresponding core peptide sequences.	S16
**Figure S10**	Phylogenetic distribution of putative atropopeptide clusters.	S17
**Figure S11**	Extracted ion chromatograms of amyxirubin B and jumorubin (**2**).	S17
**Figure S12**	HPLC-ESI-QTOF-HRMS analysis of jumorubin (**2**).	S18
**Figure S13**	^{1}H NMR spectrum (600 MHz) of jumorubin (**2**) in DMSO-d_6.	S19
**Figure S14**	Extracted ion chromatogram of varsorubin B1 (**3**) and varsorubin B2 (**4**).	S20
**Figure S15**	Extracted ion chromatogram of varsorubin B1 (**3**) and varsorubin B2 (**4**).	S21
**Figure S16**	HPLC-ESI-QTOF-HRMS analysis of varsorubin B1 (**3**).	S22
**Figure S17**	^{1}H NMR spectrum (600 MHz) of varsorubin B1 (**3**) in DMSO-d_6.	S23
**Figure S18**	^{13}C NMR spectrum (150 MHz) of varsorubin B1 (**3**) in DMSO-d_6.	S24
**Figure S19**	HSQC spectrum (600 MHz) of varsorubin B1 (**3**) in DMSO-d_6.	S25
**Figure S20**	COSY spectrum (600 MHz) of varsorubin B1 (**3**) in DMSO-d_6.	S26
**Figure S21**	HMBC spectrum (600 MHz) of varsorubin B1 (**3**) in DMSO-d_6.	S27
**Figure S22**	NOESY spectrum (600 MHz) of varsorubin B1 (**3**) in DMSO-d_6.	S28
**Figure S23**	Structure elucidation of varsorubin B1 (**3**).	S29
**Figure S24**	Extracted ion chromatograms of (A) varsorubin B2b (**4b**), (B) varsorubin B2a (**4a**) and (C) varsorubin B1 (**3**).	S30
**Figure S25**	HPLC-ESI-QTOF-HRMS analysis of varsorubin B1 (**3**).	S31
**Figure S26**	^{1}H NMR spectrum (600 MHz) of varsorubin B2a (**4a**) in DMSO-d_6.	S32
**Figure S27**	HSQC spectrum (600 MHz) of varsorubin B2a (**4a**) in DMSO-d_6.	S33
**Figure S28**	COSY spectrum (600 MHz) of varsorubin B2a (**4a**) in DMSO-d_6.	S34

**Figure S29**	Structure elucidation of varsorubin B2a (**4a**).	S35
**Figure S30**	HMBC spectrum (600 MHz) of varsorubin B2a (**4a**) in DMSO-$d_{6.}$	S36
**Figure S31**	TOCSY spectrum (600 MHz) of varsorubin B2a (**4a**) in DMSO-d_6.	S37
**Figure S32**	NOESY spectrum (600 MHz) of varsorubin B2a (**4a**) in DMSO-d_6.	S38
**Figure S33**	Key NOESY correlations of varsorubin B2a (**4a**) in DMSO-d_6.	S39
**Figure S34**	Extracted ion chromatogram of scabrirubin (**5**).	S40
**Figure S35**	HPLC-ESI-QTOF-HRMS analysis of scabrirubin (**5**).	S41
**Figure S36**	^{1}H-NMR spectrum (500 MHz) of scabrirubin (**5**) in DMSO-d_6.	S42
**Figure S37**	^{13}C-NMR spectrum (125 MHz) of scabrirubin (**5**) in DMSO-d_6.	S43
**Figure S38**	DEPT135 spectrum (125 MHz) of scabrirubin (**5**) in DMSO-d_6.	S44
**Figure S39**	HSQC spectrum (500 MHz) of scabrirubin (**5**) in DMSO-d_6.	S45
**Figure S40**	COSY spectrum (500 MHz) of scabrirubin (**5**) in DMSO-d_6.	S46
**Figure S41**	HMBC spectrum (500 MHz) of scabrirubin (**5**) in DMSO-d_6.	S47
**Figure S42**	NOESY spectrum (500 MHz) of scabrirubin (**5**) in DMSO-d_6.	S48
**Figure S43**	Structure elucidation of scabrirubin (**5**).	S49
**Figure S44**	Resequencing of *lau* BGC.	S50
**Figure S45**	Extracted ion chromatogram of pentapeptide laurentirubin B (**6**) and hexapeptide	S50
**Figure S46**	HPLC-ESI-QTOF-HRMS analysis of laurentirubin B (**6**).	S51
**Figure S47**	^{1}H NMR spectrum(600 MHz) of laurentirubin B (**6**) in DMSO-d_6.	S52
**Figure S48**	HSQC spectrum (600 MHz) of laurentirubin B (**6**) in DMSO-d_6.	S53
**Figure S49**	COSY spectrum (600 MHz) of laurentirubin B (**6**) in DMSO-d_6.	S54
**Figure S50**	TOCSY spectrum (600 MHz) of laurentirubin B (**6**) in DMSO-d_6.	S55
**Figure S51**	Structure of laurentirubin B (**6**).	S56
**Figure S52**	^{1}H–^{13}C HMBC spectrum (600 MHz) of laurentirubin B (**6**) in DMSO-d_6.	S57

**Figure S53**	^{1}H–^{15}N HMBC spectrum of laurentirubin B (**6**) in DMSO-d_6	S58
**Figure S54**	NOESY spectrum (600 MHz) of laurentirubin B (**6**) in DMSO-d_6.	S59
**Figure S55**	Key NOESY of laurentirubin B (**6**) in DMSO-d_6.	S60
**Figure S56**	*In vivo* functional characterization of P450s in *lau* BGC.	S60
**Figure S57**	Antibacterial assay of compounds **2**, **3**, **4a** and **5** against *Pseudomonas aeruginose.*	S61
**Figure S58**	The lowest energy conformer of P_{ansa}-**6a**, M_{ansa}-**6**, and P_{ansa}-**6** calculated at B3LYP/6-31G(d,p) level of theory. The values show distance (Å) of protons which showed NOESY correlations in **6**. Protons not involved in one of the NOESY correlations are hidden.	S61
**Table S1**	Detailed metrics of final classifiers for differentiating atropopeptide-modifying P450s (class 1) from P450s modifying any other substrate (class 0).	S62
**Table S2**	^{1}H and ^{13}C NMR Data for varsorubin B1 (**3**) in DMSO-d_6.	S63
**Table S3**	^{1}H and ^{13}C NMR Data for varsorubin B2a (**4a**) in DMSO-d_6.	S64
**Table S4**	^{1}H and ^{13}C NMR Data for scabrirubin (**5**) in DMSO-d_6.	S65
**Table S5**	^{1}H and ^{13}C NMR Data for laurentirubin (**6**) in DMSO-d_6.	S66
**Table S6**	Comparison of experimental and calculated ^{13}C NMR chemical shifts for **6**	S67
**Table S7**	Strains and plasmids used in this study	S68
**Table S8**	Primers used in this study	S69

**Supplementary data file S1**	Sequence alignment of all identified putative atropopeptide precusors. The names of the records are the GIs (sequence identifiers) from NCBI of the nucleotide sequences they have been found in.	precusor_alignment.fasta
**Supplementary data file S2**	Sequence similarity network of all putative atropopeptide precursors and their corresponding core peptide sequences.	precursor_peptides_SNN.xgmml

**Supplementary data file S3**	BiG-SCAPE analysis of all putative atropopeptide-containing gene clusters (red) and all characterized BGC deposited to the MiBIG database (blue) at threshold of 0.5.	bigscape_MiBIG_SNN.xgmml
**Supplementary data file S4**	Video of the AlphaFold2 multimer protein model of WP_007820080.1 and the tryptorubin A precursor peptide visualized from different angles. The leader peptide, KSLK motif, core peptide, and cytochrome P450 are depicted in green, blue, red, and gray, respectively.	P450_precursor_movie.mp4
**Supplementary data file S5**	Fasta file including all P450 sequences used as the positive training data set for the random forest classifier	atropopeptide_p450s_query.fasta
**Supplementary data file S6**	Table including information on atropopeptide BGCs, contains also AtropoFinder hits without a CoreFinder hit	BGC_data.csv
**Supplementary data file S7**	Table including information on atropopeptide BGCs	BGC_data_only_positives.csv
**Supplementary data file S8**	P450 pyhlogenetic tree in newick format	atropopeptide_p450s_newick.txt
**Supplementary data file S9**	Concatenated fasta used to compare AtropoFinder to other genome mining tools	positive_BGCs_for_comparison_with_other_tools.fasta
**Supplementary data file S10**	Genbank file containing the *sca* genecluster	NZ_KB889561_Scabrirubin.gb
**Supplementary data file S11**	Genbank file containing the *jum* genecluster	NZ_VCLA01000160_Amyxirubin.gb
**Supplementary data file S12**	Genbank file containing the *var* genecluster	NZ_JOBF01000044_Varsorubin.gb
**Supplementary data file S13**	Genbank file containing the *lau* genecluster	AP017424_Laurentirubin.gb

Supplementary Figures and tables

Detailed description of AtropoFinder

AtropoFinder is a Jupyter notebook-based script designed to handle the data preprocessing and machine learning to identify P450s involved in atropopeptide modification.

The primary input for AtropoFinder consists of amino acid sequence alignments of query P450s in fasta format. The first objective is to featurize these sequences to transform the raw amino acid sequences into numerical features that can be inputted into the machine learning algorithm (Figure S2). To map the specific features to functional regions of the P450, the alignments were prepared by aligning the query P450 sequences against a reference P450 for which functional regions have been annotated. The alignments are separated into functionally annotated regions based on insights from the annotations of the reference sequence. This method results in fragments of each P450 sequence associated with a specific functional region of the P450 (Methods, Data preprocessing). To prevent overfitting and focus less on sequence homology and more on binding properties of the amino acids, sequences are transformed into a simplified amino acid code that groups amino acids with similar physio-chemical properties under one character (Figure S3, Methods, Data preprocessing).

The next phase is the featurization, wherein overlapping occurrences of k-mer motifs with a length of four are counted within the fragmented sequences. To reduce the number of features and target atropopeptide-specific features, only k-mer motifs are considered that occur in at least half of the P450s of the atropopeptide training dataset (Figure S2 and Methods, Training of the classifier and hyperparameter optimization).

The machine learning algorithm utilizes a Random Forest classifier trained in advance on the atropopeptide training data set (Methods, Training data set assembly and Training of the classifier and hyperparameter optimization) to classify the sequences based on the features calculated in the step before. This classifier was selected based on its performance metrics, particularly its f1 score for atropopeptide P450s and is implemented in scikit-learn (Methods, Training of the classifier and hyperparameter optimization).

Atropofinder outputs a CSV table of all results, as well as a fasta file containing all P450s predicted to be atropopeptide-modifying enzymes.

Detailed description of CoreFinder

CoreFinder functions as a command-line tool designed to process fasta files of peptides, utilizing their accession numbers in the header for identification. CoreFinder's main functionality is to search for occurrences of genes coding these non-redundant proteins in the NCBI database using NCBI Entrez and subsequently analyze the genomic regions around these genes.

During its operation, CoreFinder identifies potential genes encoding precursor and core peptides. Open reading frames (ORFs) ranging between 10 to 40 amino acids that possess 'KSLK', 'RSLK', 'ESLK', 'KSRK', 'KPLK', or 'PSLK' in the precursor peptide are flagged. These ORFs should not coincide with existing coding sequences (CDS), with the exception of those categorized as "tryptorubin family RiPP precursor CDS". Overlapping precursor peptides ORFs are filtered to remove duplicates, keeping the shortest possible ORF. It is observed that the annotation of the exact start, and consequently, the length of precursor peptides encoding ORF can be difficult if multiple putative start codons are present. The output of the tool is a genbank file, capturing the 3kb region both upstream and downstream of the gene encoding the P450 of interest including the annotated precursor peptides. A more detailed description of the exact criteria can be found in the Methods Corefinder.

To aid its functionality, CoreFinder has several command-line options available to the user, including specifying input files, setting a genomic boundary for search, enabling dynamic core detection (instead of using the last 6 amino acids, it automatically uses the amino acid in front of W2 as the start of the core protein), and setting up an email used for the queries at NCBI Entrez. The tool also allows users to specify the desired output directory.

Comparison of AtropoFinder to existing bioinformatic tools

To compare the AtropoFinder results with existing state-of-the-art genome mining tools, all putative AtropoFinder BGCs were combined into one fasta file and used as input for different state-of-the-art genome mining tools. Using the antiSMASH 7 beta webserver and the antismash 6.1.1 webserver, 9 BGC regions were detected, all annotated as "indole", found based on the indole_PTase. The atropopeptide cytochrome p450 gene was labeled as "biosynthetic additional", whereas the precursor gene was labeled as "other gene". Based on the close inspection of the antiSMASH results, it can be assumed that antiSMASH did not detect the atropopeptide cluster, but rather a closeby indole BGC and thus annotated the atropopeptide BGC by chance. DeepBGC, which uses a neural network to distinguish BGC regions, annotated 108 putative gene clusters, usually labeled as "Polyketide-Terpene". These putative BGCs are much larger than the BGCs annotated by AtropoFinder, often containing multiple BGCs predicted by AtropoFinder. The tool GECCO labeled 38 regions as putative gene clusters most of which were not attributed to any natural product class (with the exception of one labeled "terpene"). The results of this query are displayed in Figure S5. A query with PRISM 4 showed no results, and a rodeo search with the tryptorubin A cytochrome P450 gene as a query only resulted in one result, the protein WP_007820080 of *Streptomyces* sp. SID8380 with no annotated precursors. In conclusion, all identified entities were seemingly discovered by chance and none of them were identified as a RiPP BGC. It has to be noted that the rule-based tools antiSMASH (with the exception of the RRE-finder implementation) and PRISM do not include rules for detecting atropopeptides and thus them not being able to detect atropopeptides was to be expected. They were included in the analysis to show the need for an additional genome mining tool for atropopeptides.

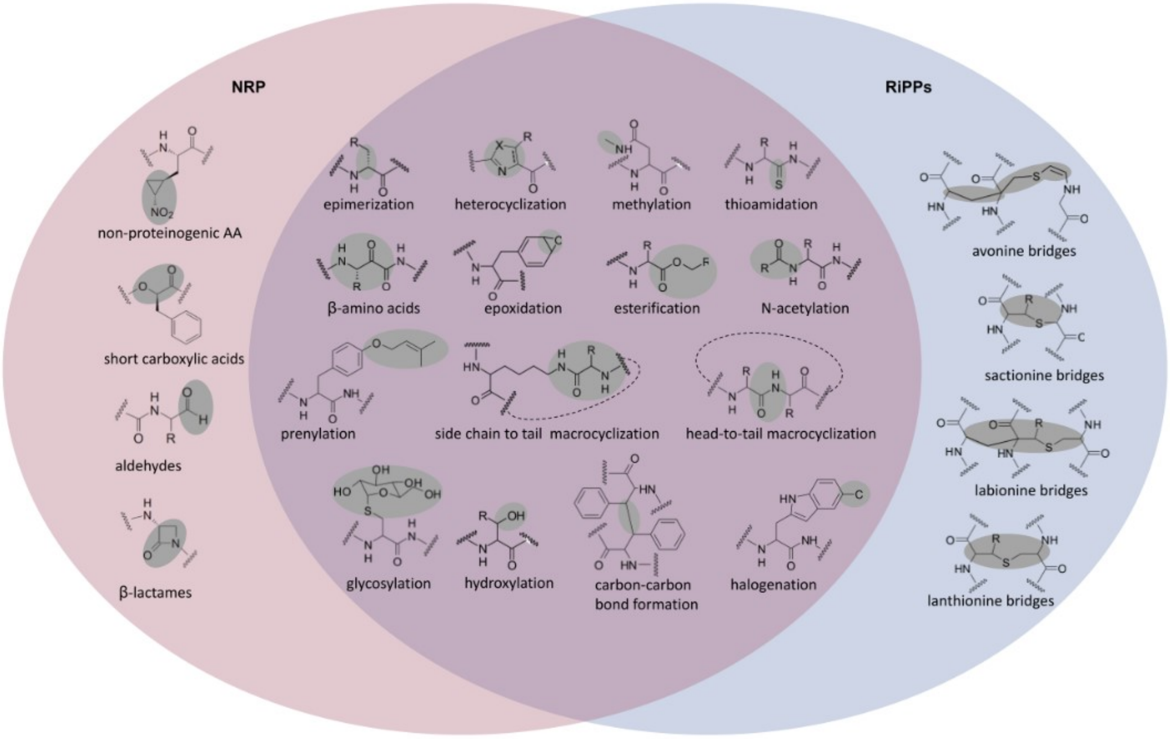

Figure S1. Overview over characterized peptide modifications in non-ribosomal peptides and ribosomally synthesized and posttranslationally modified peptides. The overview is intended to illustrate each type of modification with one example, rather than to catalog all characterized modifications. The overview does not aim to be exhaustive.

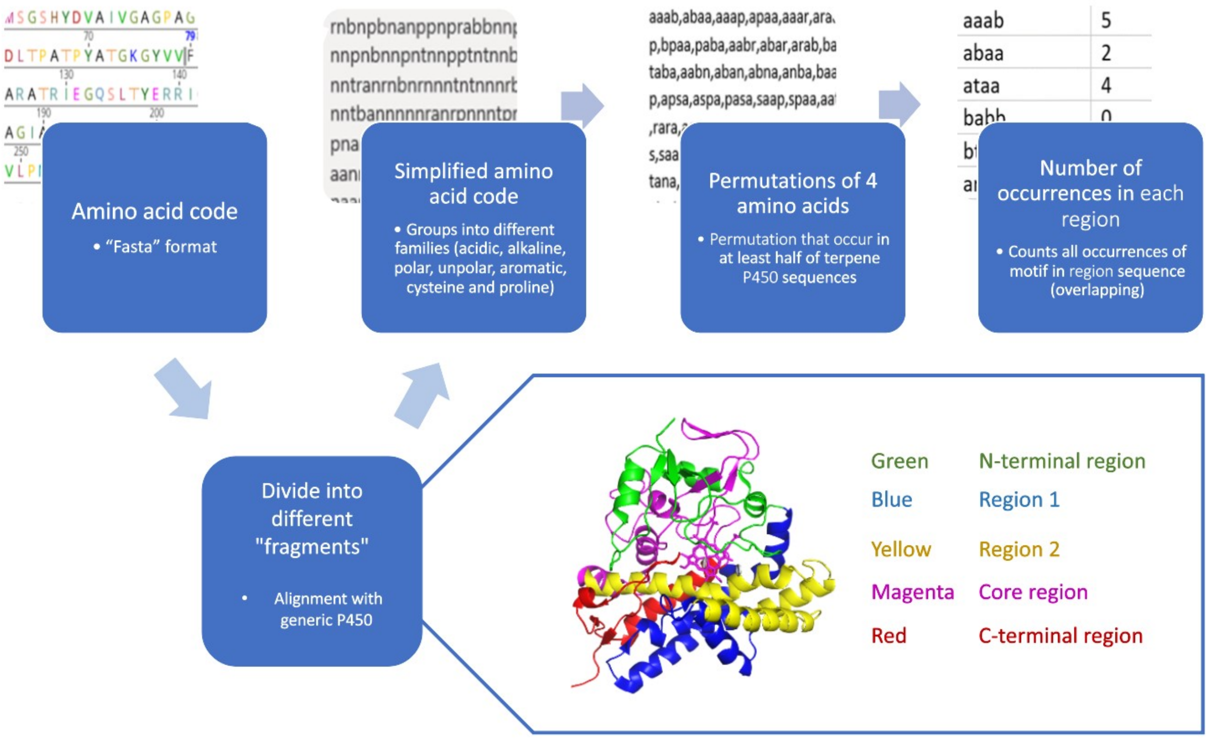

Figure S2. Featurisation of the amino acid sequences of P450s to feed into the machine learning classifier: First, the amino acid sequences are aligned against a reference P450 and split into fragments that resemble functional regions within the reference P450. Then, those fragmented amino acid sequences are translated into a simplified amino acid code (Figure S3). Subsequently, the number of occurrences of 4 amino acid long motifs (k-mers) is counted in each fragment and used as a feature.

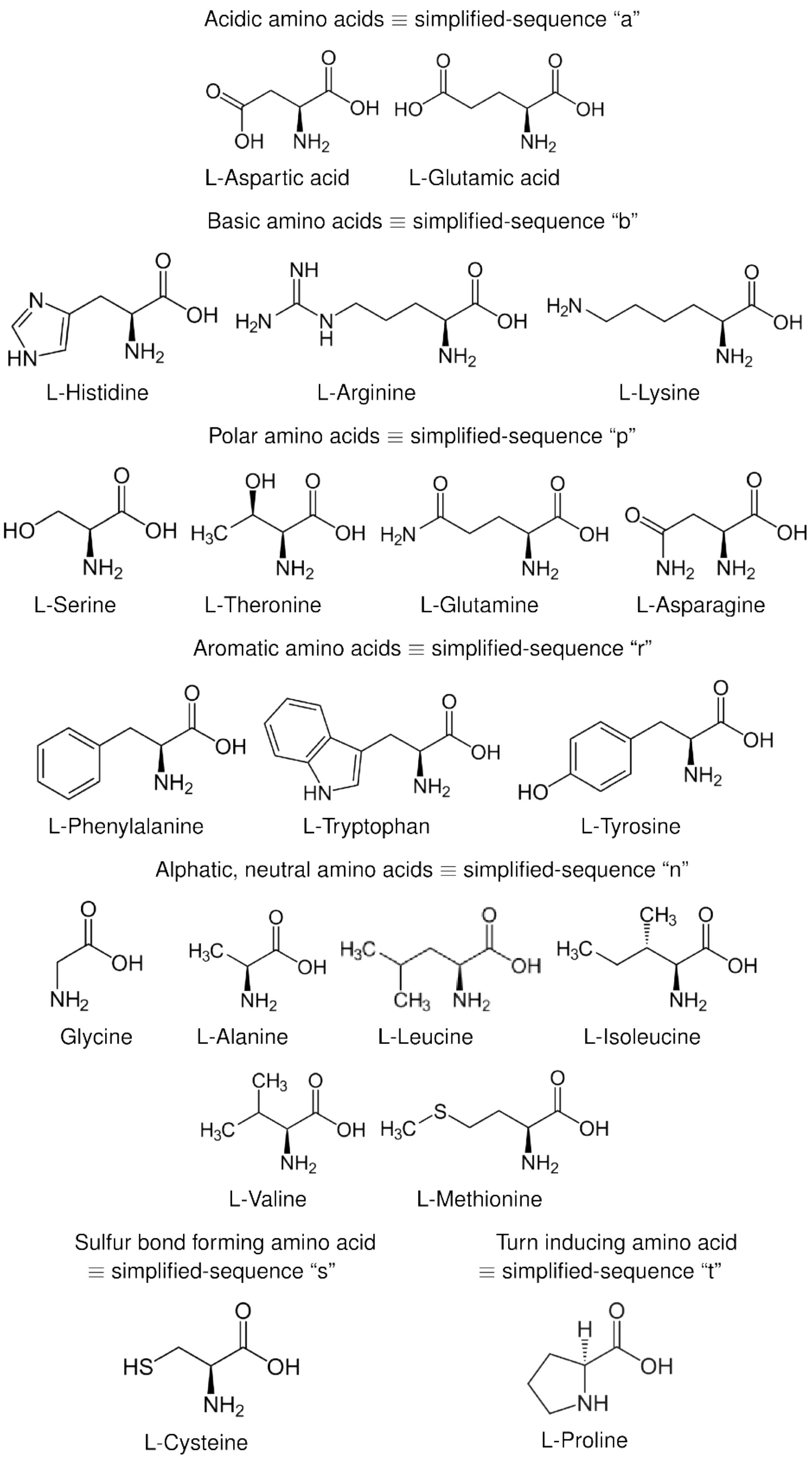

Figure S3. Simplified amino acid code that reflects the physicochemical properties of the proteinogenic amino acids.

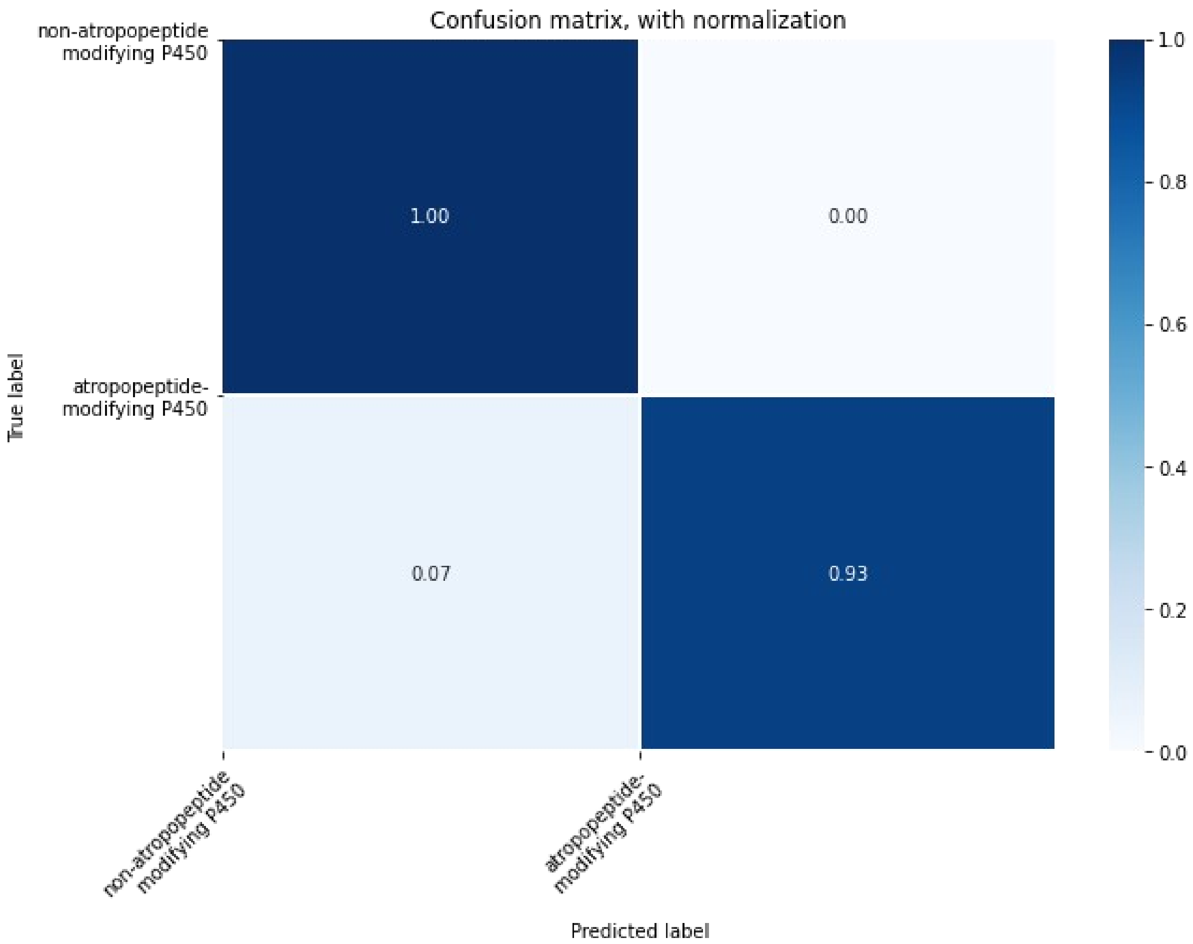

Figure S4. Confusion matrix of atropopeptide-modifying P450s for conditional random forest classifier utilized for the prediction of atropopeptide-modifying P450s on the internal validation set.

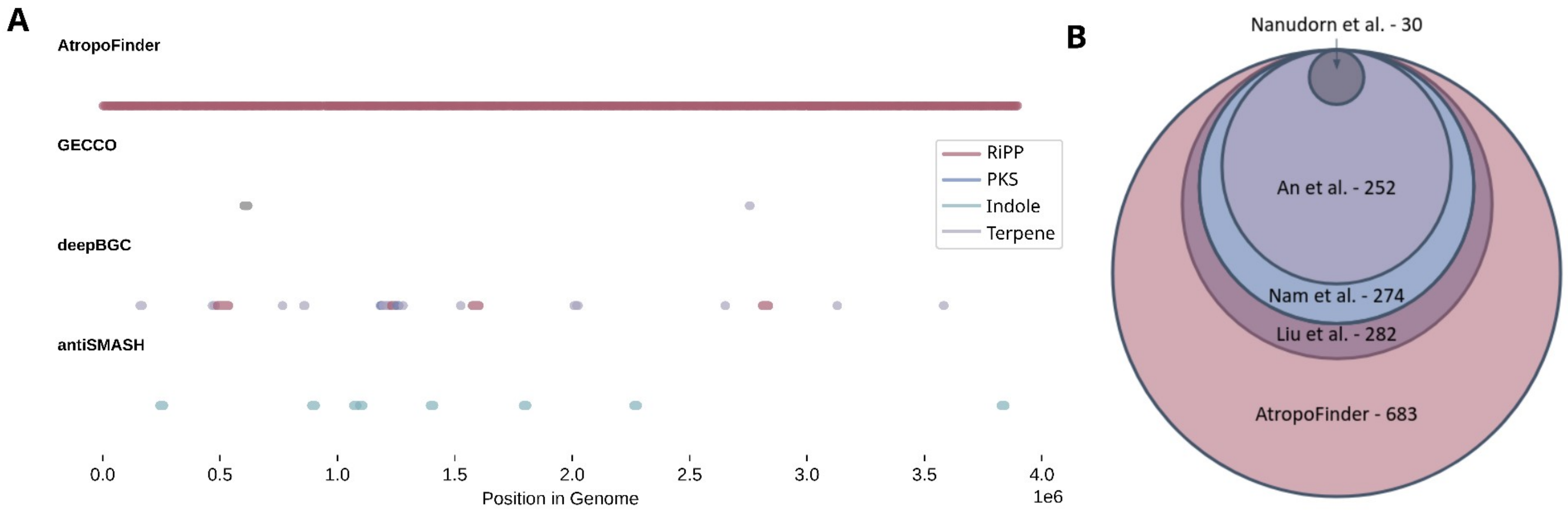

Figure S5 Comparison of AtropoFinder results with results obtained from state-of-the art genome mining tools and studies conducted with BLASTp/psi-BLAST. A) Comparison of BGC predictions of different state-of-the-art genome mining tools on a concatenated nucleotide sequence containing all atropopeptide BGCs identified by AtropoFinder.[1–3] Only deepBGC was able to identify a small number of putative RiPP BGCs. Since they span multiple BGCs identified by Atropofinder they are most likely artifacts. The indole BGCs identified by antiSMASH can most likely be attributed to flanking enzymes involved in indole synthesis, but antiSMASH did not identify the genes encoding the enzymes involved in atropopeptide biosynthesis. As neither one of those tools was programmed to detect atropopeptides, these results are not suprising, especially since GECCO and deepBGC predict BGCs based on the occurence of pfam domains. As the only feature defining atropopeptide BGCs is a cytochrome P450, they will most likely also not be able to detect them even if they were directly trained on atropopeptide BGCs. This hightlights the need for a tool that is able to differentiate tailoring enzymes within pfam domains for certain appllications. B) Quantitative comparison of atropopeptide BGCs identified with BLAST-based approaches and this study.[4–7]

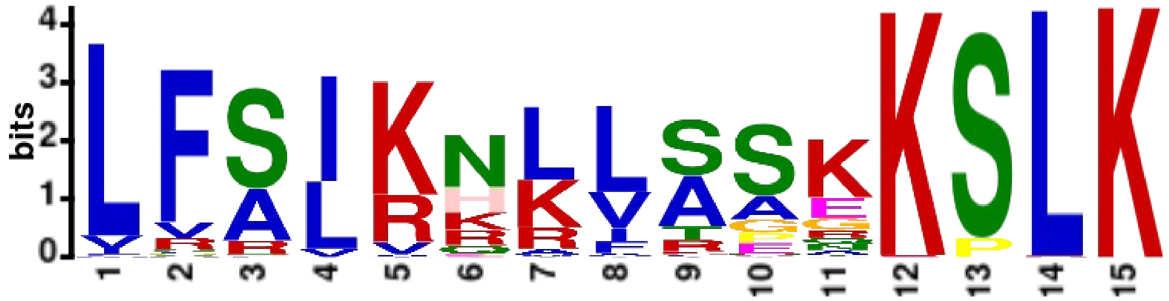

Figure S6. Meme[8] conserved motif search. The sequence logo represents a significant motif (E-value: 1.0e-2100) observed 338 times, spanning a width of 15 amino acids. The motif predominantly features the conserved “KSLK” sequence at the C-terminus, preceded by 9 variable or unspecified residues, as well as a conserved “LF” motif at the N-terminus. The height of each letter in the sequence logo corresponds to the frequency of the respective amino acid at that position, with taller letters indicating higher frequency and conservation.

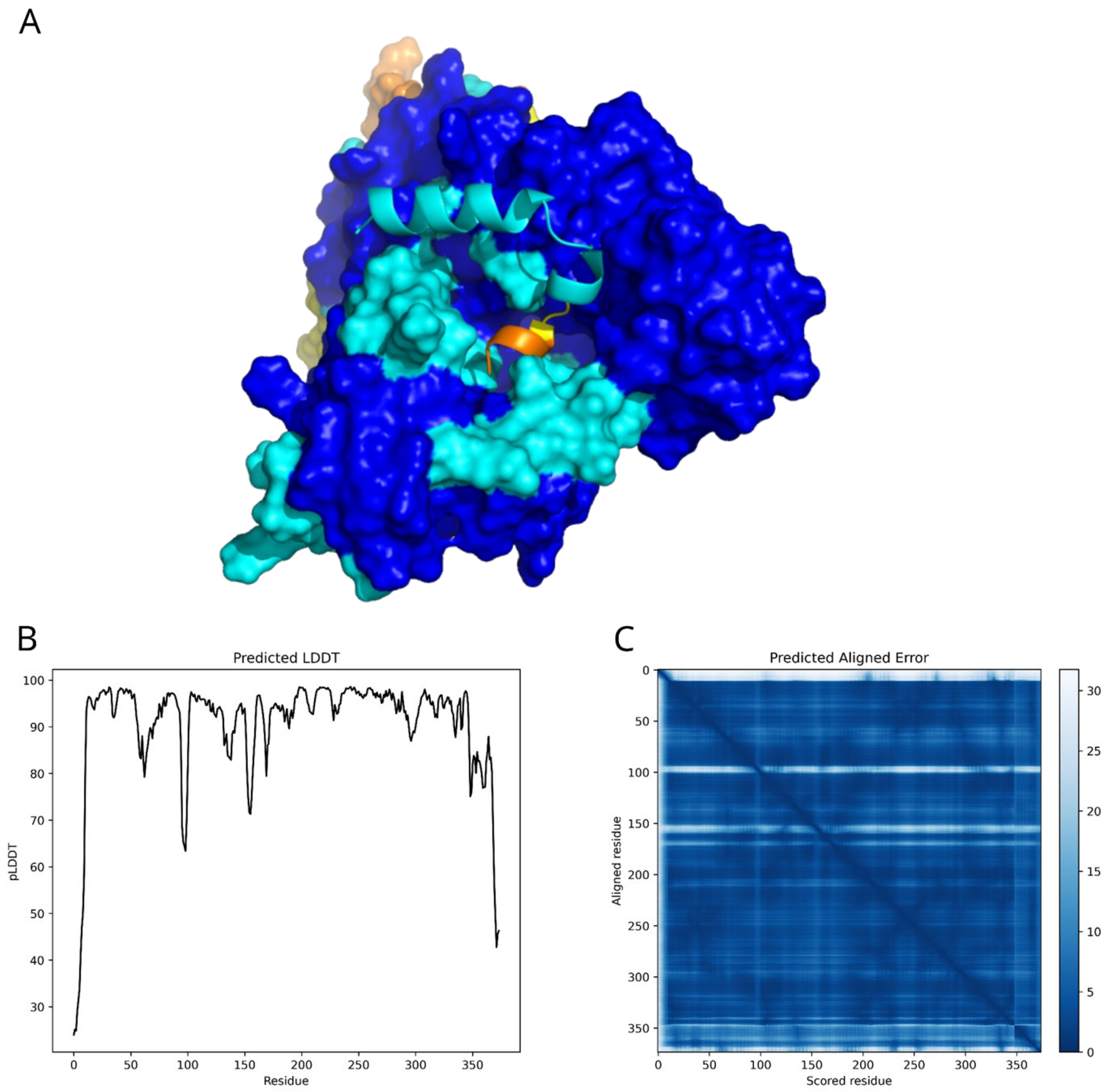

Figure S7. Metrics of the AlphaFold predicted model of precursor peptide and P450 encoded in the *trp* BGC. A) 3D structure representation: The model was predicted using AlphaFold. The structure is color-coded based on the predicted local distance difference test (pLDDT) scores. High-confidence regions with pLDDT scores above 90 are colored in blue, while regions with scores between 70 and 90 are colored in cyan. Moderate-confidence regions with scores between 50 and 70 are represented in yellow, and low-confidence regions with scores below 50 are displayed in orange. B) pLDDT Distribution: Line plot of predicted pLDDT scores against protein sequence, illustrating regions of modeling certainty and uncertainty. C) Predicted aligned error heatmap: A heatmap displaying the predicted aligned error for each pair of residues. The color intensity, ranging from dark (low error) to light blue (high error), signifies the magnitude of the aligned error, with the scale provided by a colorbar. This heatmap representation provides a comprehensive view of the alignment's reliability and potential areas where the predicted structure might deviate from a potential experimentally determined structure. In both B) and C), the P450 is represented by residues 1-449, while the precursor peptide is represented by residues 350-376.

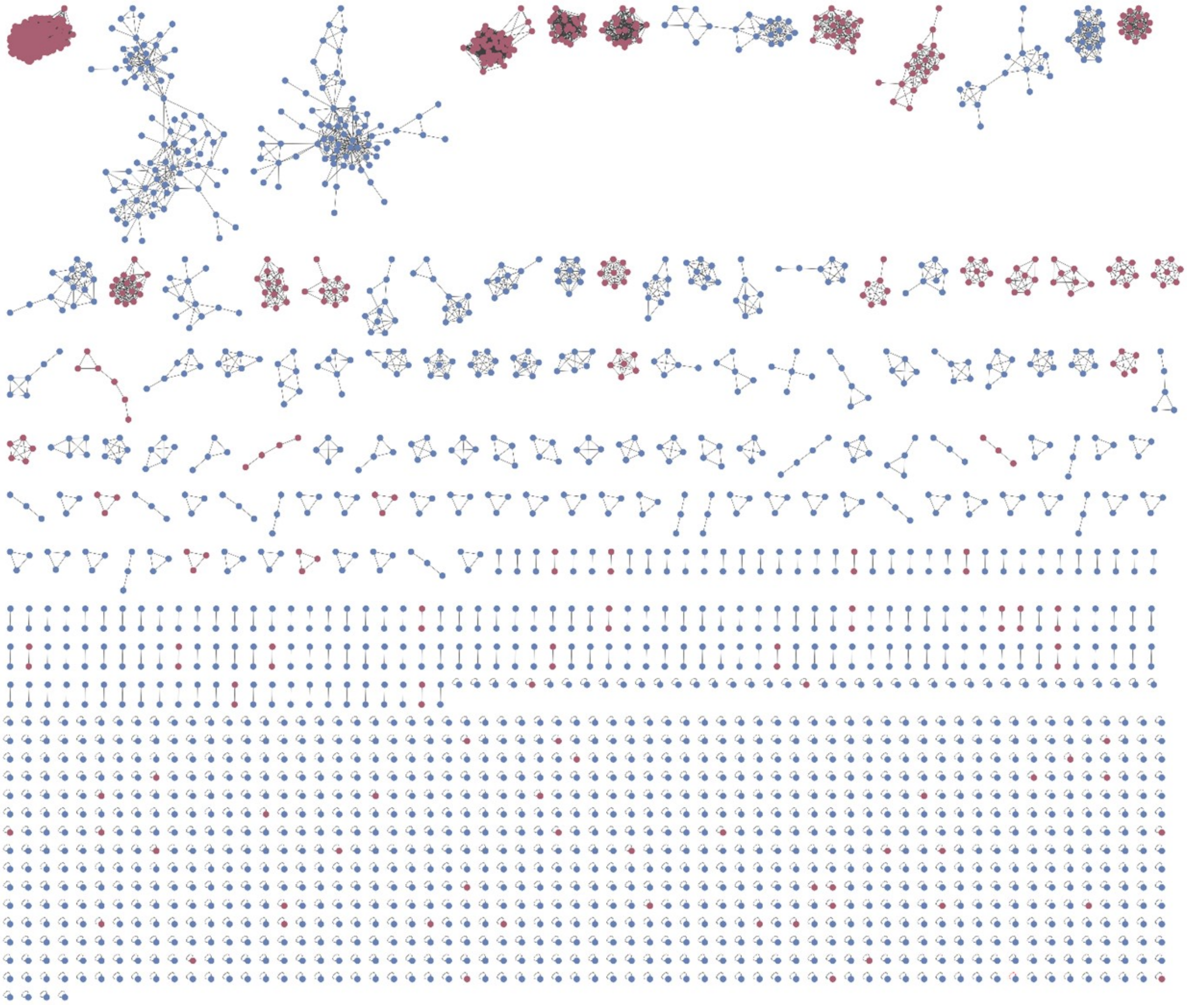

Figure S8. BiG-SCAPE analysis of all putative atropopeptide-containing gene clusters (red) and all characterized BGC deposited to the MiBIG database (blue) at threshold of 0.5. The analysis shows that atropopeptide BGCs cluster separately from all BGCs deposited to the MiBIG database.

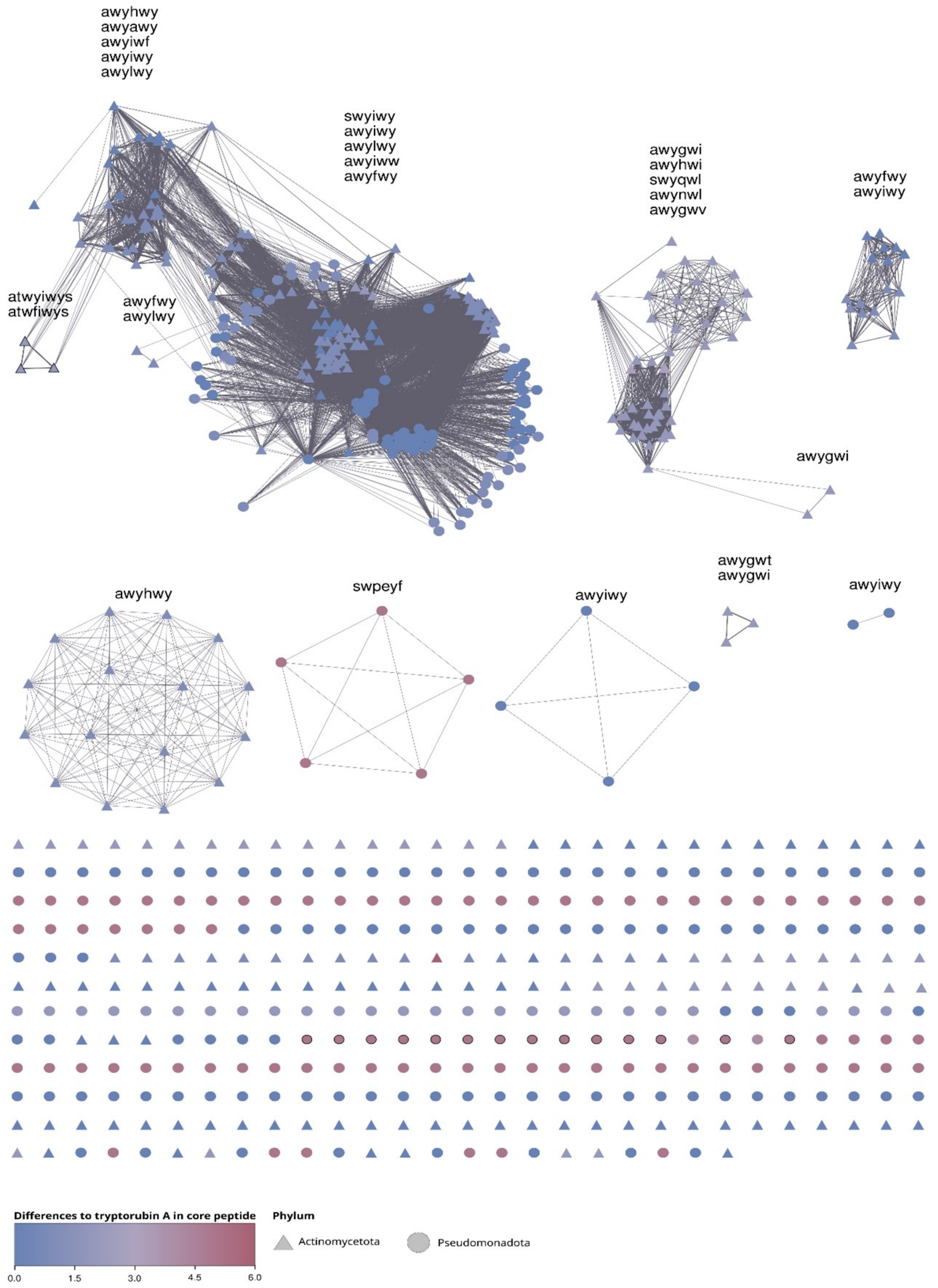

Figure S9. Sequence similarity network of all putative atropopeptide precursors and their corresponding core peptide sequences. The edge line width indicates the sequence similarity. Nodes with core peptides predicted to be longer than 6 amino acids are highlighted with a bold outline.

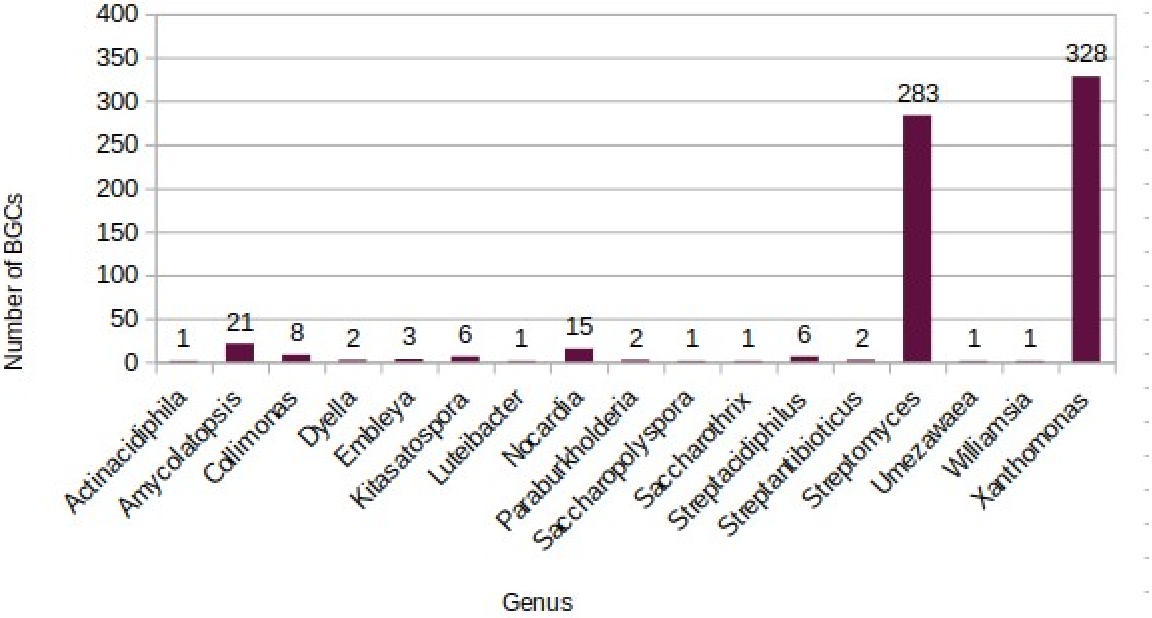

Figure S10. Phylogenetic distribution of putative atropopeptide clusters.

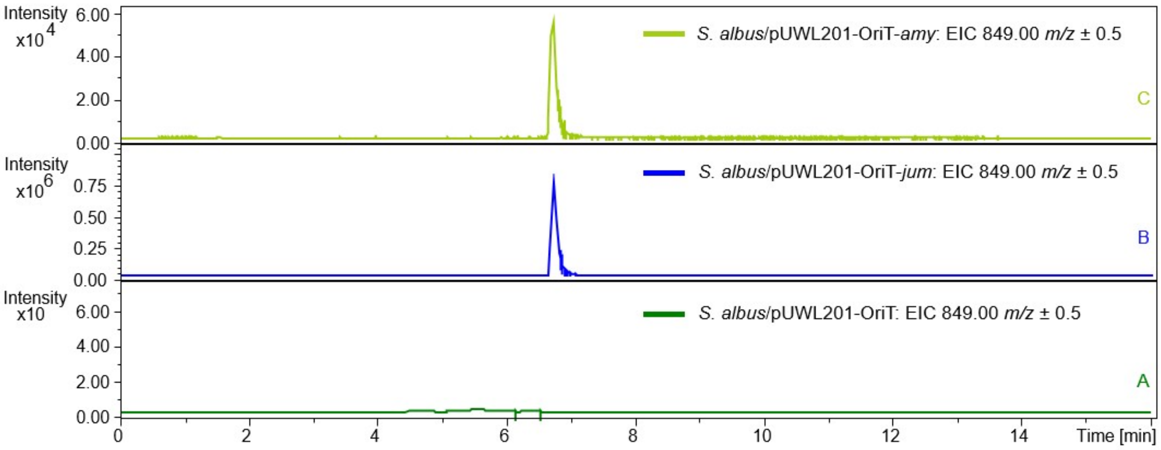

Figure S11. Extracted ion chromatograms of amyxirubin B and jumorubin (**2**). (A) *S. albus* harboring the empty pUWL201-oriT. (B) jumorubin produced by heterologous expression of the jumorubin BGC in *S. albus* J1074. (C) amyxirubin B produced by heterologous expression of the amyxirubin BGC in *S. albus* J1074.

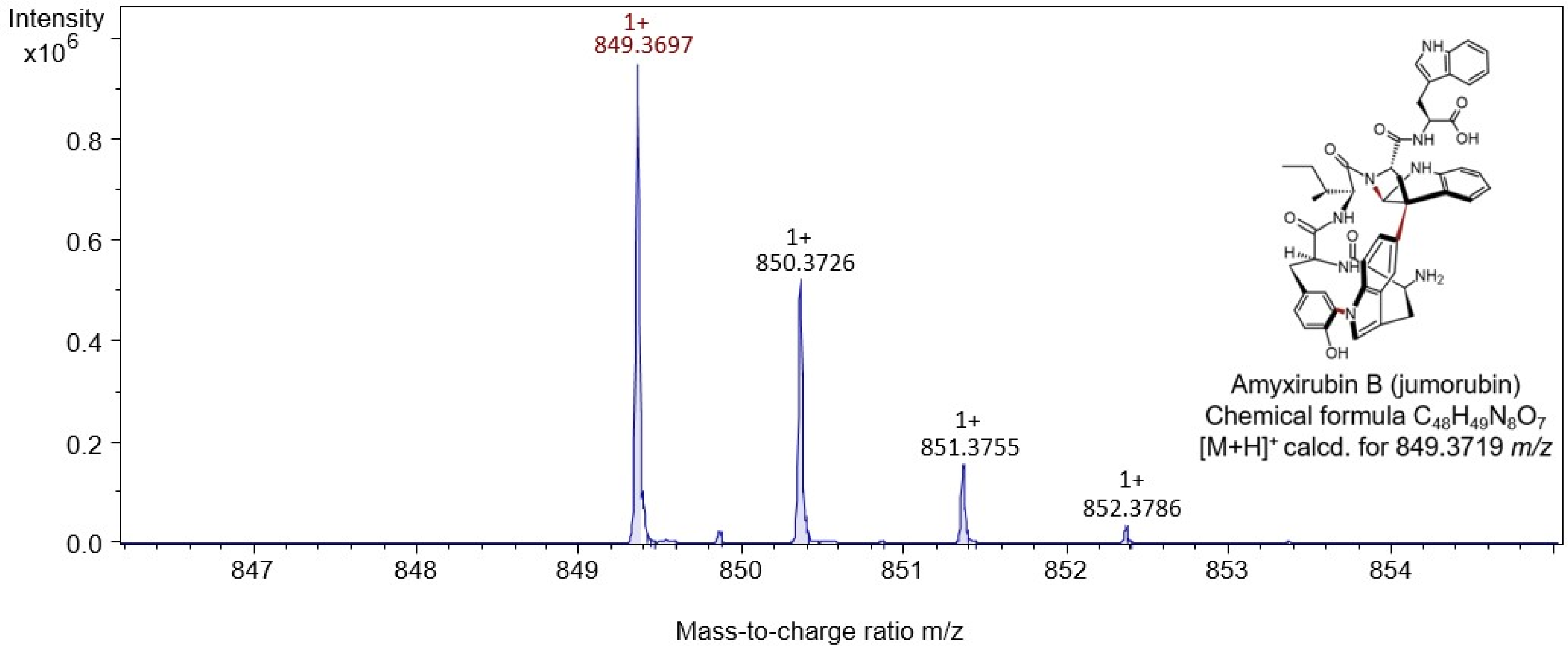

Figure S12. HPLC-ESI-QTOF-HRMS analysis of jumorubin (**2**).

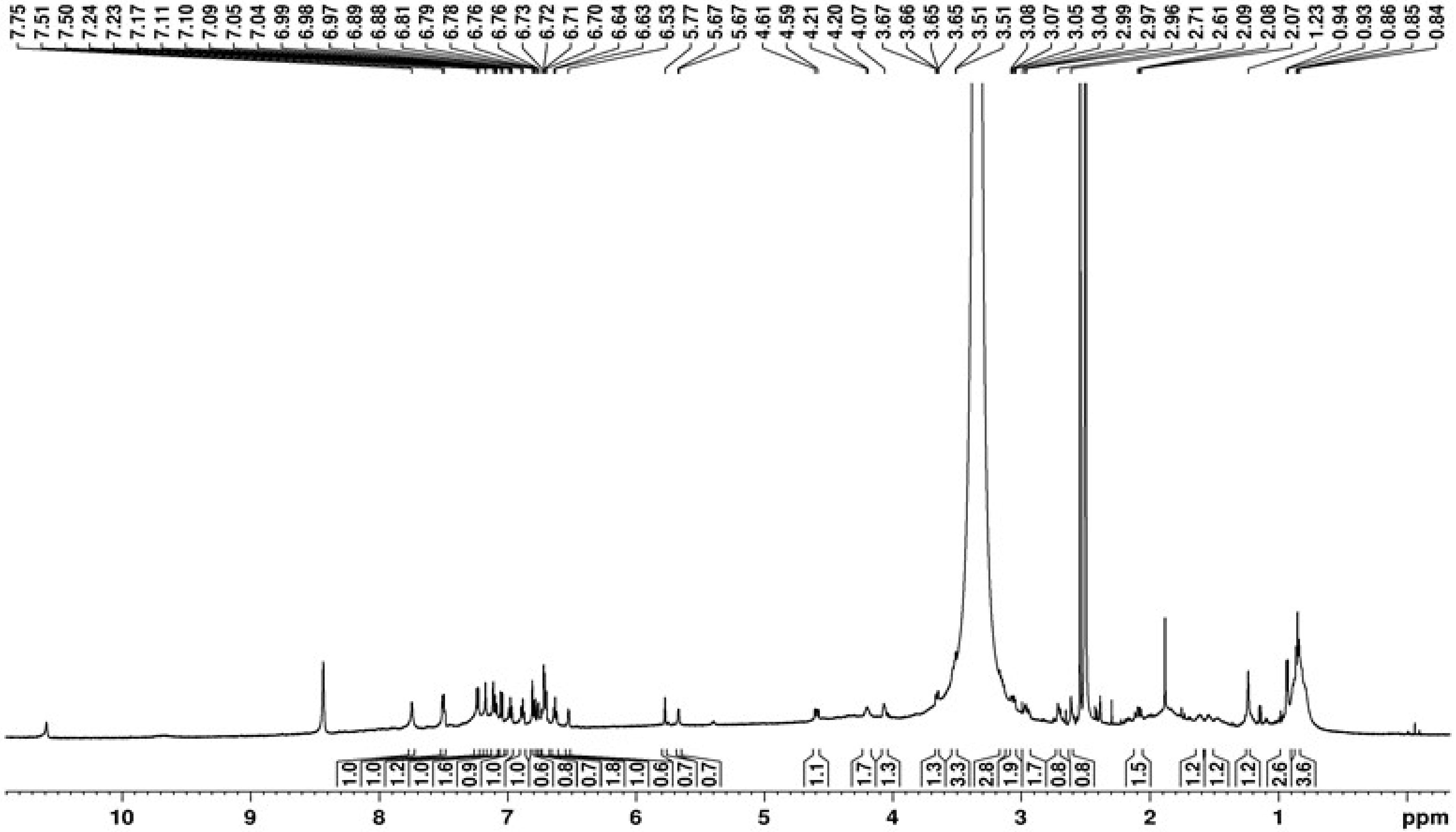

Figure S13. ^{1}H NMR spectrum (600 MHz) of jumorubin (**2**) in DMSO-d_6.

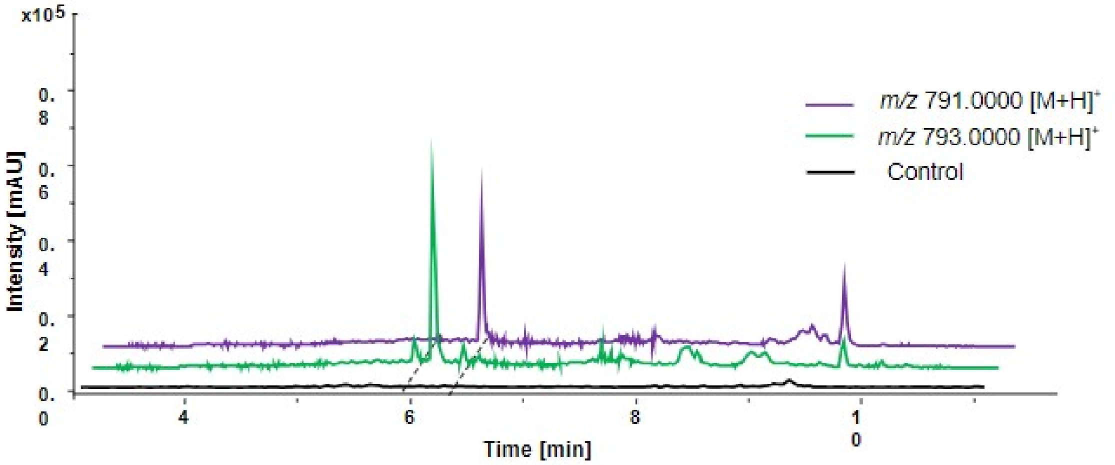

Figure S14. Extracted ion chromatogram (EIC) of varsorubin B1 (**3**) and varsorubin B2 (**4**) from the control *S. albus* harboring the empty pUWL201-oriT (black chromatogram) and *S. albus* harboring pUWL201-oriT-Sva (green and purple chromatogram)

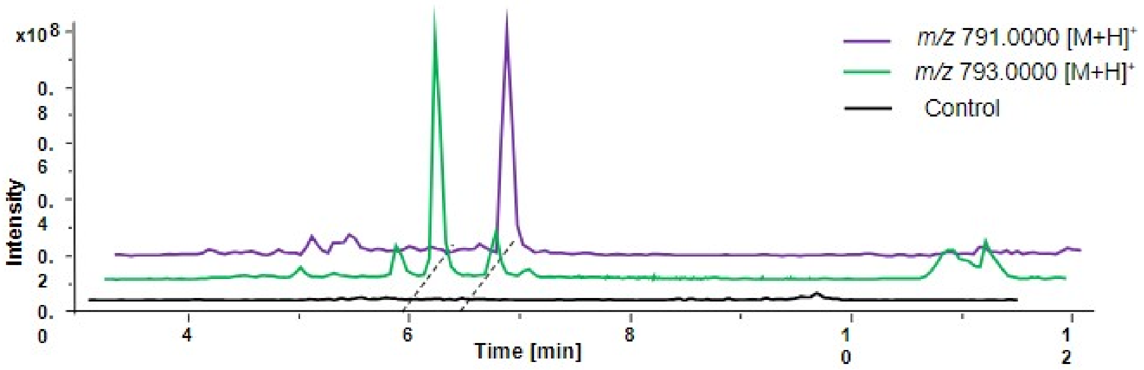

Figure S15. Extracted ion chromatogram (EIC) of varsorubin B1 (**3**) and varsorubin B2 (**4**) from the control *S. albus* harboring pUWL201-oriT (black chromatogram) and *S. albus* harboring pUWL201-oriT-SvaR (green and purple chromatogram)

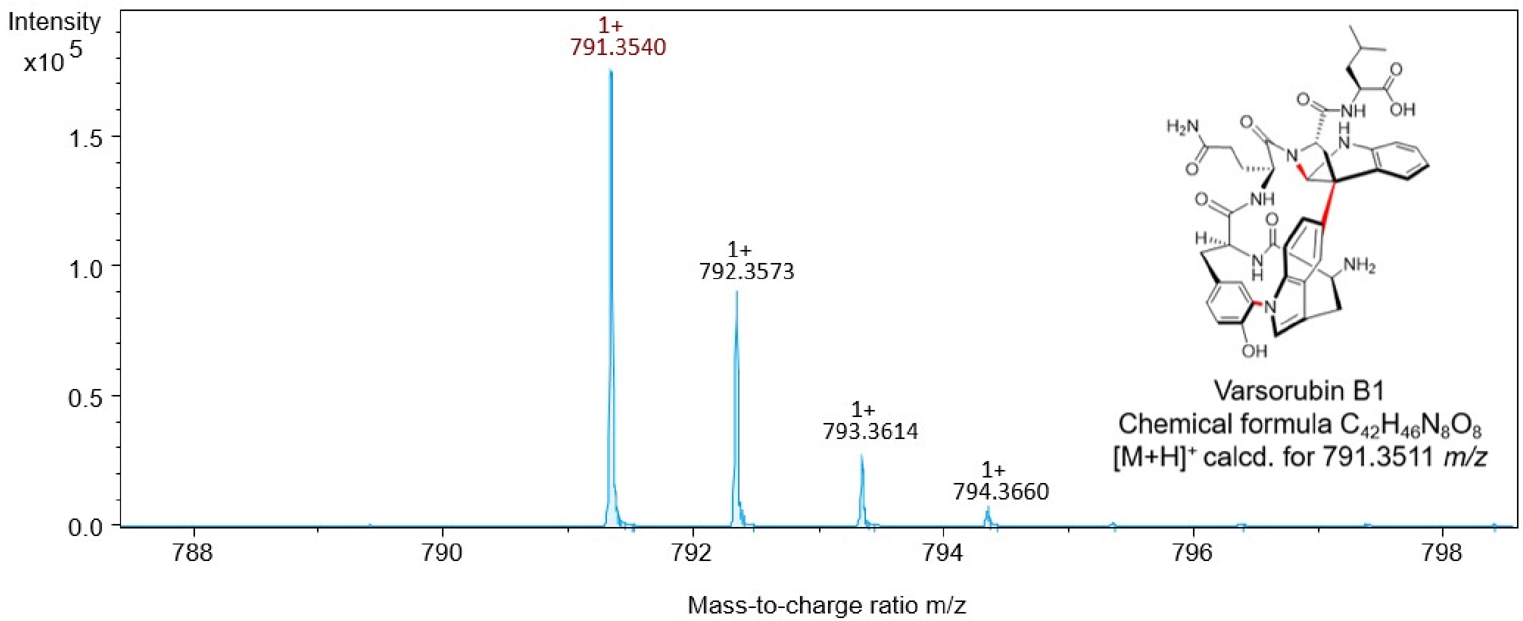

Figure S16. HPLC-ESI-QTOF-HRMS analysis of varsorubin B1 (**3**).

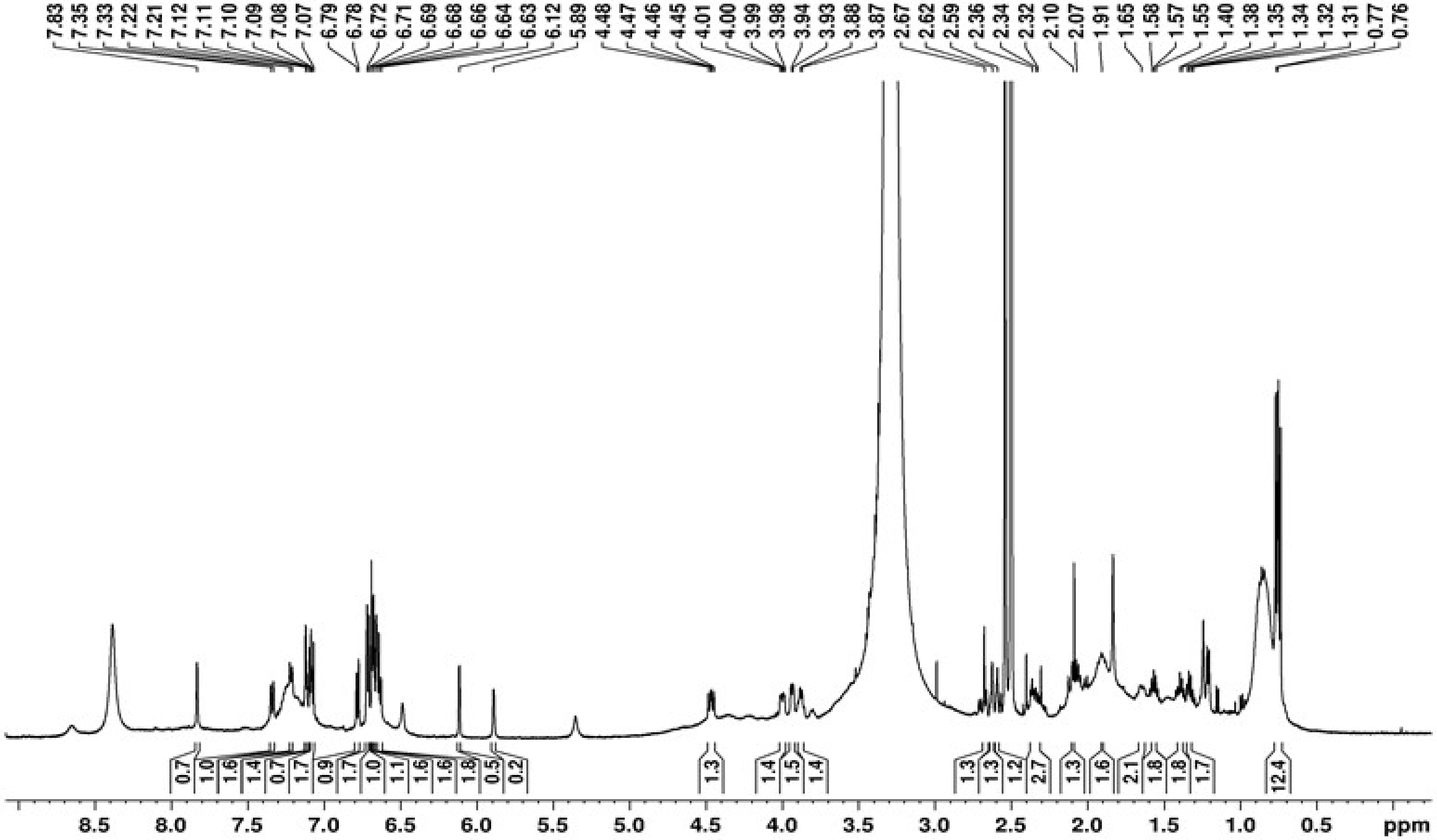

Figure S17. ^1H NMR spectrum (600 MHz) of varsorubin B1 (**3**) in DMSO-d_6.

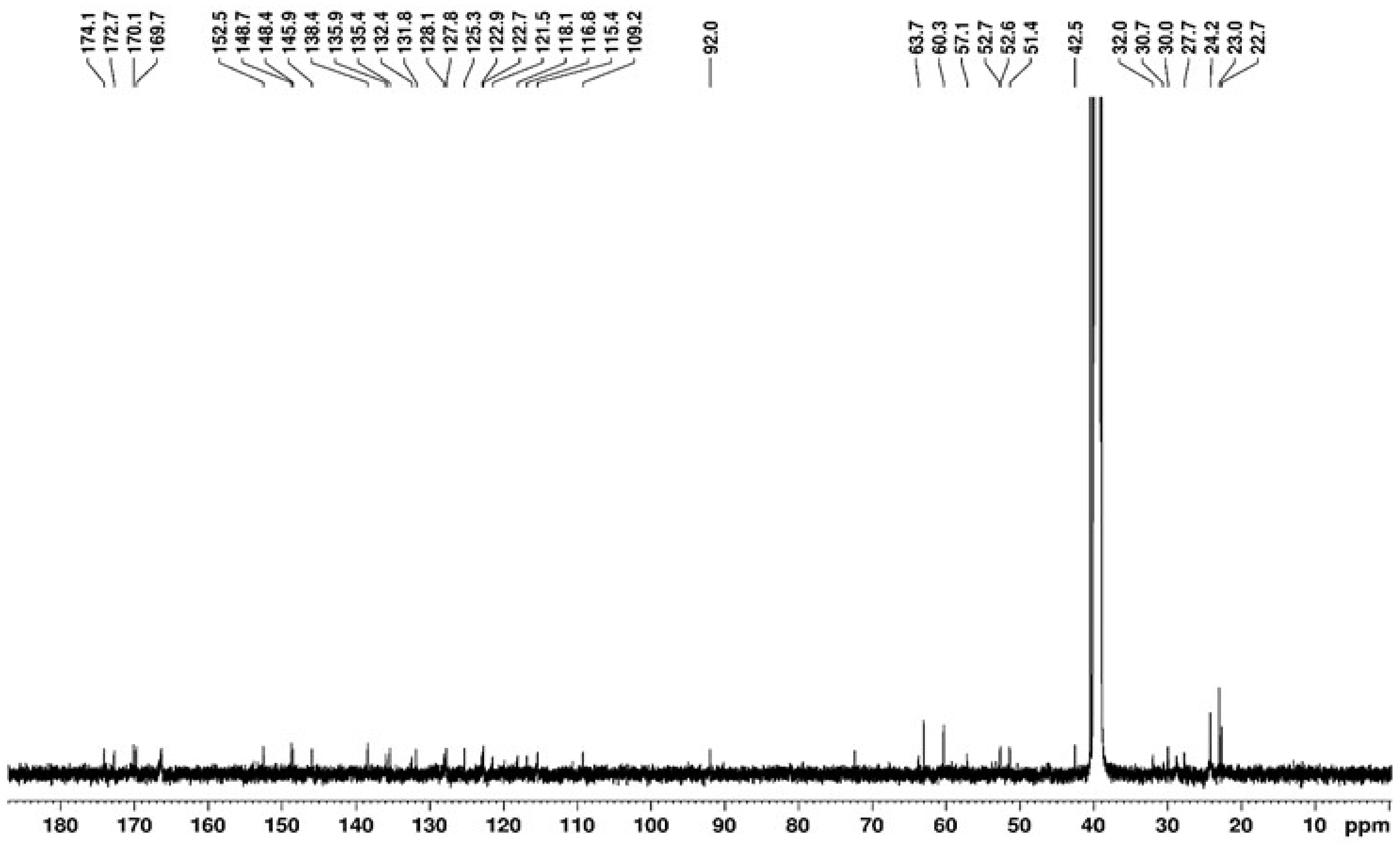

Figure S18. ^{13}C NMR spectrum (150 MHz) of varsorubin B1 (**3**) in DMSO-d_6.

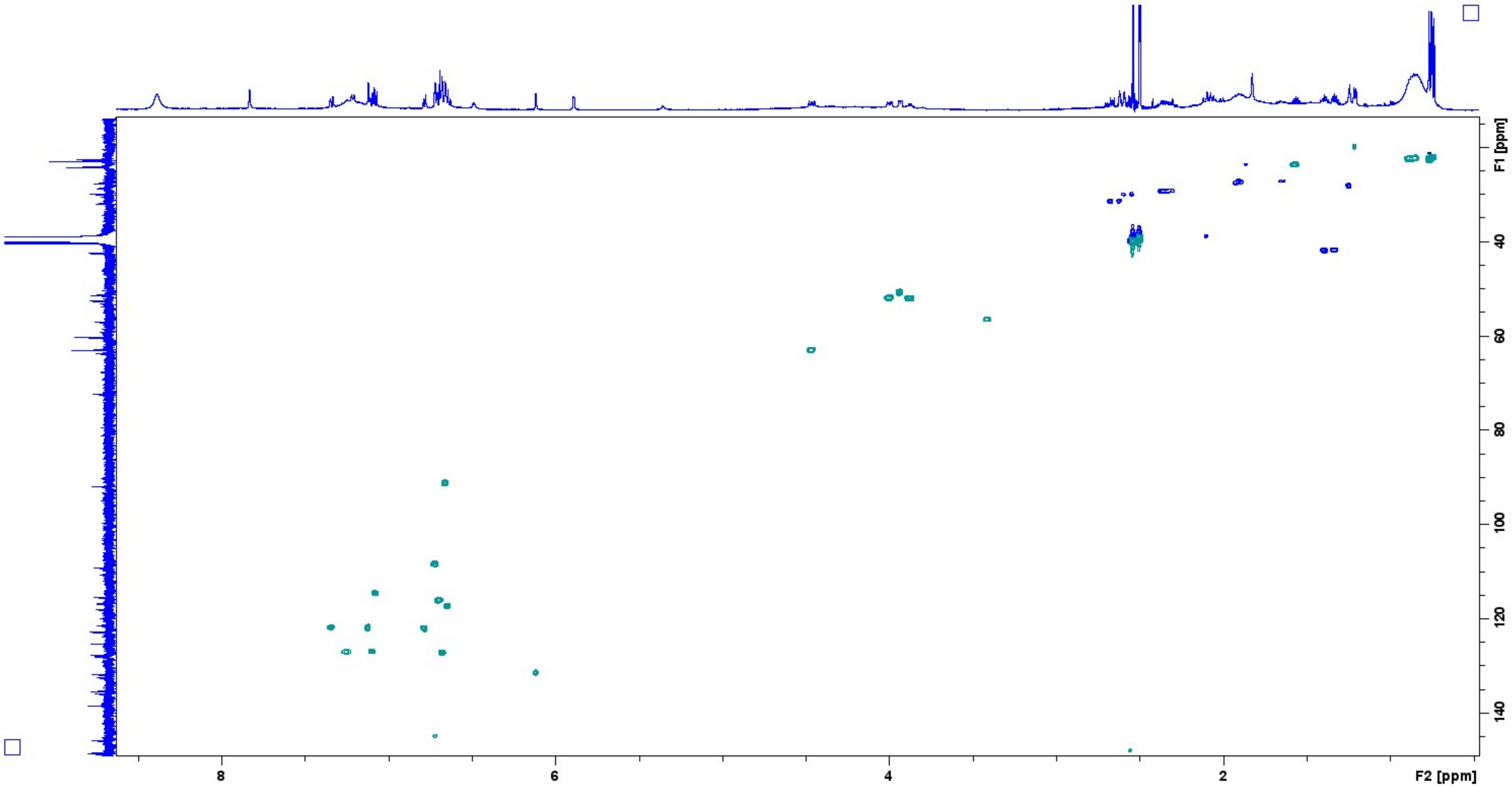

Figure S19. HSQC spectrum (600 MHz) of varsorubin B1 (**3**) in DMSO-d_6.

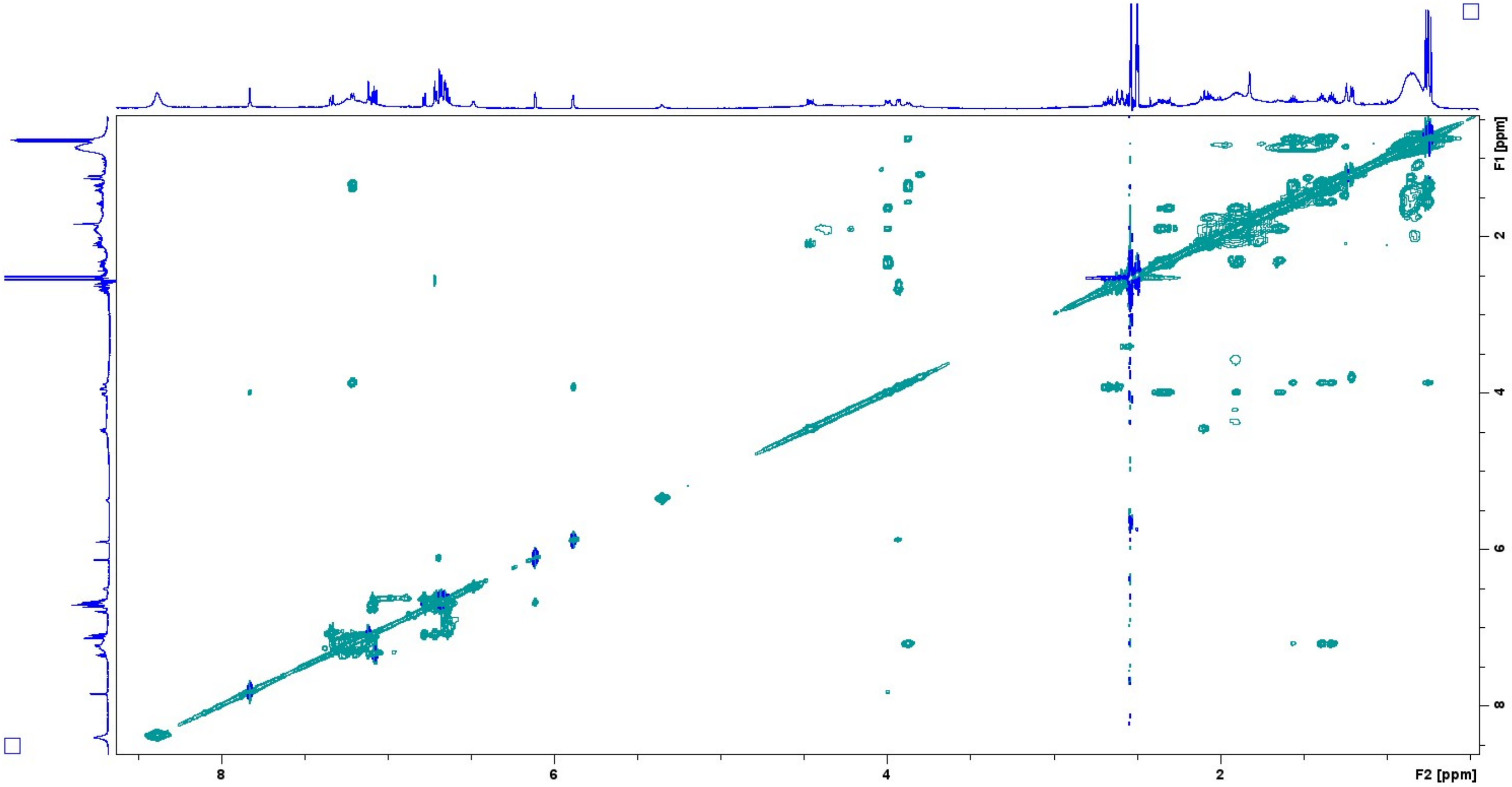

Figure S20. COSY spectrum (600 MHz) of varsorubin B1 (**3**) in DMSO-d_6.

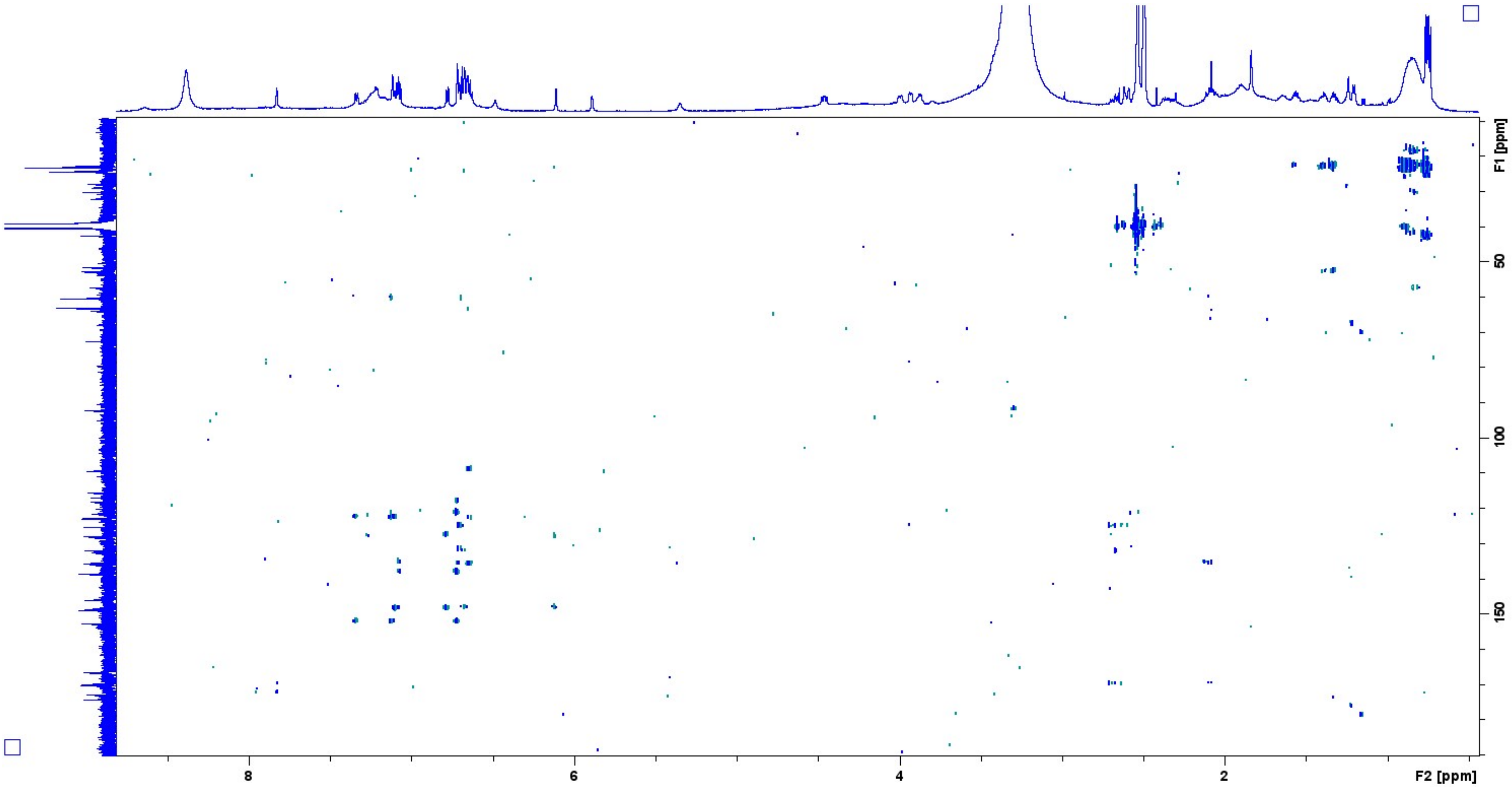

Figure S21. HMBC spectrum (600 MHz) of varsorubin B1 (**3**) in DMSO-d_6.

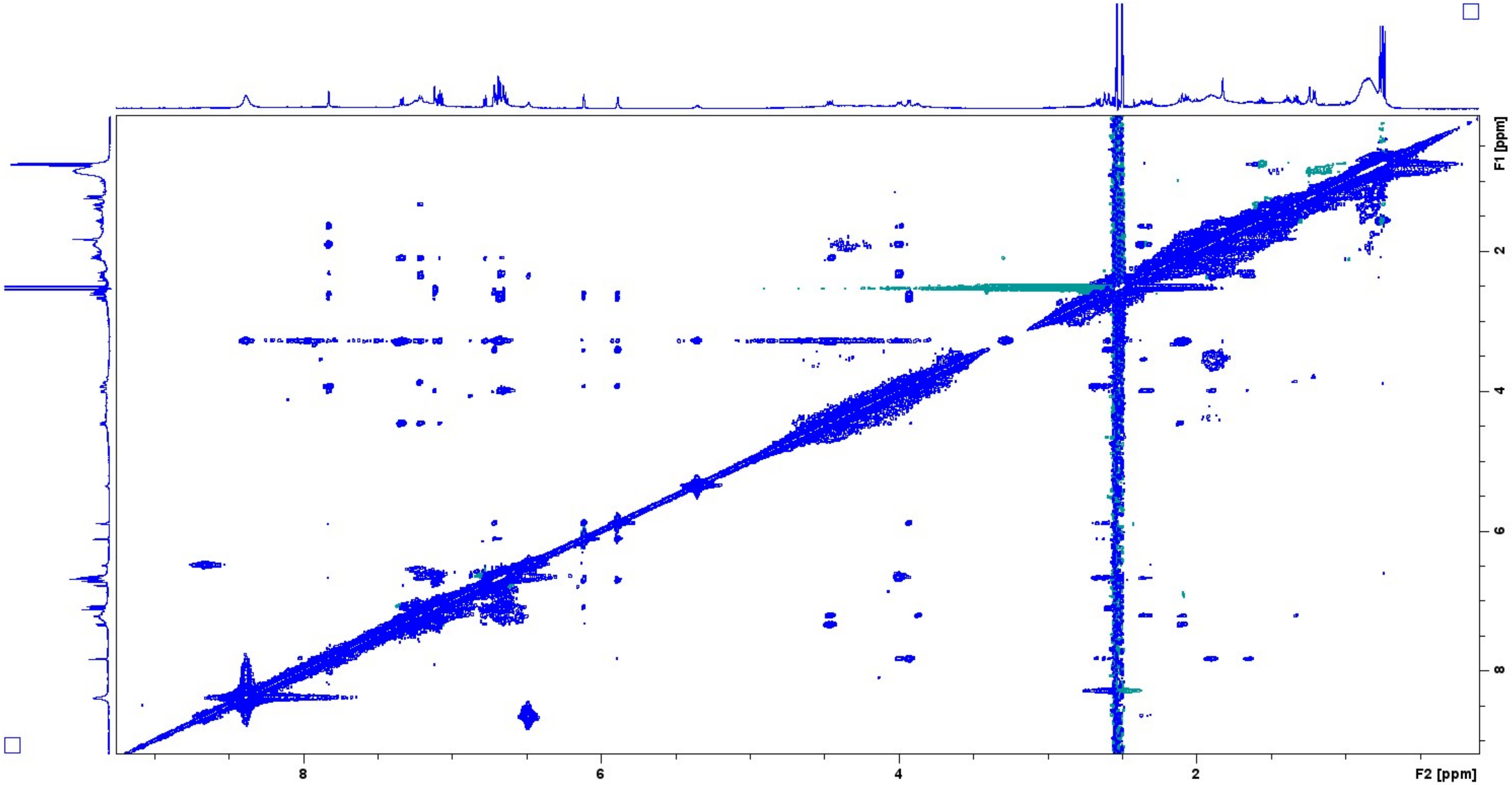

Figure S22. NOESY spectrum (600 MHz) of varsorubin B1 (**3**) in DMSO-d_6.

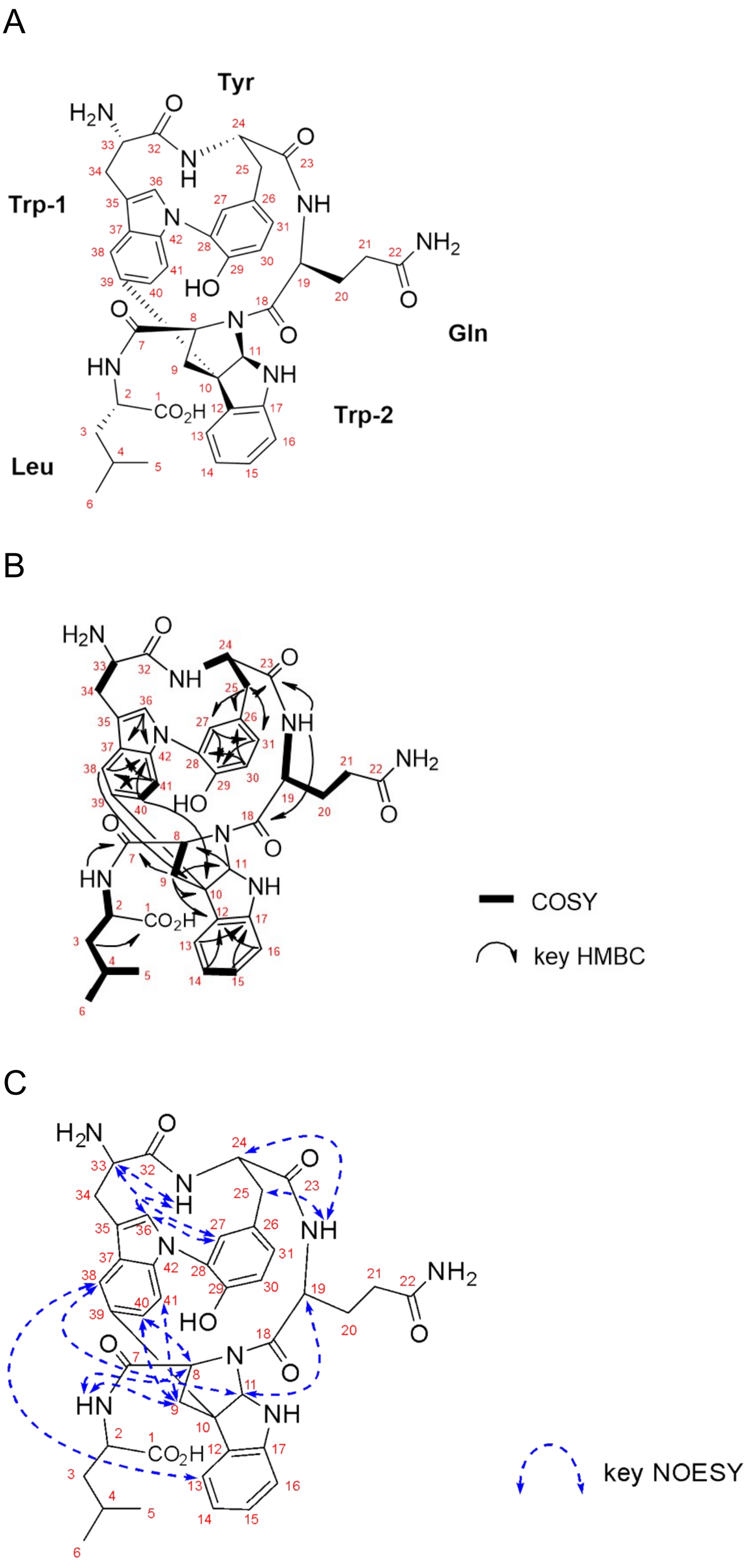

Figure S23. Structure elucidation of varsorubin B1 (**3**). (A) Structure of varsorubin B1 (**3**) and atom numbering used throughout this study. (B) COSY and key HMBC correlations in DMSO-d_6. (C) Key NOESY correlations of varsorubin B1 (**3**) in DMSO-d_6.

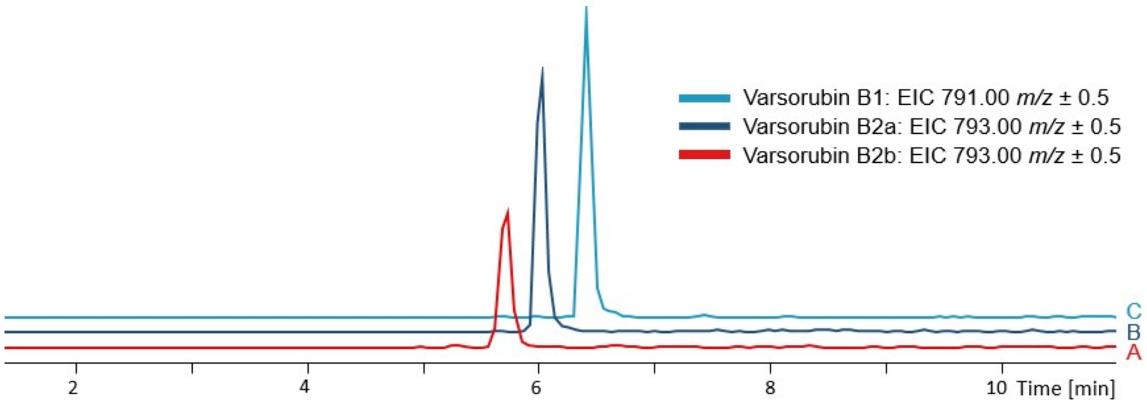

Figure S24. Extracted ion chromatograms of (A) varsorubin B2b (**4b**), (B) varsorubin B2a (**4a**) and (C) varsorubin B1 (**3**) from *S. albus* harboring pUWL201-oriT-SvarCore1R.

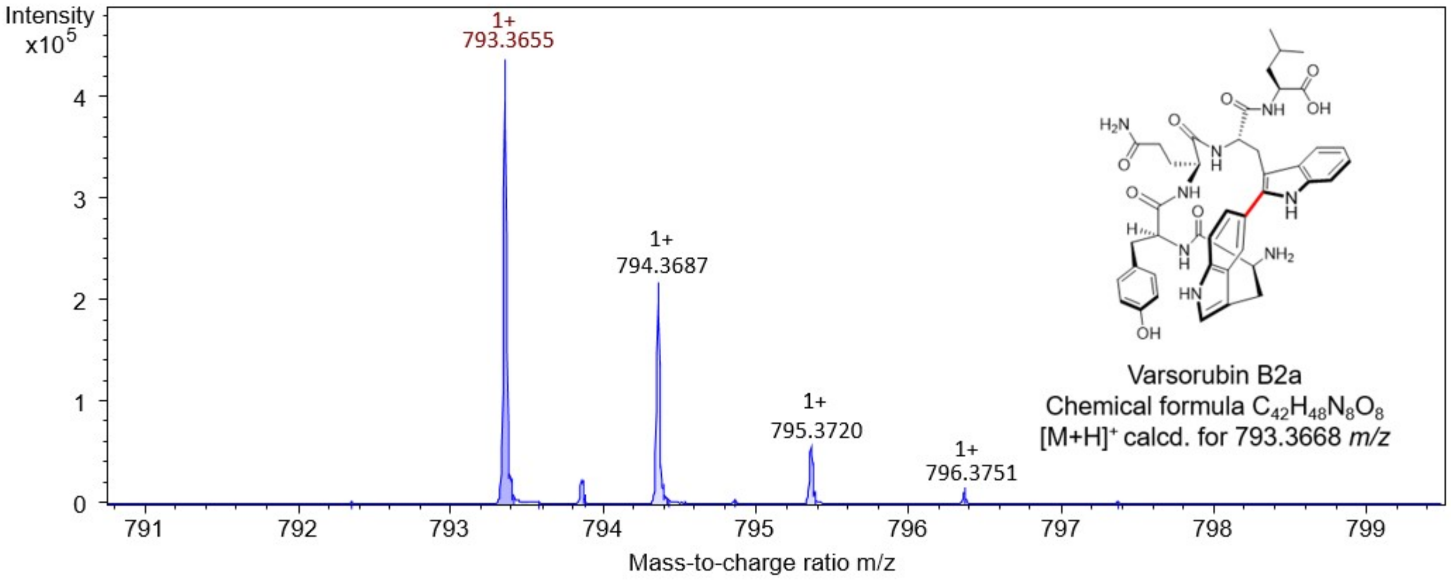

Figure S25. HPLC-ESI-QTOF-HRMS analysis of varsorubinvarsitalin B2a (**4a**).

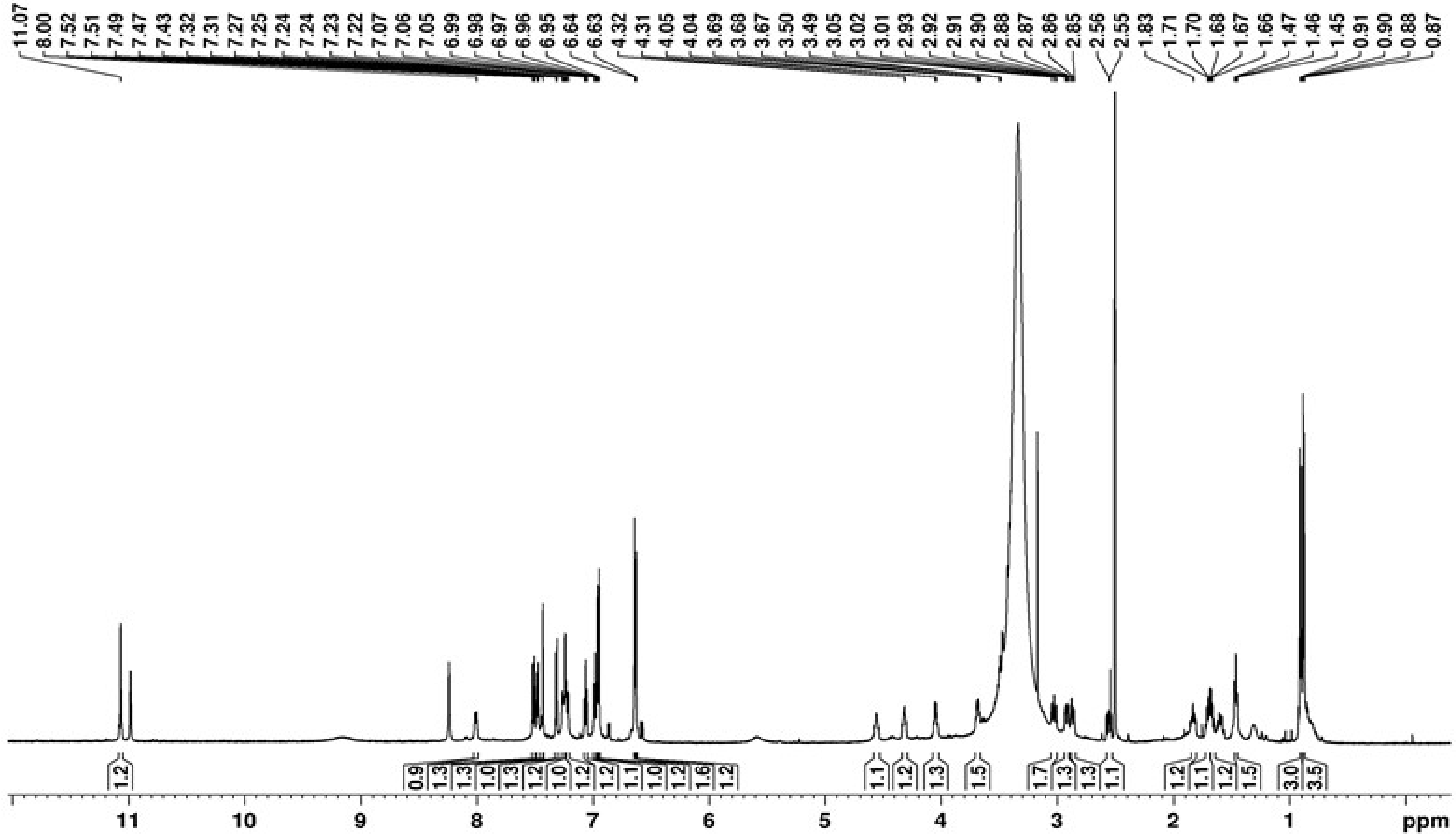

Figure S26. ^{1}H NMR spectrum (600 MHz) of varsorubin B2a (**4a**) in DMSO-d_6.

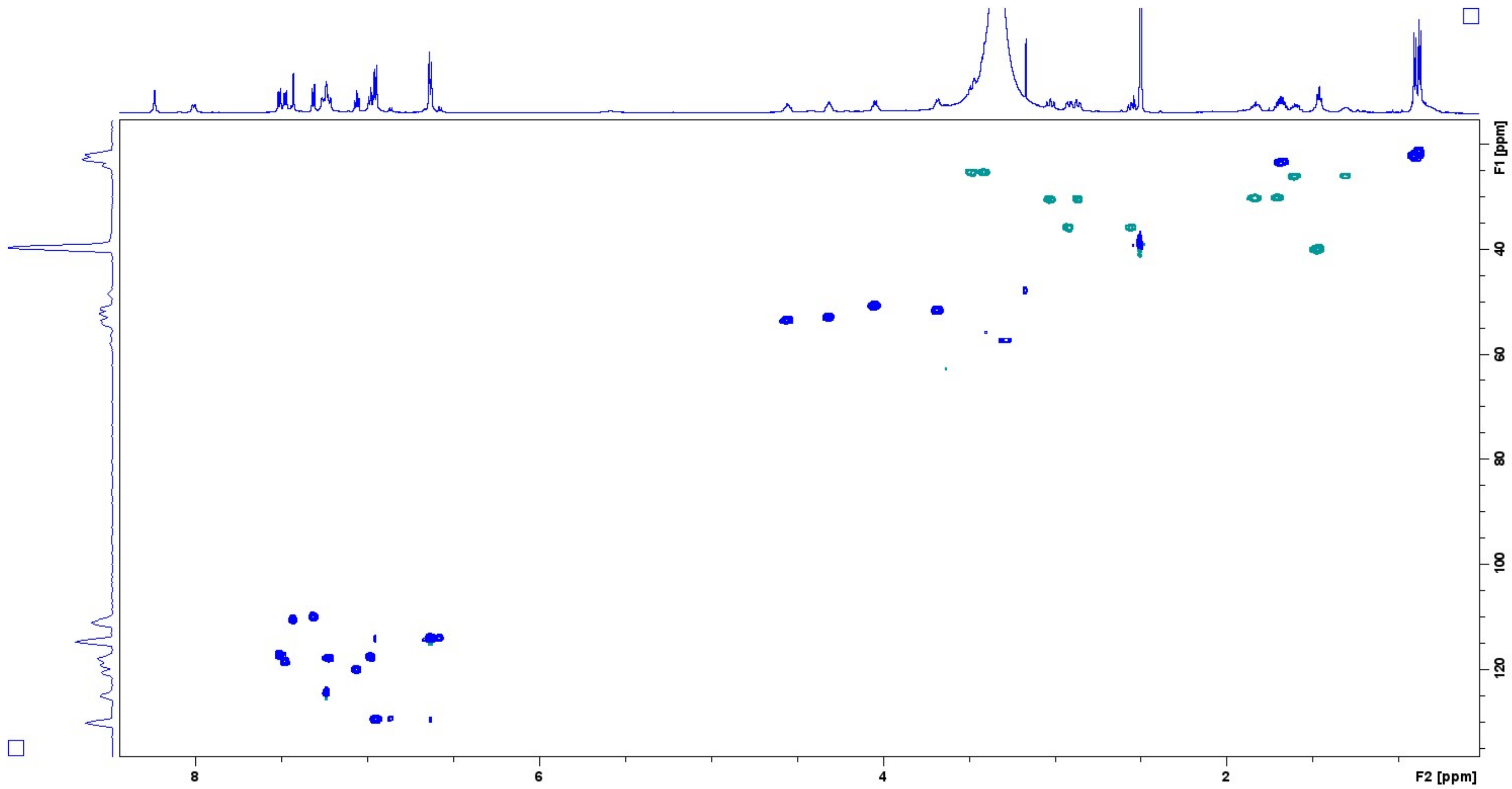

Figure S27. HSQC spectrum (600 MHz) of varsorubin B2a (**4a**) in DMSO-d_6.

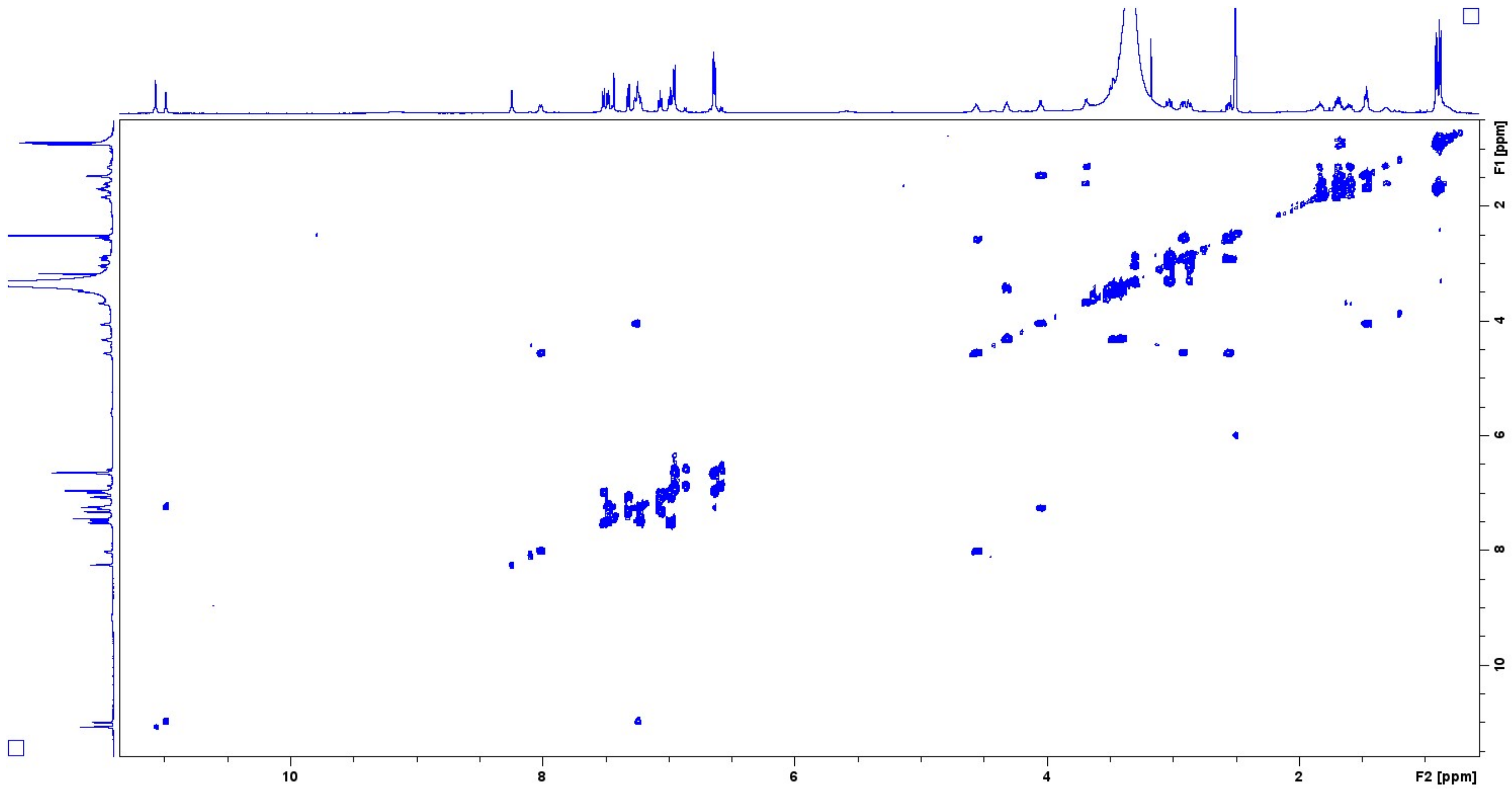

Figure S28. COSY spectrum (600 MHz) of varsorubin B2a (**4a**) in DMSO-d_6.

A

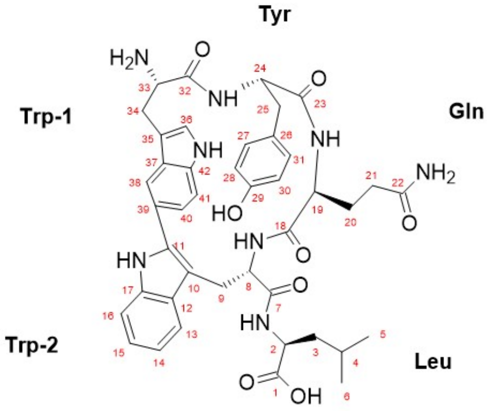

B

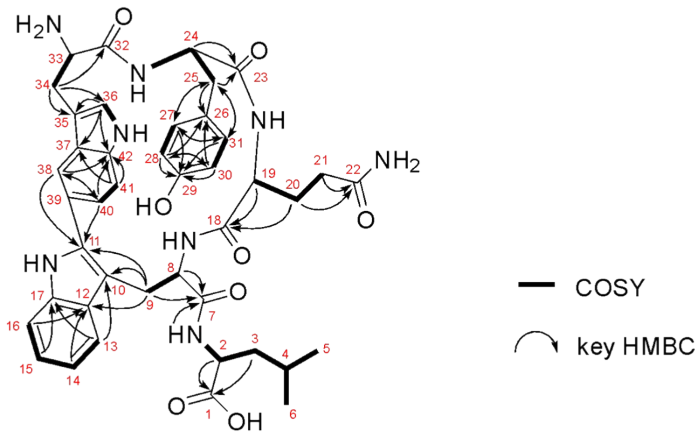

Figure S29. Structure elucidation of varsorubin B2a (**4a**). (A) Structure of varsorubin B2a (**4a**) and atom numbering used throughout this study. (B) COSY and key HMBC correlations in DMSO-d_6.

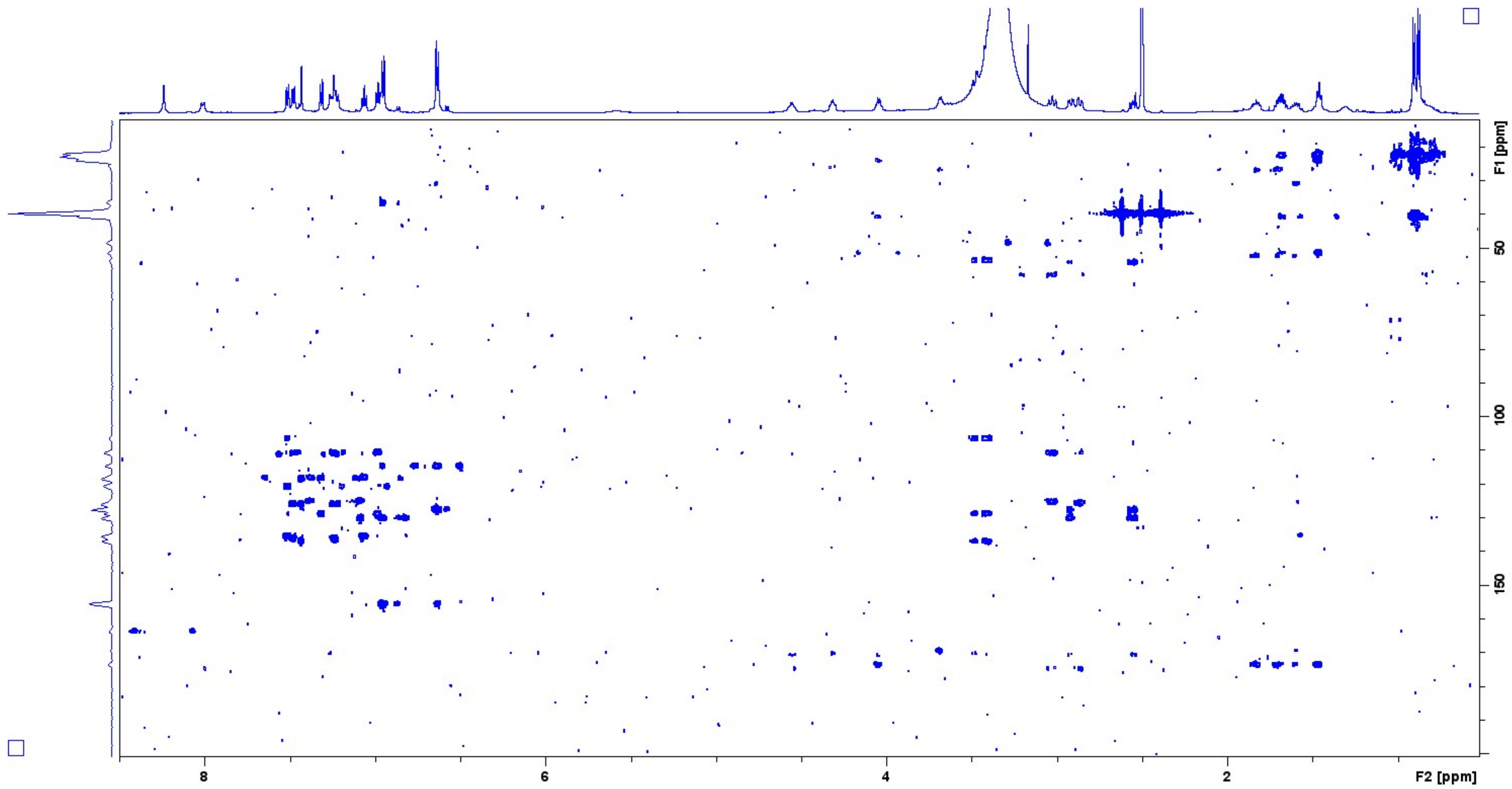

Figure S30. HMBC spectrum (600 MHz) of varsorubin B2a (**4a**) in DMSO-d_6.

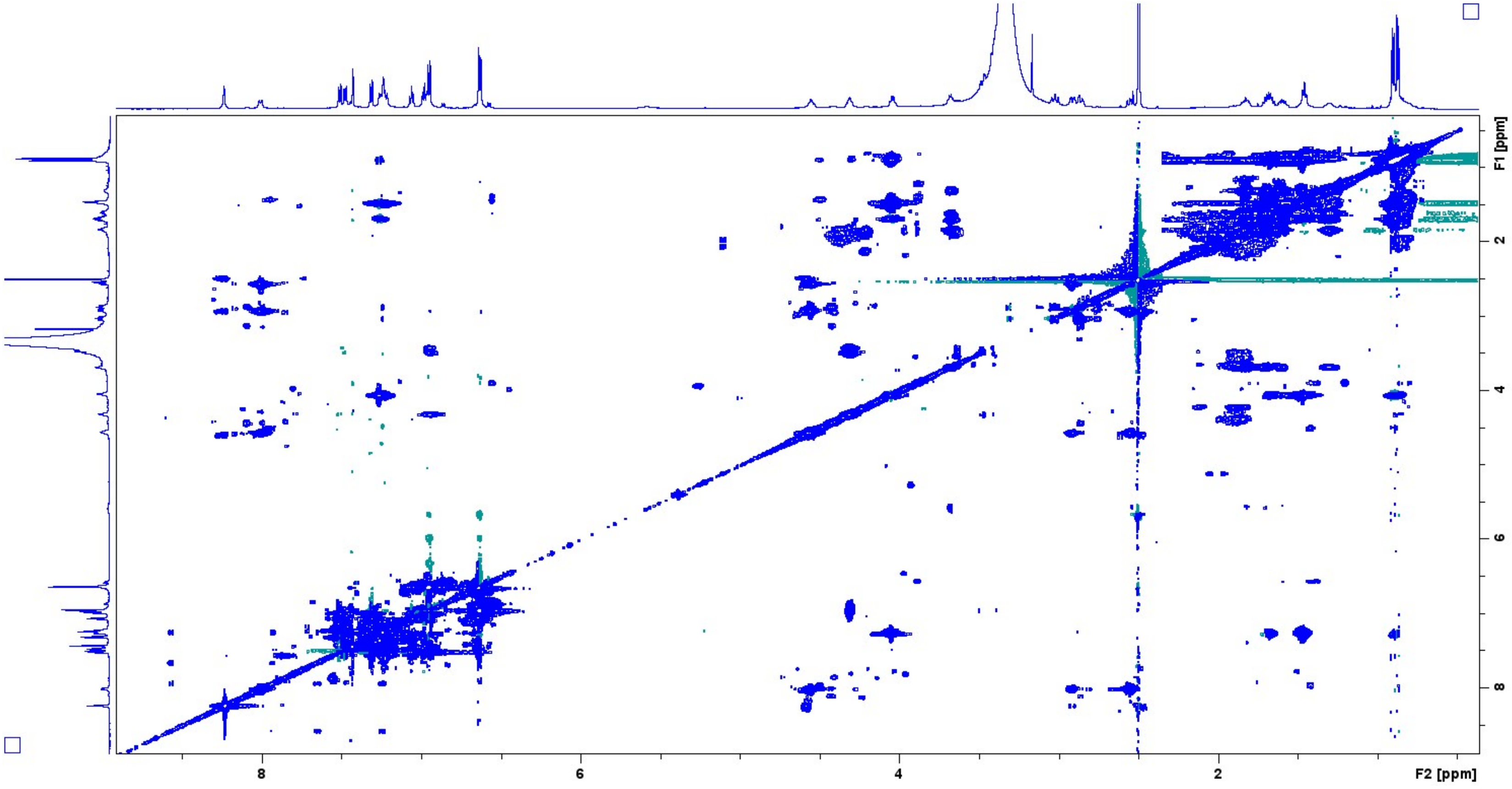

Figure S31. TOCSY spectrum (600 MHz) of varsorubin B2a (**4a**) in DMSO-d_6.

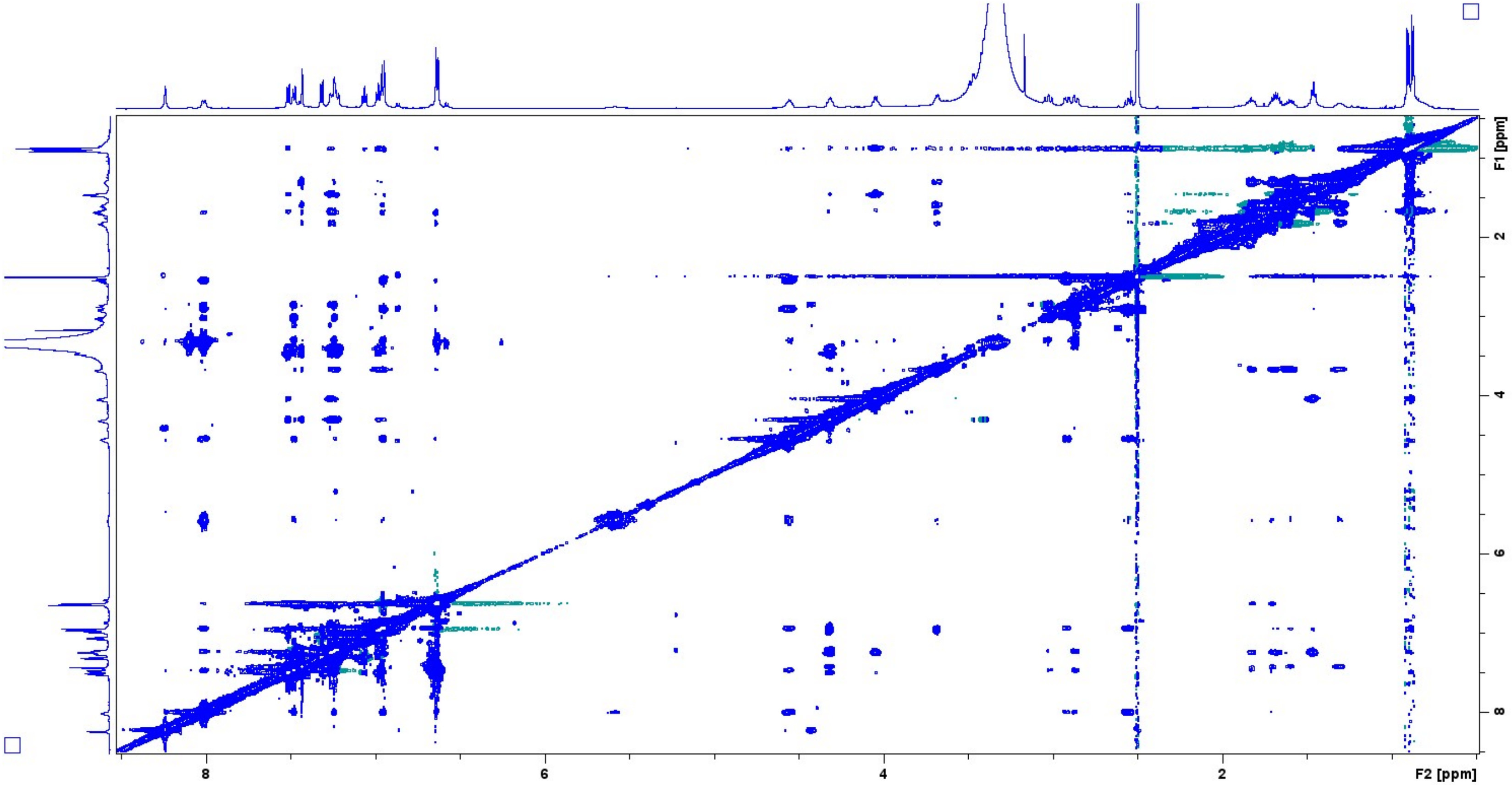

Figure S32. NOESY spectrum (600 MHz) of varsorubin B2a (**4a**) in DMSO-d_6.

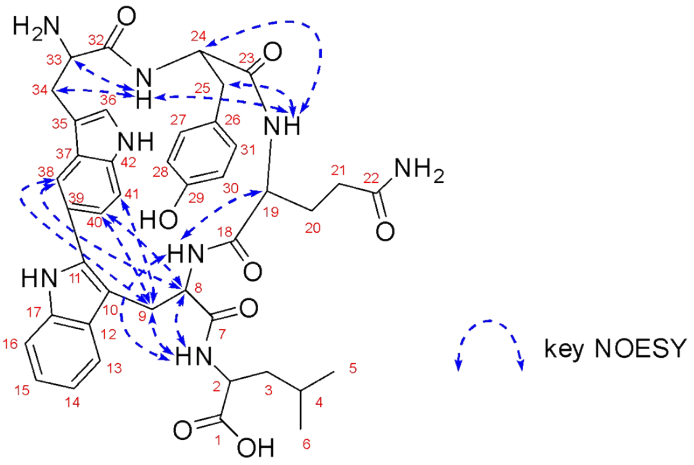

Figure S33. Key NOESY correlations of varsorubin B2a (**4a**) in DMSO-d_6.

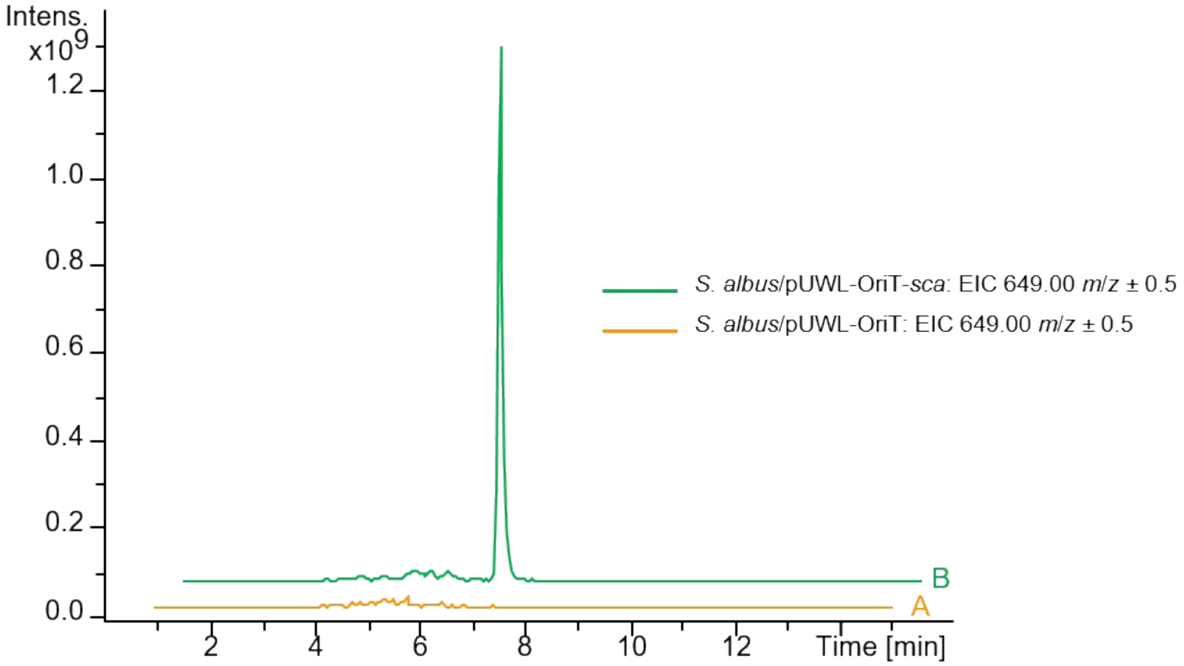

Figure S34. Extracted ion chromatogram of scabrirubin (**5**) from *S. albus* harboring pUWL201-oriT (A) and *S.albus* harboring pUWL201-OriT-*scaerm* (B).

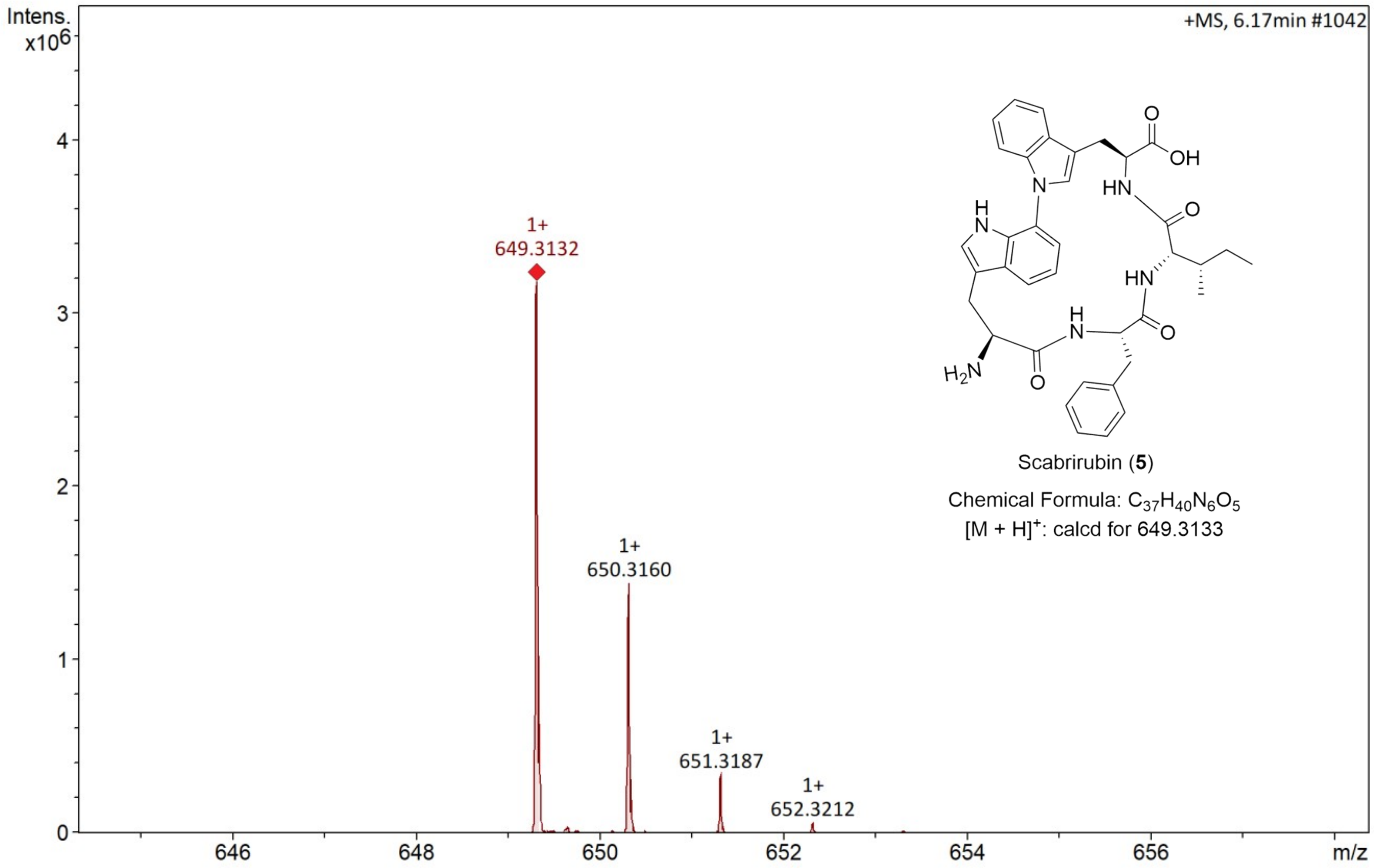

Figure S35. HPLC-ESI-QTOF-HRMS analysis of scabrirubin (**5**).

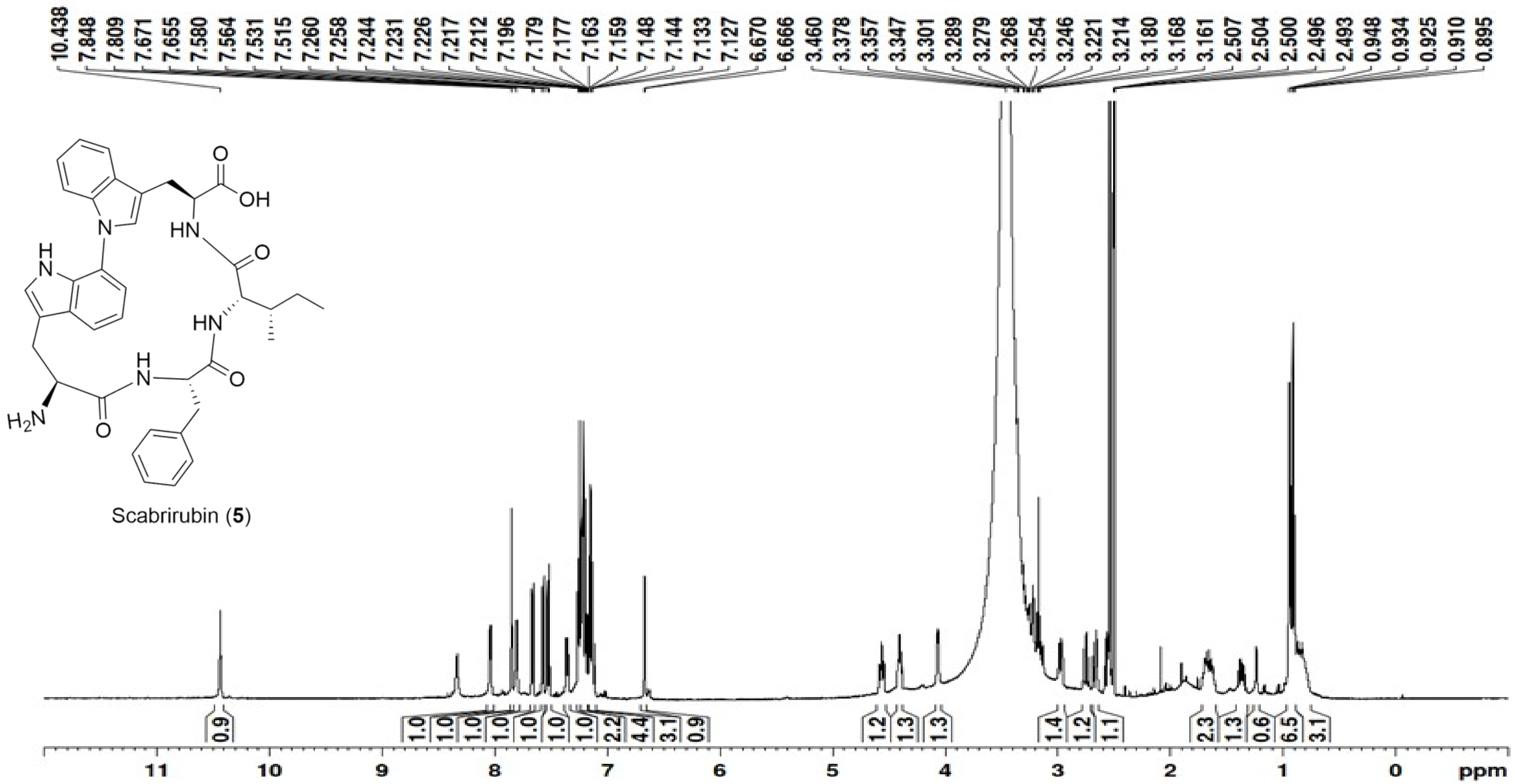

Figure S36. ^{1}H-NMR spectrum (500 MHz) of scabrirubin (**5**) in DMSO-d_6.

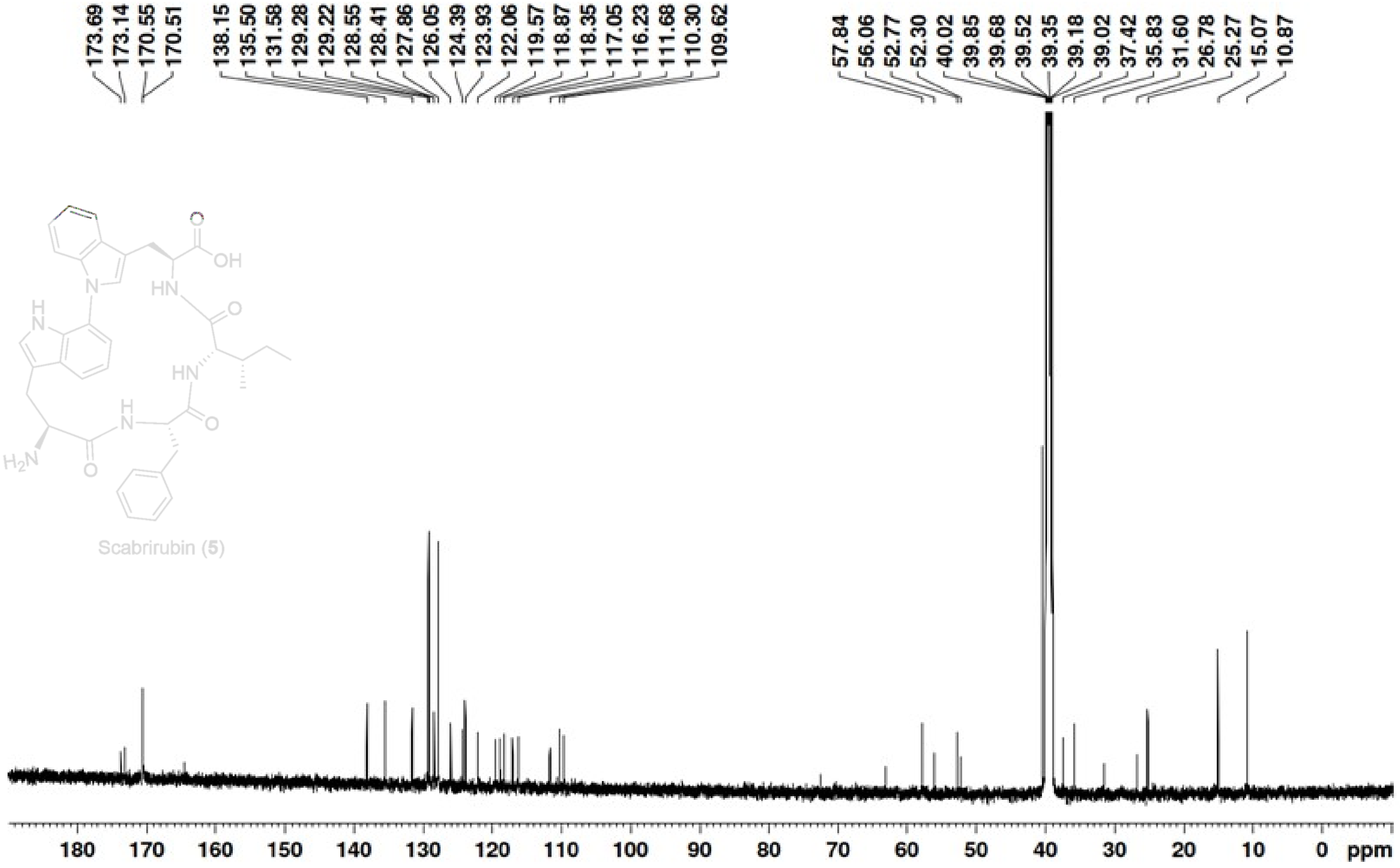

Figure S37. ^{13}C-NMR spectrum (125 MHz) of scabrirubin (**5**) in DMSO-d_6.

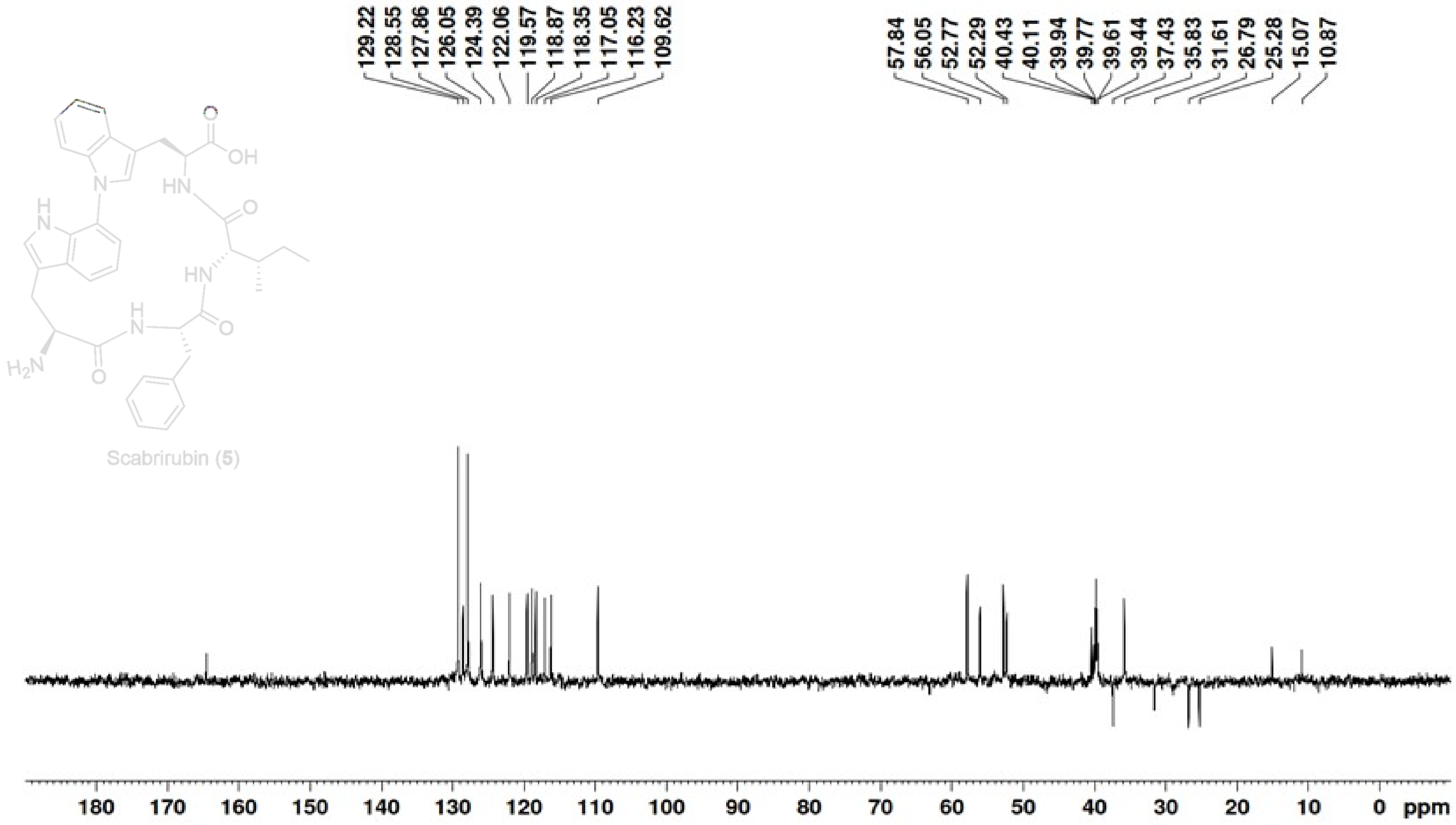

Figure S38. DEPT135 spectrum (125 MHz) of scabrirubin (**5**) in DMSO-d_6.

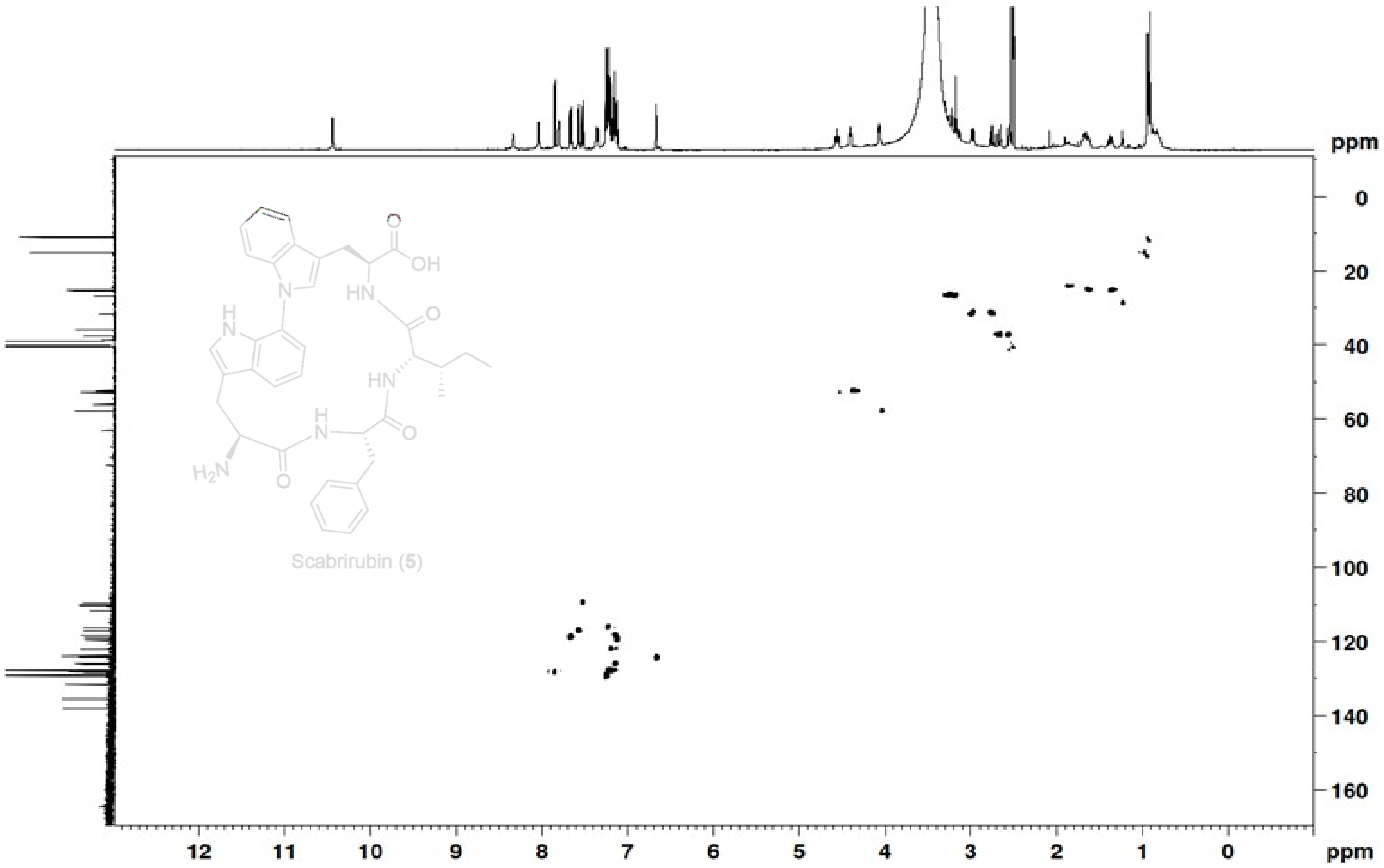

Figure S39. HSQC spectrum (500 MHz) of scabrirubin (**5**) in DMSO-d_6.

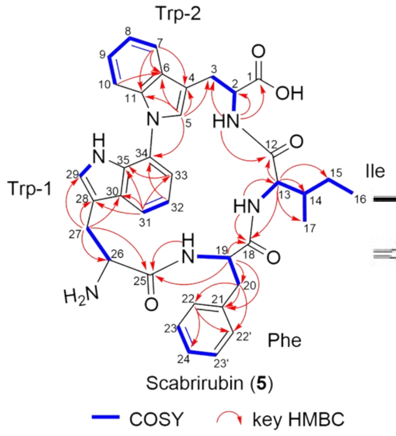

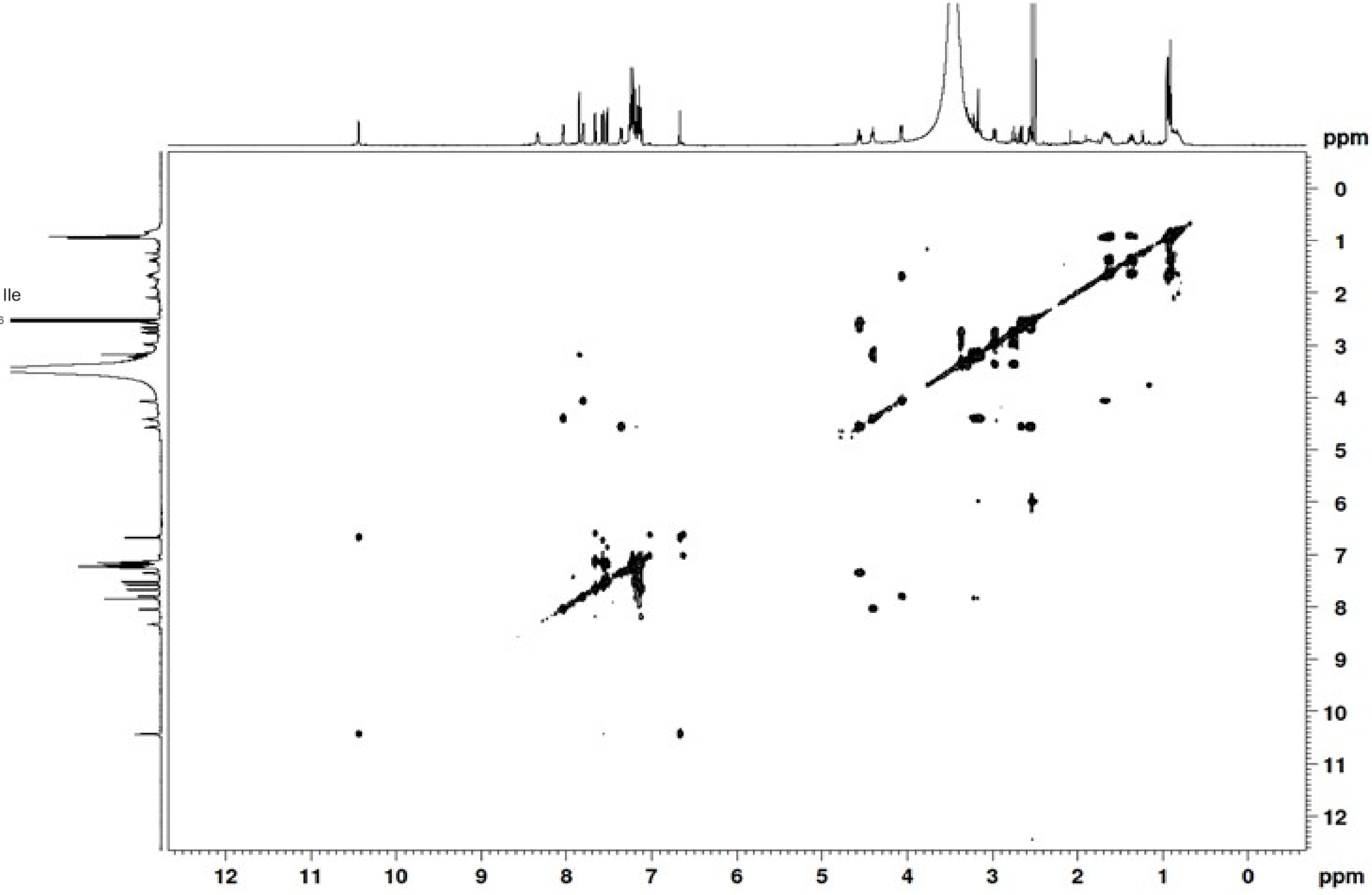

Figure S40. COSY spectrum (500 MHz) of scabrirubin (**5**) in DMSO-d_6.

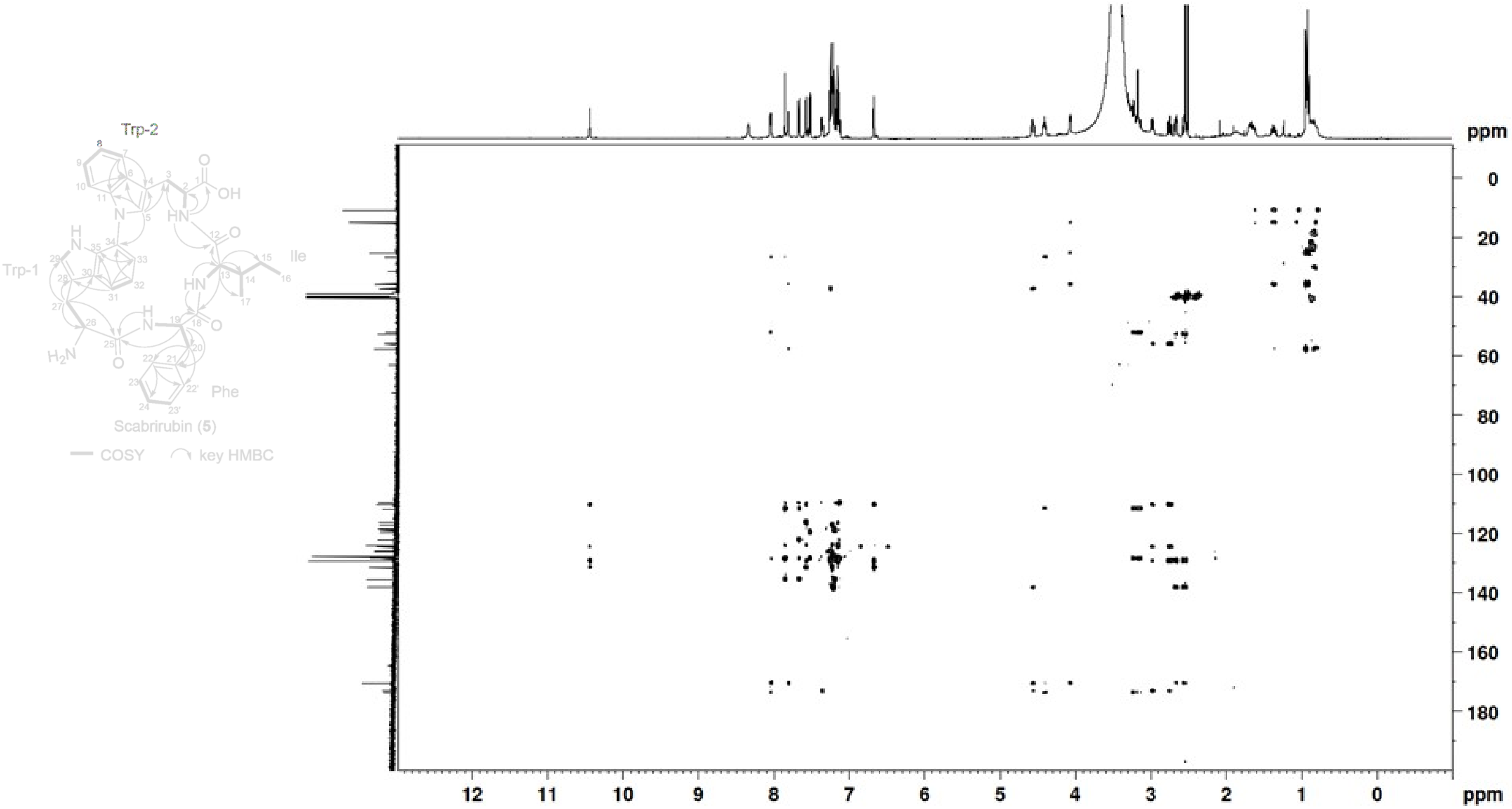

Figure S41. HMBC spectrum (500 MHz) of scabrirubin (**5**) in DMSO-d_6.

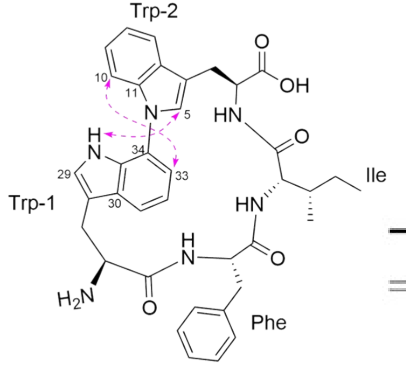

Scabrirubin (**5**)

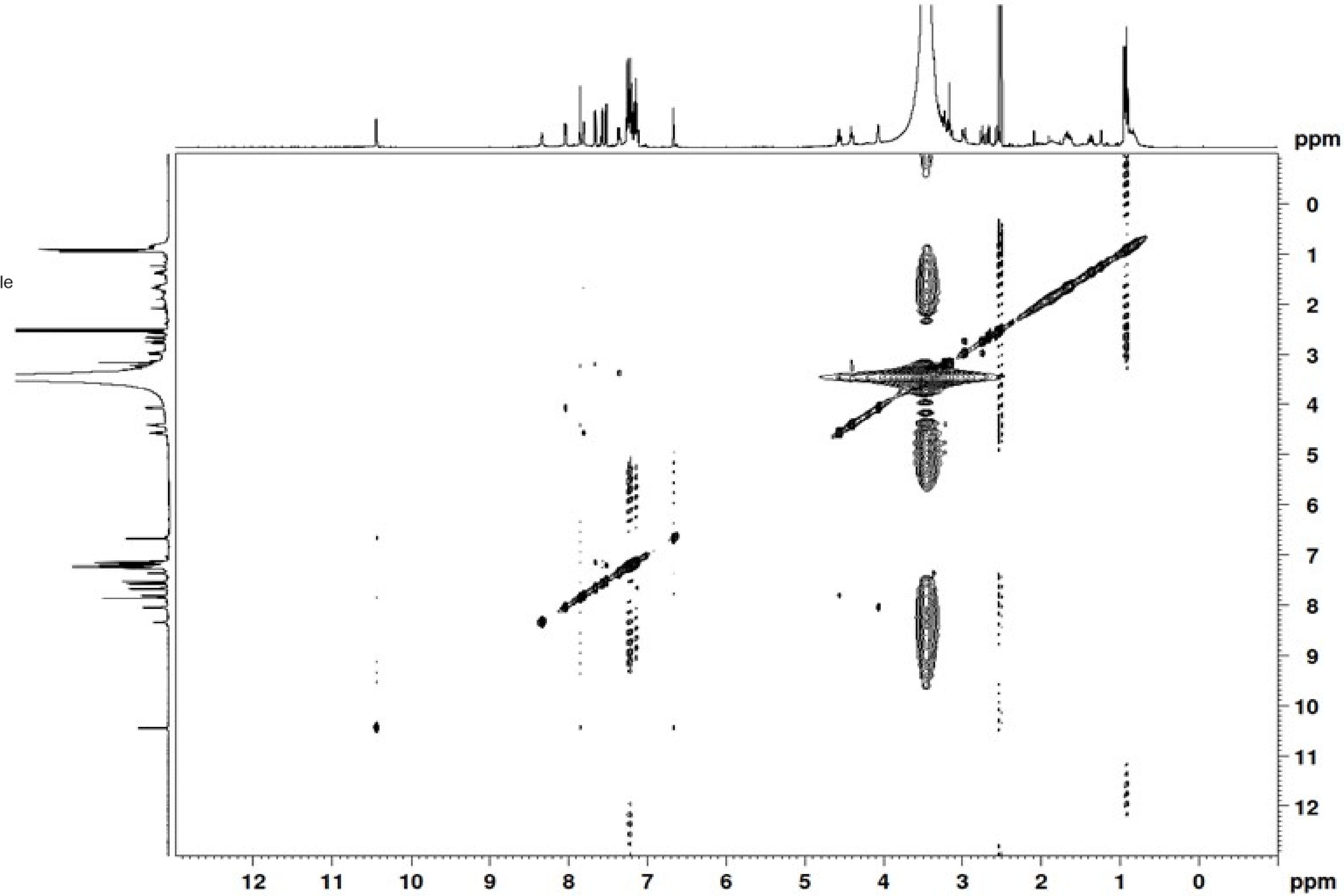

Figure S42. NOESY spectrum (500 MHz) of scabrirubin (**5**) in DMSO-d_6.

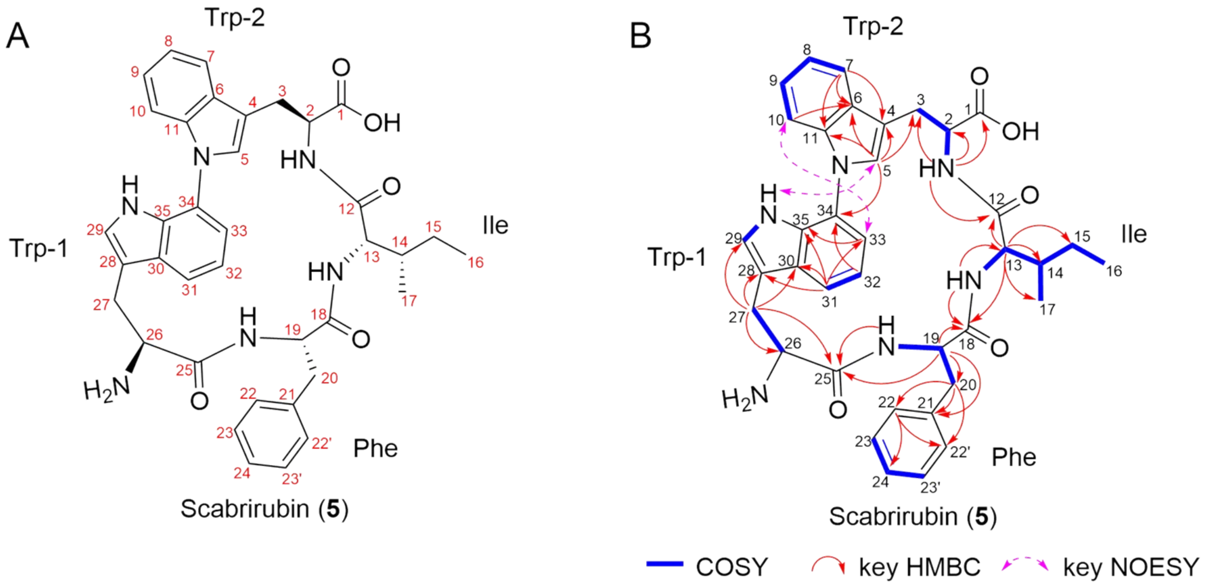

Figure S43. Structure elucidation of scabrirubin (**5**). (A) Structure of **5** and atom numbering used throughout this study. (B) Key ^{1}H-^{1}H COSY, ^{1}H-^{13}C HMBC and ^{1}H-^{1}H NOESY correlation used for determining the structure of **5**.

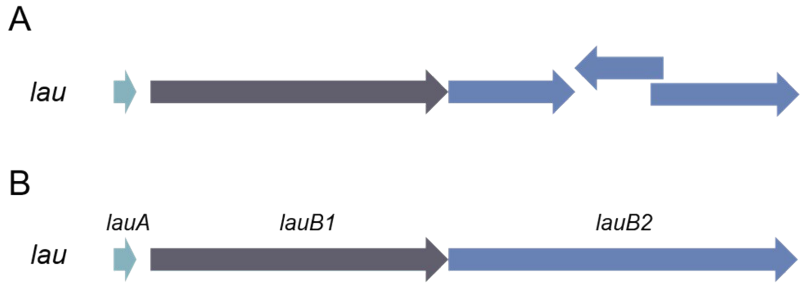

Figure S44. Resequencing of *lau* BGC. (A) the *lau* BGC downloaded from NCBI; (B) the resequenced *lau* BGC.

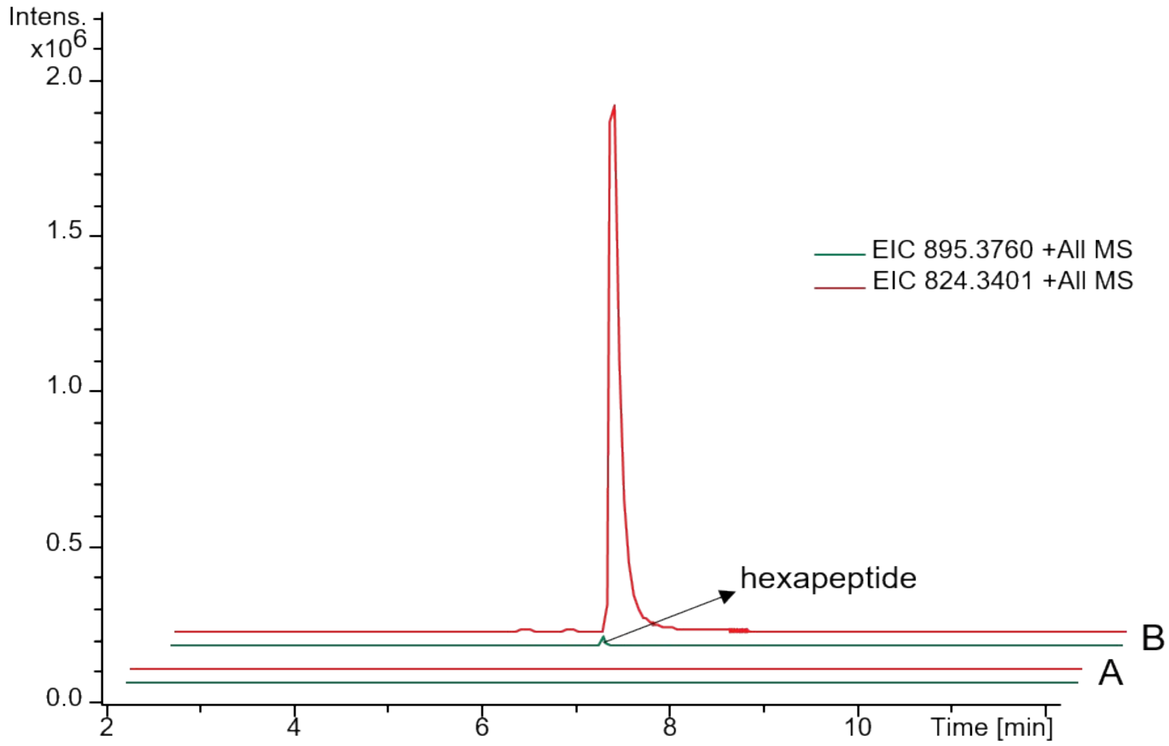

Figure S45. Extracted ion chromatogram of pentapeptide laurentirubin B (**6**) and hexapeptide from *S. albus* harboring pUWL201-oriT (A) and *S.albus* harboring pUWL201-OriT-*lau* (B), respectively.

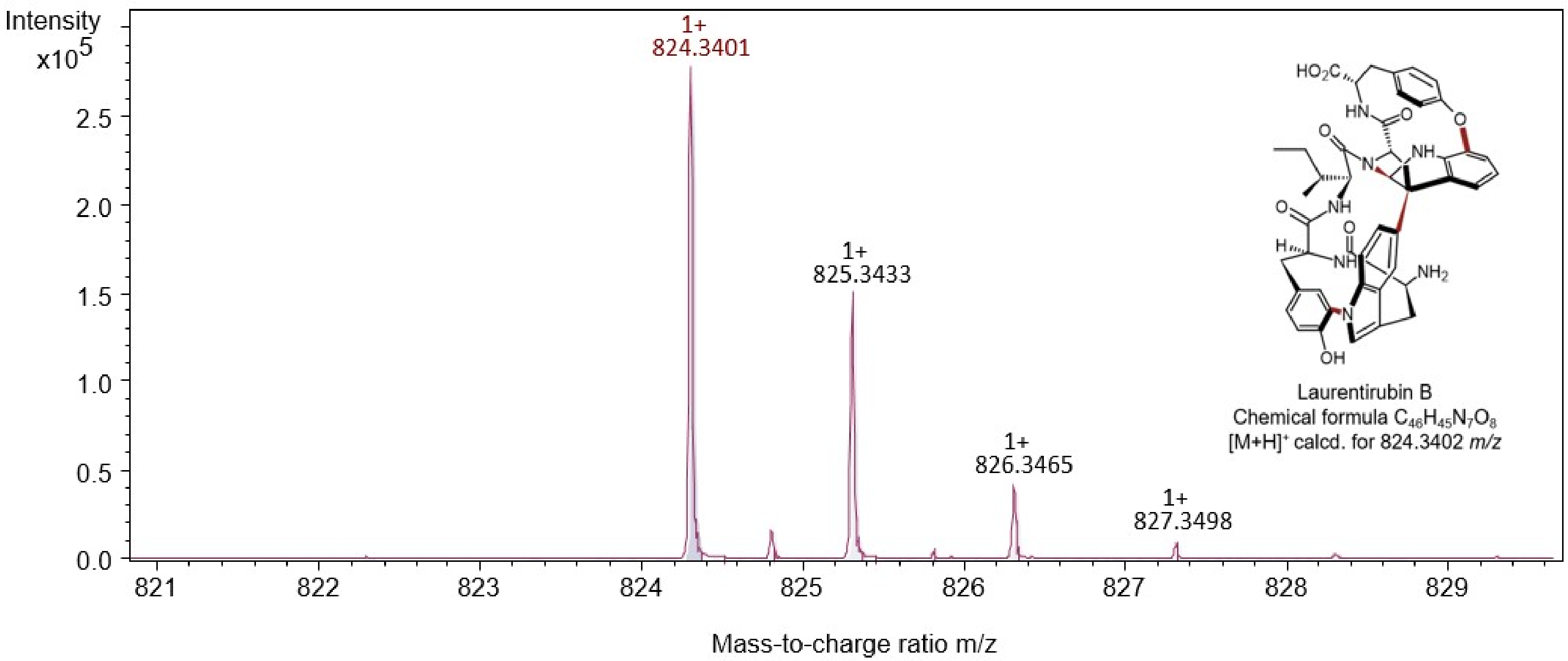

Figure S46. HPLC-ESI-QTOF-HRMS analysis of laurentirubin B (**6**).

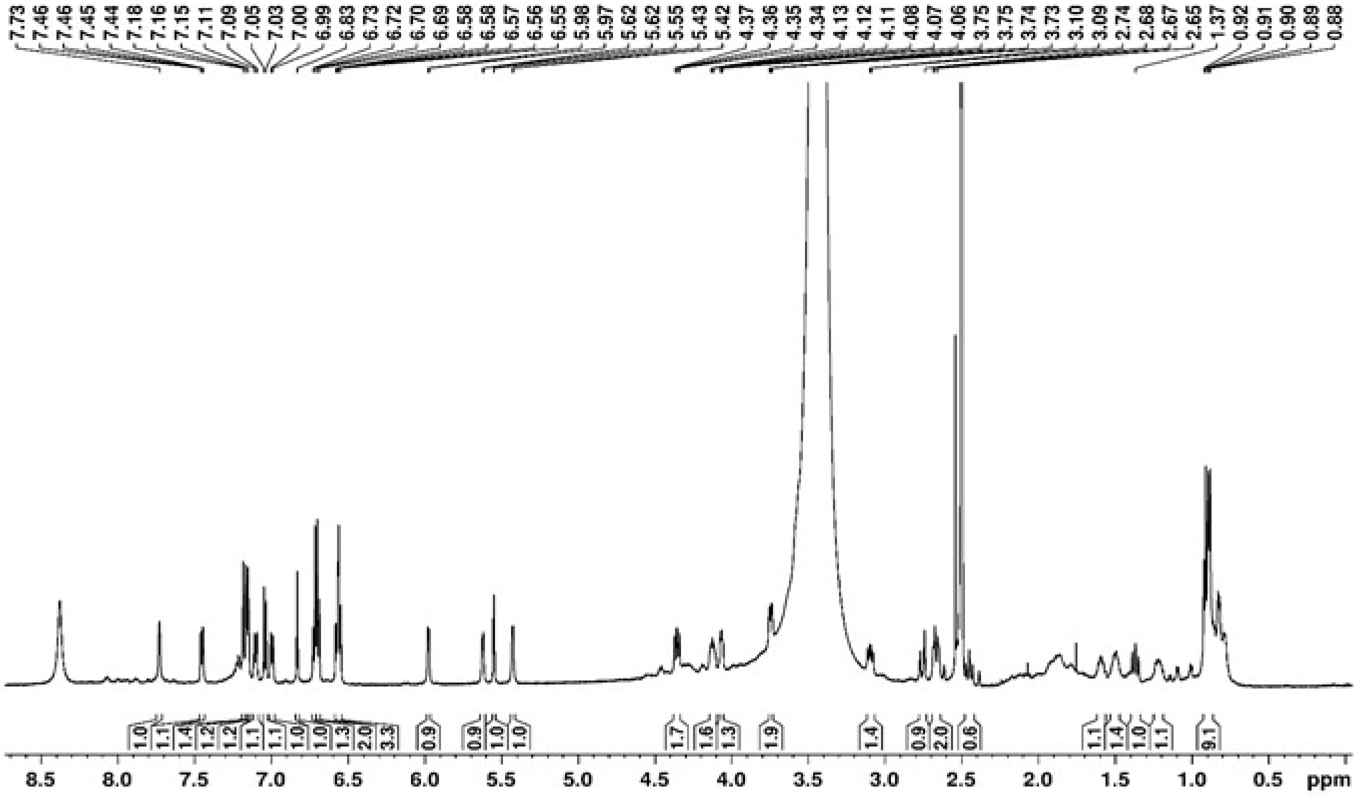

Figure S47. ^1H NMR spectrum(600 MHz) of laurentirubin B (**6**) in DMSO-d_6.

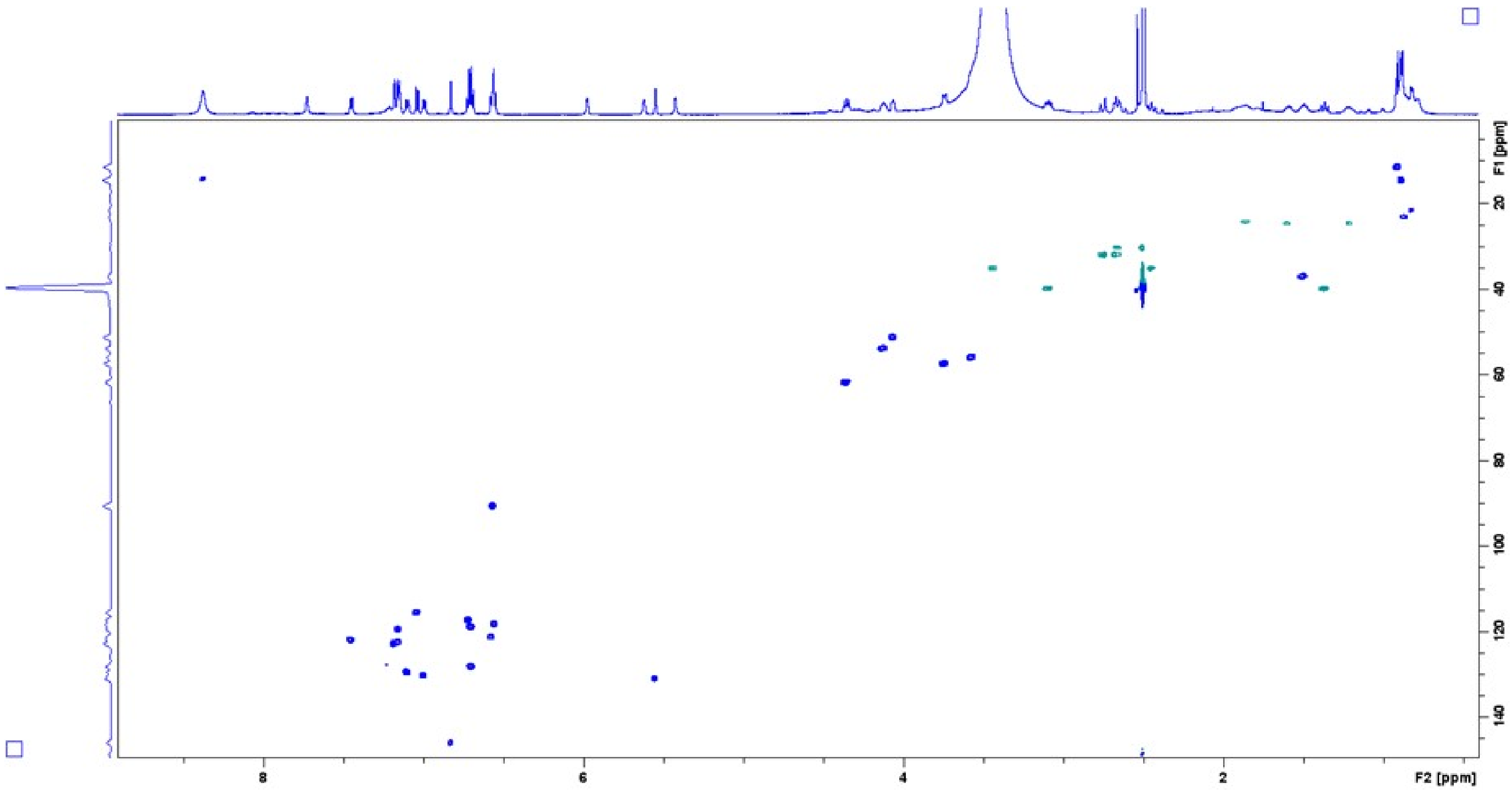

Figure S48. HSQC spectrum (600 MHz) of laurentirubin B (**6**) in DMSO-d_6.

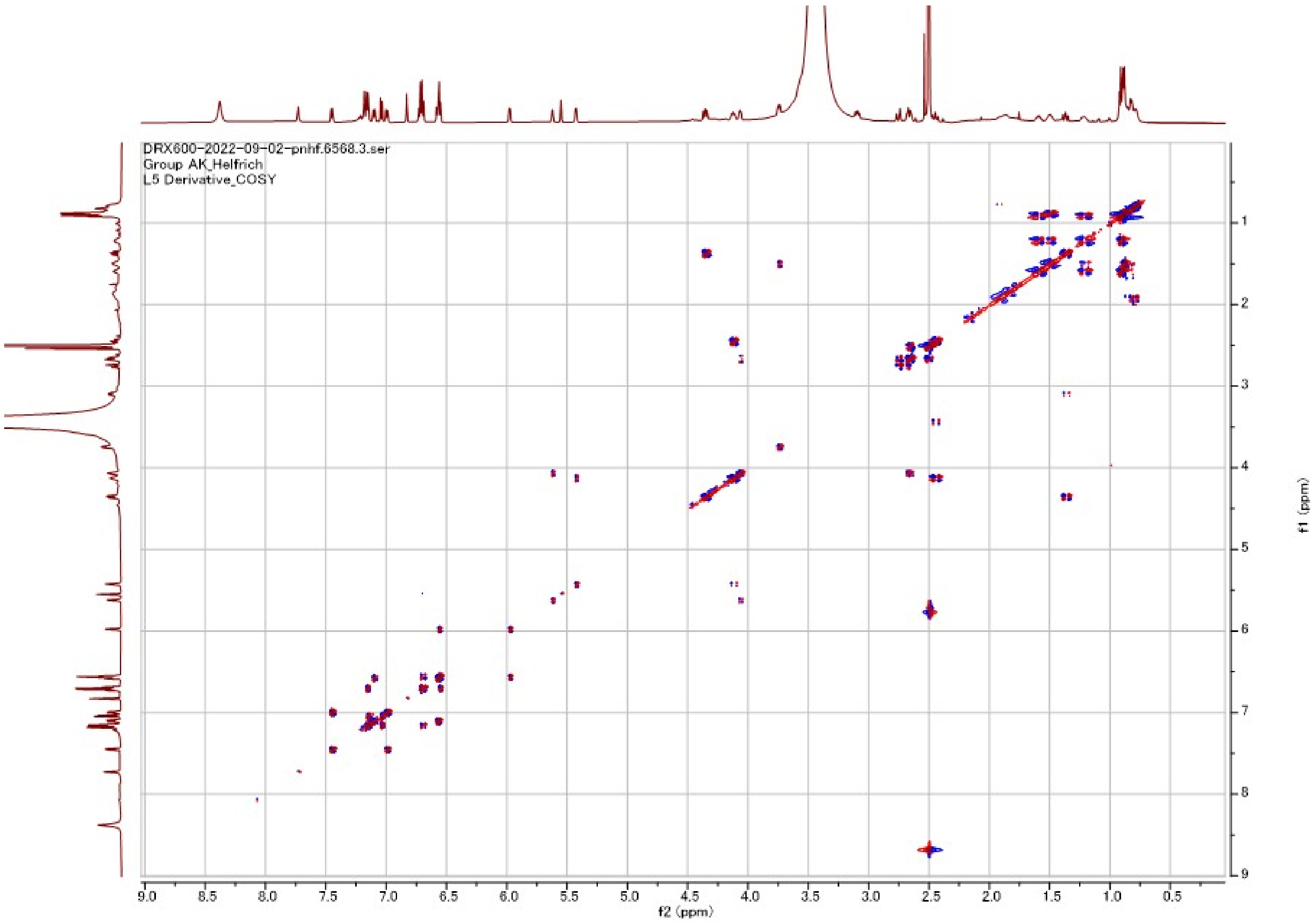

Figure S49. COSY spectrum (600 MHz) of laurentirubin B (**6**) in DMSO-d_6.

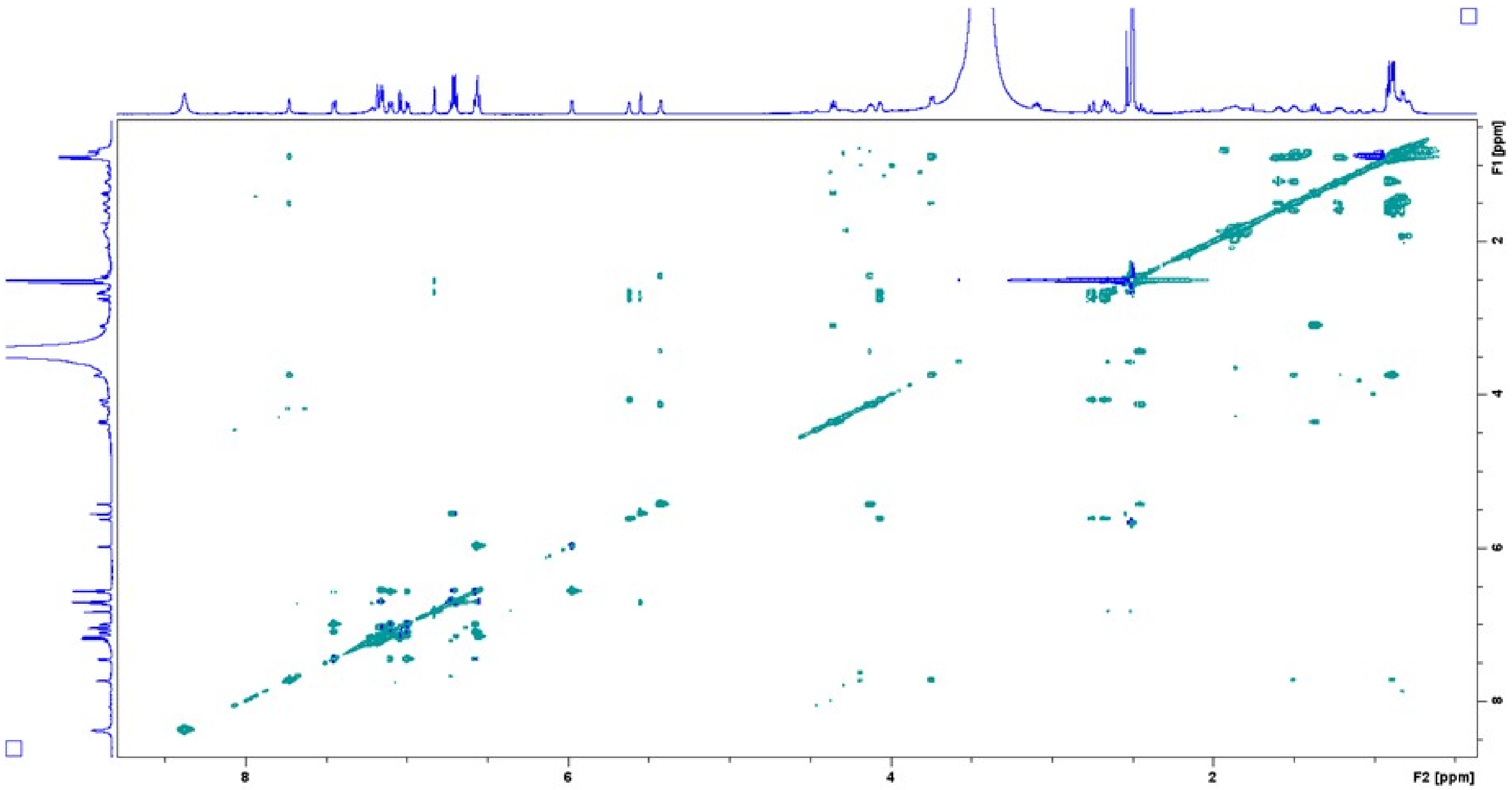

Figure S50. TOCSY spectrum (600 MHz) of laurentirubin B (**6**) in DMSO-d_6.

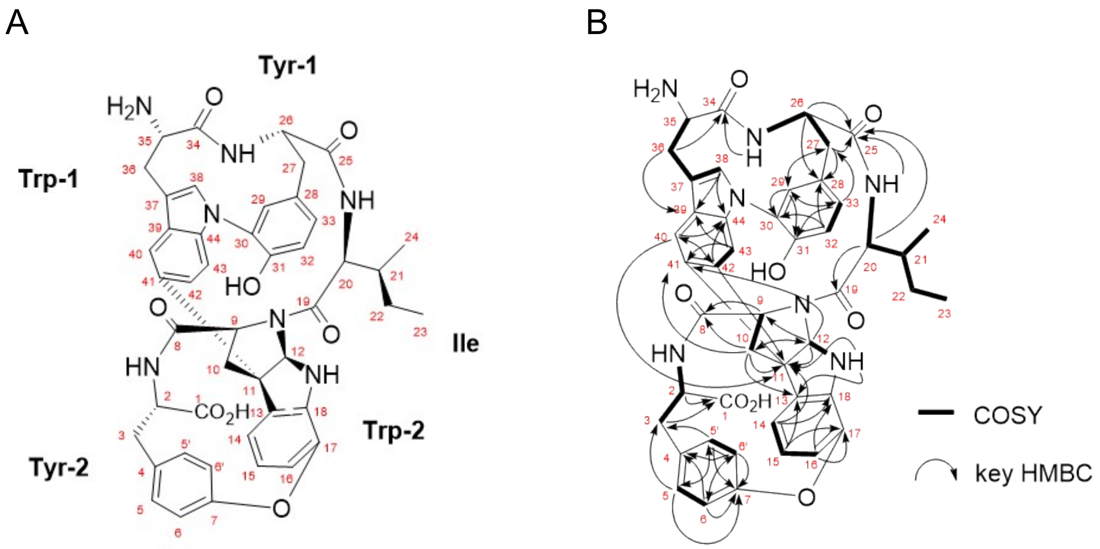

Figure S51. Structure of laurentirubin B (**6**). (A) Structure of laurentirubin B (**6**) and atom numbering used throughout this study. (B) COSY and key HMBC correlations of laurentirubin B (**6**) in DMSO-d_6.

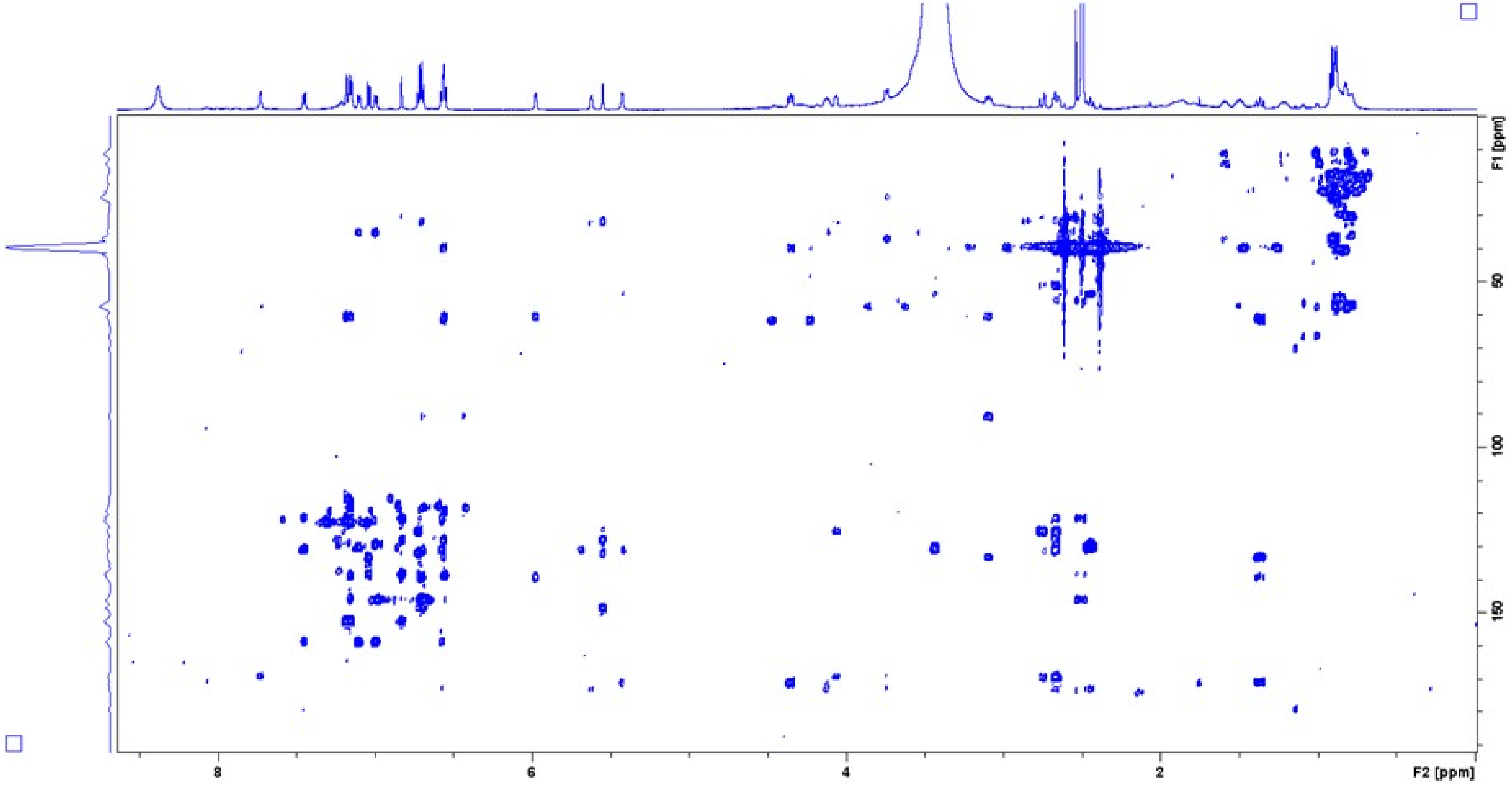

Figure S52. HMBC spectrum (J = 8 Hz) of laurentirubin B (**6**) in DMSO-d_6.

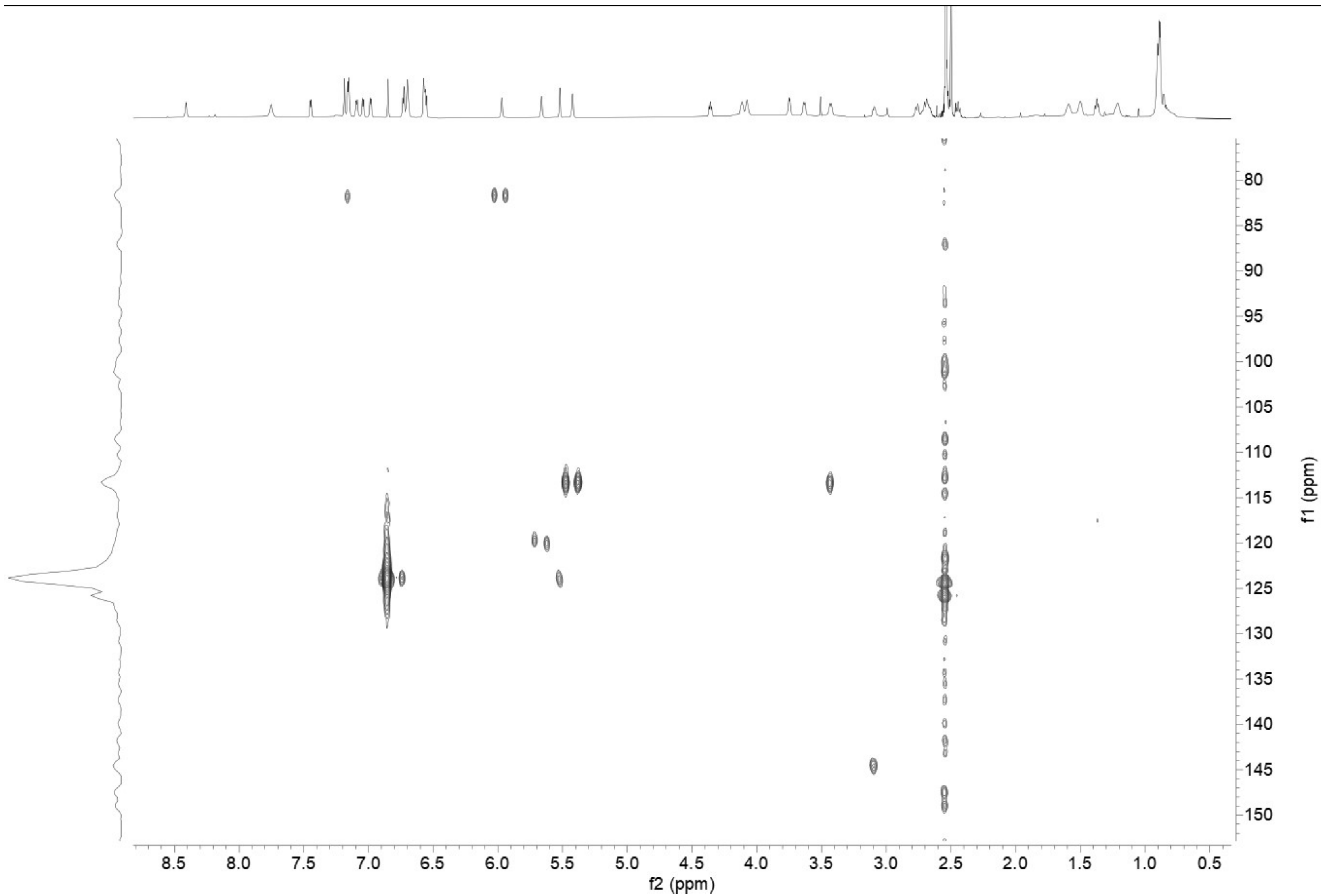

Figure S53. $^1H–^{15}N$ HMBC NMR of laurentirubin B (**6**) in DMSO-d_6.

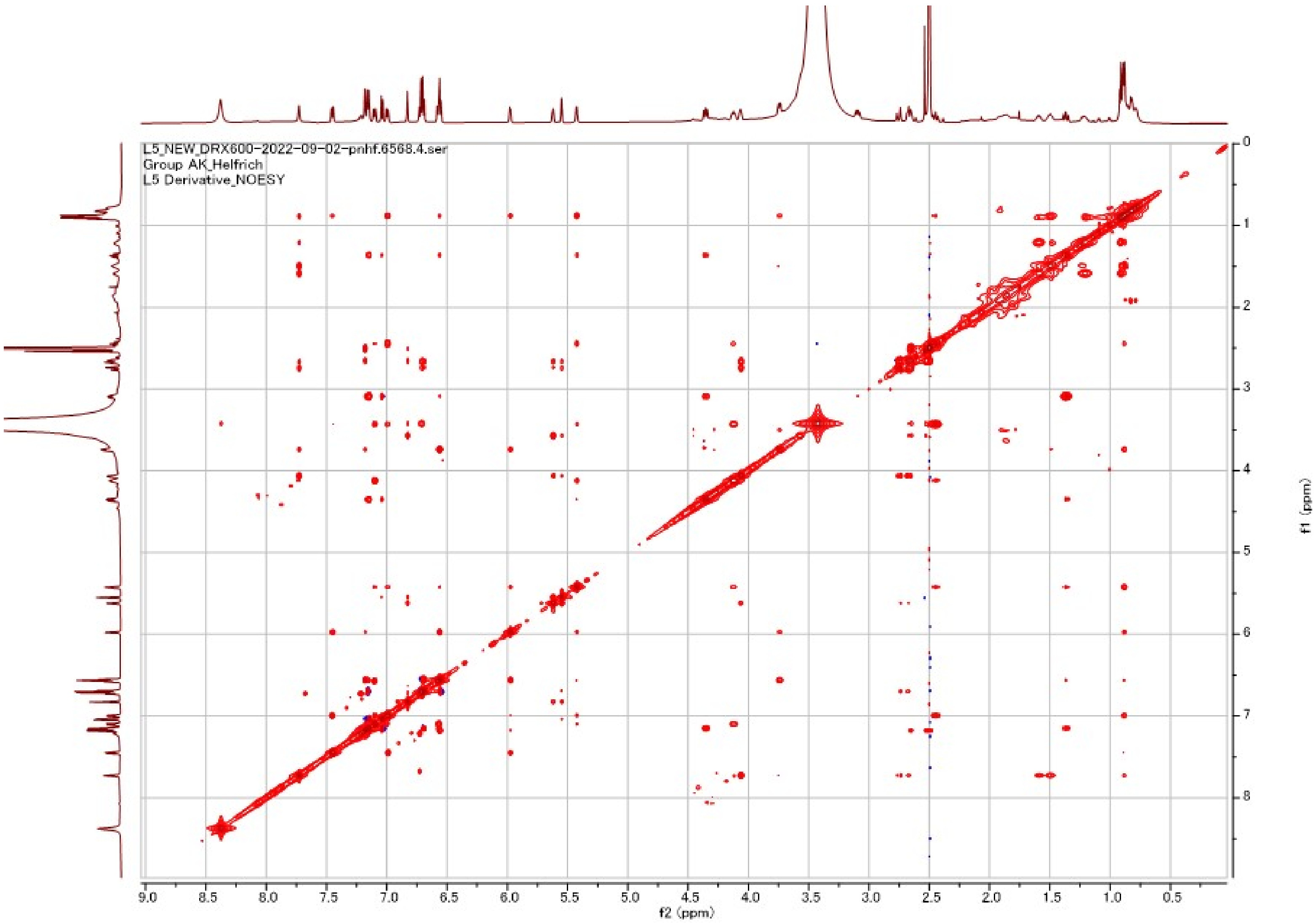

Figure S54. NOESY spectrum (600 MHz) of laurentirubin B (**6**) in DMSO-d_6.

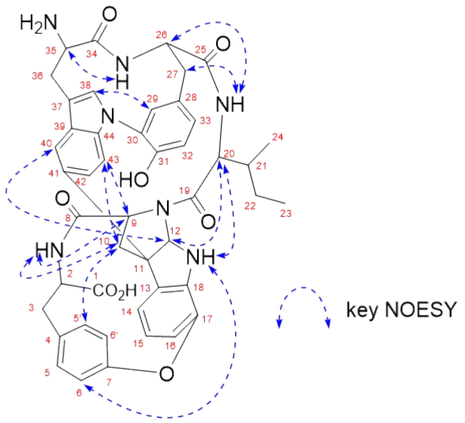

Figure S55. Key NOESY correlations of laurentirubin B (**6**).

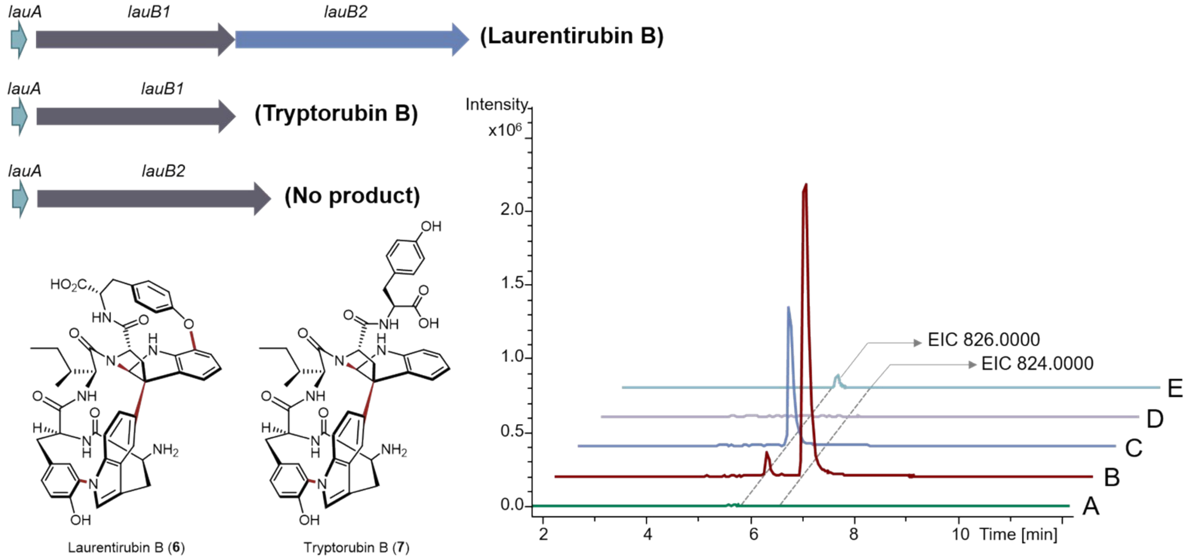

Figure S56. *In vivo* functional characterization of P450s in *lau* BGC. Extracted ion chromatogram of laurentirubin B (**6**) and tryptorubin B (**7**) together from different sources. (A) *S. albus* harboring pUWL201-oriT; (B) *S. albus*/pUWL201-OriT-*lau*; (C) *S. albus*/pUWL201-OriT-*lauA*+*lauB1*; (D) *S. albus*/pUWL201-OriT-*lauA*+*lauB2*; (E) authentic tryptorubin B standard.

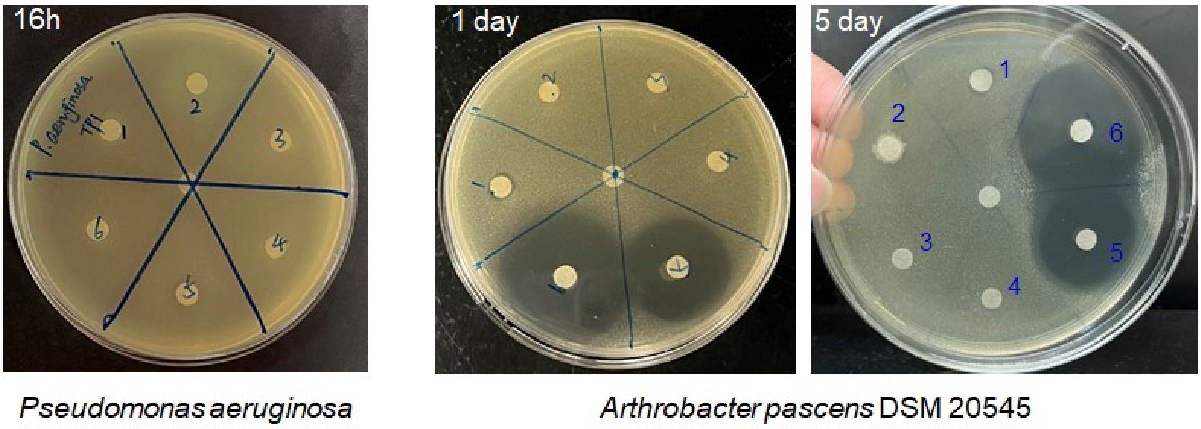

Figure S57. Antibacterial assay of compounds **2**, **3**, **4a** and **5** against *Pseudomonas aeruginosa* and *Arthrobacter pascens* DSM 20545. Labels 1, 2, 3, 4, 5, 6 represent compounds **5**, **3**, **2**, **4a**, trimethoprim and ampicillin, respectively. The central disk paper was loaded by DMSO. It shows that label 2 (compound **3**) can promote the growth of *P. aeruginosa and A. pascens DSM 20545,* and label 1 (compound **5**) displayed weak growth inhibition activity against *A. pascens* DSM 20545.

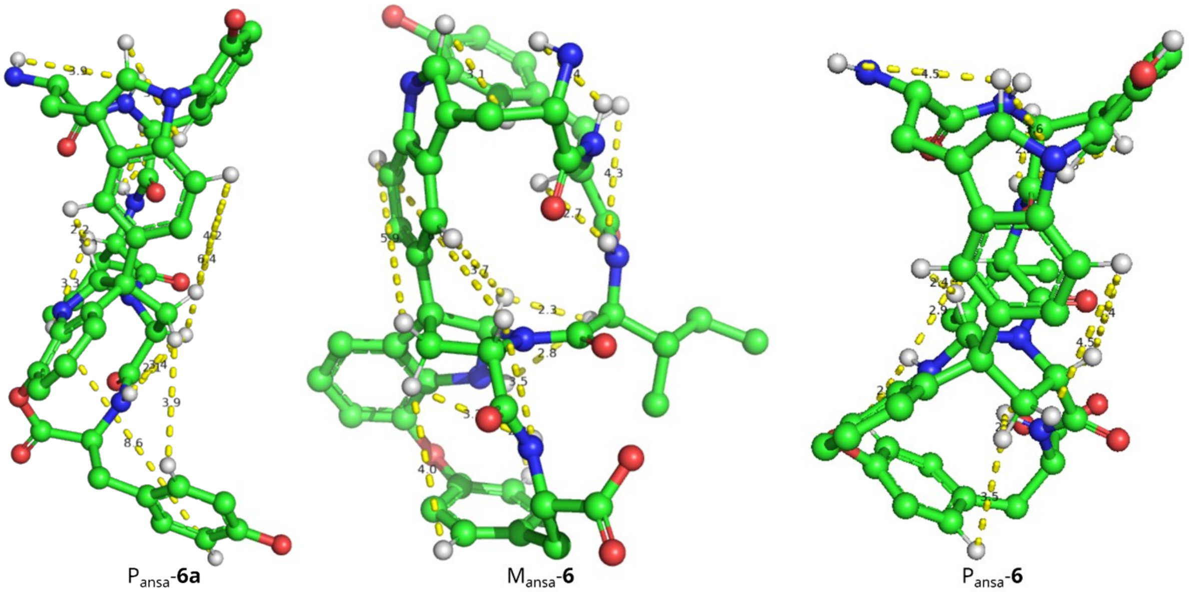

Figure S58. The lowest energy conformer of P_{ansa}-**6a**, M_{ansa}-**6**, and P_{ansa}-**6** calculated at B3LYP/6-31G(d,p) level of theory. The values show distance (Å) of protons which showed NOESY correlations in **6**. Protons not involved in one of the NOESY correlations are hidden.

Table S1. Detailed metrics of final classifiers for differentiating atropopeptide-modifying P450s (class 1) from P450s modifying any other substrate (class 0)

Classifier	Score	Balanced Accuracy Score	Cohen-Kappa Score	Matthews Correlation Coefficient	AUC Score	Cross Validation Scores	F1-Score Class 0 / 1
**RandomForestClassifier**	0.9991	0.9674	0.9659	0.9664	0.9668	[0.95182999, 0.96767131, 0.9662531, 1., 0.98823424]	[1.00, 0.97]
**ExtraTreesClassifier**	0.9992	0.9674	0.9659	0.9664	0.9848	[0.97699709, 1., 0.97699709, 1., 0.98823424]	[1.00, 0.97]
**AdaBoostClassifier**	0.9992	0.9674	0.9659	0.9664	0.9831	[0.97699709, 1., 0.97699709, 1., 0.98823424]	[1.00, 0.97]
**BaggingClassifier**	0.9978	0.9882	0.9173	0.9190	0.9877	[0.9662531, 0.96897373, 0.93667219, 0.98875096, 0.95596986]	[1.00, 0.92]
**DecisionTreeClassifier**	0.9981	0.9668	0.9238	0.9238	0.9668	[0.9662531, 1., 0.97699709, 0.97699694, 0.94611847]	[1.00, 0.92]
**MLPClassifier**	0.9970	0.9663	0.8851	0.8863	0.9927	[0.98875103, 0.97699709, 0.95182999, 0.94944904, 0.98823424]	[1.00, 0.89]

Table S2. ^{1}H and ^{13}C NMR Data for varsorubin B1 (**3**) in DMSO-d_6.

	**δ_H, mult. (*J* in Hz)**	**δ_C**		**δ_H, mult. (*J* in Hz)**	**δ_C**
1		174.1	22[a]		
2	3.87, m	52.7	22-NH_2[a]		
2-NH	7.21, d(8.0)		23		170.1
3	1.33, m	42.5	24	3.93, m	51.4
	1.39, m		24-NH	5.89, br d	
4	1.57, m	24.2	25	2.61, ovlp.[iii]	32.0
5	0.74, d (6.9)	22.7		2.68, dd (7.7, 17.4)	
6	0.76, d (6.8)	23.0	26		125.3
7		169.7	27	6.12, br s	132.4
8	4.46, dd (7.3, 12.0)	63.7	28		131.8
9	2.09, m	39.5	29		148.4
	3.29, ovlp.[i]		30	6.69, ovlp.[ii]	116.8
10		60.3	31	6.67, ovlp.[ii]	128.1
11	6.66, ovlp.[ii]	92.0	32[a]		
11-NH[a]			33	3.41, ovlp.[i]	57.1
12		135.9	33-NH_2[a]		
13	6.78, d (7.5)	122.9	34	2.54, ovlp.[iv]	30.7
14	6.64, t (7.5)	118.1		2.59, ovlp.[iii]	
15	7.09, t (7.5)	127.8	35		121.5
16	6.72, d (7.5)	109.2	36	6.72, s	145.9
17		148.7	37		138.4
18		172.7	38	7.12, s	122.7
19	4.00, m	52.6	39		135.4
19-NH	7.83, br s		40	7.34, d (8.4)	122.7
20	1.64, m	27.7	41	7.08, d (8.4)	115.4
	1.90, m		42		152.5
21	2.32, m	30.0			
	2.36, m				

a: not observed
i: overlapped with water
ii: overlapped with H-11, H-30, and H-31
iii: overlapped with H-25 and H-34
iv: overlapped with solvent

Table S3. ^{1}H and ^{13}C NMR Data for varsorubin B2a (**4a**) in DMSO-d_6.

	δ_H, mult. (J in Hz)	δ_C[a]		δ_H, mult. (J in Hz)	δ_C[a]
1		173.6	22		173.5
2	4.04, ddd (7.0, 7.0, 7.0)	51.2	22-NH_2[b]		
2-NH	7.26, d (7.0)		23		170.7
3	1.46, m	40.5	24	4.55, ddd (4.6, 9.1, 9.1)	54.0
4	1.68, ovlp.[i]	23.8	24-NH	8.01, d (9.1)	
5	0.88, d (6.5)	21.9	25	2.55, dd (9.1, 13.7)	36.3
6	0.90, d (6.7)	22.7		2.92, dd (4.6, 13.7)	
7		170.3	26		127.7
8	4.32, ddd (4.2, 8.8, 8.8)	53.4	27	6.95, d (8.3)	130.0
8-NH	6.95 br s		28	6.63, d (8.3)	114.6
9	3.41, dd (4.2, 15.2)	25.7	29		155.5
	3.48, dd (8.8, 15.2)		30	6.63, d (8.3)	114.6
10		106.4	31	6.95, d (8.3)	130.0
11		136.9	32		174.7
11-NH[a]			33	3.30, ovlp.[ii]	57.8
12		128.8	33-NH_2[a]		
13	7.51, d (7.9)	117.8	34	2.86, (dd, 3.0, 13.4)	30.9
14	6.98, t (7.9)	118.1		3.03, (dd, 11.1, 13.4)	
15	7.06, t (7.9)	120.5	35		110.6
16	7.31, d (7.9)	110.5	36	7.24, d (1.8)	124.9
17		135.4	36-NH	11.0, br s	
18		169.4	37		135.9
19	3.68, ddd (6.8, 6.8, 6.8)	52.1	38	7.43, s	111
19-NH	5.58, br s		39[a]		
20	1.31, m	26.5	40	7.22, d (8.3)	118.3
	1.59, m		41	7.48, d (8.3)	119.1
21	1.70, ovlp.[i]	30.6	42		125.9
	1.83, ddd (5.9, 8.9, 14.8)				

a: assigned by cross peaks in the HSQC and HMBC spectra
b: not observed
i: overlapped with H-4 and H-21
ii: overlapped with water

Table S4. ^1H and ^{13}C NMR data of scabrirubin (**5**) in DMSO-d_6

position	δ_H, mult. (J in Hz)	δ_C, type
1		173.7, C
2	4.40, m	52.3, CH
2-NH	8.04, d (5.7)	
3	3.24, dd (16.0, 3.3)	26.8, CH_2
	3.15, m	
4		111.7, C
5	7.85, s	128.6, CH
6		128.4, C
7	7.66, d (7.9)	118.9, CH
8	7.12, m	119.6, CH
9	7.19, m	122.1, CH
10	7.52, d (8.0)	109.6, CH
11		135.5, C
12		170.5, C
13	4.06, dd (7.1, 3.8)	57.8, CH
13-NH	7.81, d (3.8)	
14	1.68, m	35.8, CH
15	1.66, m	25.3, CH_2
	1.36, m	
16	0.91, t (7.4)	10.9, CH_3
17	0.94, d (6.8)	15.1, CH_3
18		170.5, C
19	4.56, ddd (10.4, 9.3, 3.7)	52.8, CH
19-NH	7.35, d (9.3)	
20	2.67, dd (14.1, 3.7)	37.4, CH_2
	2.57, dd (14.1, 10.4)	
21		138.1, C
22	7.25, m	129.2, CH
23	7.21, m	127.9, CH
24	7.14, m	126.1, CH
23′	7.21, m	127.9, CH
22′	7.25, m	129.2, CH
25		173.7, C
26	3.35, m	56.1, CH
27	2.98, dd (13.2, 3.8)	31.6, CH_2
	2.75, dd (12.5, 12.5)	
28		110.3, C
29	6.66, d (2.2)	124.4, CH
29-NH	10.45, s	
30		129.3, C
31	7.57, d (7.85)	117.1, CH
32	7.15, m	118.3, CH
33	7.23, m	116.2, CH
34		123.9, C
35		131.6, C

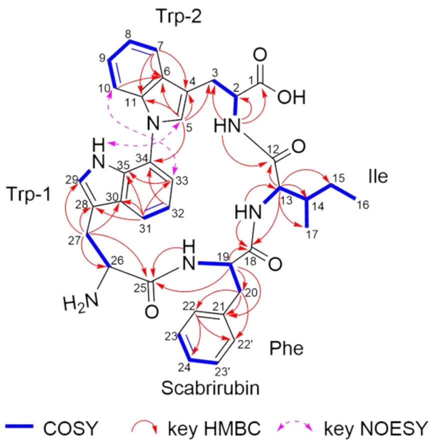

Table S5. ^{1}H and ^{13}C NMR data of laurentirubin B (**6**) in DMSO-d_6

	δ_H, mult.	δ_C[a]		δ_H, mult.	δ_C[a]
1		173.0	23	0.91, t (7.3)	11.3
2	4.12, m	53.8	24	0.89, d (6.6)	14.4
2-NH	5.43, d (3.9)		25		169.3
3	2.45, t (13.1)	35.1	26	4.07, m	51.1
	3.44, ovlp.[i]		26-NH	5.62, d (3.6)	
4		130.9	27	2.66, ovlp.[v]	31.9
5	7.00, br d	130.2		2.76, d (17.3)	
5'	7.10, br d	129.3	28		125.4
6	7.45, dd (2.4, 8.2)	121.9	29	5.55, s	131.0
6'	6.58, ovlp.[ii]	121.2	30		131.9
7		158.9	31		148.5
8		171.2	31-OH		
9	4.36, dd (7.8, 10.9)	61.7	32	6.72, ovlp.[iii]	117.3
10	1.37, br dd	39.8	33	6.70, ovlp.[iii]	128.2
	3.09, dd (7.8, 13.5)		34		173.2
11		60.5	35	3.58, m	55.8
12	6.57, ovlp.[ii]	90.6	35-NH_2		
12-NH	5.98, d (4.5)		36	2.51, ovlp.[vi]	30.2
13		139.2		2.66, ovlp.[v]	
14	6.55, ovlp.[ii]	118.2	37		121.5
15	6.70, ovlp.[iii]	118.9	38	6.83, s	145.9
16	7.16, ovlp.[iv]	119.4	39		138.4
17		145.7	40	7.18, s	122.7
18		138.7	41		133.3
19		172.8	42	7.16, ovlp.[iv]	122.4
20	3.75, dd (2.8, 9.3)	57.3	43	7.04, d (8.3)	115.4
20-NH	7.73, br s		44		152.5
21	1.50, m	36.9			
22	1.22, m	24.6			
	1.59, m				

a: assigned by cross peaks in the HSQC and HMBC spectra
i: overlapped with water signal
ii: overlapped with H-6', H-12, and H-14
iii: overlapped with H-15, H-32, and H-33
iv: overlapped with H-16 and H-42
v: overlapped with H-27 and H-36
vi: overlapped with solvent

Table S6. Comparison of experimental and calculated ^{13}C NMR chemical shifts for **6**

no.	exp. δ_C	P_{ansa}-**6**	P_{ansa}-**6a**[a]	M_{ansa}-**6**
1	173.0	175.8	167.6	169.3
2	53.8	49.6	67.7	55.5
3	35.1	35.2	45.4	37.1
4	130.9	132.3	129.3	133.0
5	130.2	133.8	130.4	130.8
6	129.3	128.9	129.4	130.5
7	121.9	120.5	115.6	122.0
8	121.2	120.5	113.7	120.5
9	158.9	163.4	154.7	161.7
10	171.2	168.4	168.9	168.2
11	61.7	67.0	64.6	67.9
12	39.8	39.8	49.9	45.2
13	60.5	67.4	76.6	68.7
14	90.6	95.9	91.3	91.8
15	139.2	140.4	138.3	139.4
16	118.2	119.4	120.7	119.6
17	118.9	119.1	117.9	119.7
18	119.4	121.9	121.5	122.5
19	145.7	148.1	135.8	147.5
20	138.7	140.4	146.0	139.3
21	172.8	169.0	166.7	174.2
22	57.3	60.7	64.6	62.2
23	36.9	42.6	49.2	40.5
24	24.6	29.6	33.0	26.8
25	11.3	12.0	19.2	11.7
26	14.4	11.1	19.3	12.4
27	169.3	174.1	174.0	169.8
28	51.1	55.9	61.6	65.5
29	31.9	34.1	40.8	29.4
30	125.4	126.0	127.0	129.7
31	131.0	136.1	135.2	125.5
32	131.9	135.4	136.1	130.3
33	148.5	149.2	148.6	150.2
34	117.3	114.2	115.5	116.7
35	128.2	128.0	128.5	128.5
36	173.2	173.9	175.7	180.5
37	55.8	64.1	68.1	58.6
38	30.2	35.6	36.9	32.3
39	121.5	122.1	124.9	130.2
40	145.9	145.7	146.5	144.9
41	138.4	140.2	140.1	138.7
42	122.7	122.8	122.1	124.0
43	133.3	136.8	140.4	140.0
44	122.4	123.3	123.9	127.6
45	115.4	116.0	117.4	116.8
46	152.5	153.1	151.6	160.8
MAE[b]		2.57	4.96	3.00
MSE[c]		10.97	41.57	17.38

[a]Compound **6a** has aryl ester bridge between C-1 and C-17 instead of aryl ether bond of C-7 and C-17. [b]Mean absolute errors (ppm). [c]Mean squared error.

Table S7. Strains and plasmids used and constructed in this study

Strains/plasmids	Characteristic(s)	Sources
E. coli		
DH5a	Host strain for cloning	[9]
ET12567/pUZ8002	Donor strain for conjugation, Chl^r, Kan^r	[10]
**Actinobacteria**		
Streptomyces jumonjinensis DSM 747	Wild type strain containing *jum* BGC	DSMZ[a]
Streptomyces varsoviensis DSM 40346	Wildtype strain containing *sva* BGC	DSMZ[a]
Embleya scabrispora DSM 41855	Wild type strain containing *sca* BGC	DSMZ[a]
Streptomyces laurentii DSM 41684	Wildtype strain containing *lau* BGC	DSMZ[a]
S. albus J1074	Heterologous host	[11]
S. albus J1074/pUWL201-OriT	The introduction of pUWL201-OriT into *S. albus* J1074	This study
S. albus J1074/pUWL201-OriT-*jum*	The introduction of pUWL201-OriT-*jum* into *S. albus* J1074	This study
S. albus J1074/pUWL201-OriT-Sva	The introduction of pUWL201-OriT-Sva into *S. albus* J1074	This study
S. albus J1074/pUWL201-OriT-SvaR	The introduction of pUWL201-OriT-SvaR into *S. albus* J1074	This study
S. albus J1074/pUWL201-OriT-*sca*	The introduction of pUWL201-OriT-*sca* into *S. albus* J1074	This study
S. albus J1074/ pUWL201-OriT-*lau*	The introduction of pUWL201-OriT-*lau* into *S. albus* J1074	This study
S.albus J1074/ pUWL201-OriT-*lauA*+*lauB1*	The introduction of pUWL201-OriT-*lauA*+*lauB1* into *S.albus* J1074	This study
S.albus J1074/ pUWL201-OriT-*lauA*+*lauB2*	The introduction of pUWL201-OriT-*lauA*+*lauB2* into *S.albus* J1074	This study
**Plasmids**		
pUWL201-OriT	Apr^r, *ermE**p, replicative expression vector in *Streptomyces*	[12]
pUWL201-OriT-deletion	Deletion of space between RBS and MCS in pUWL201-OriT	This study
pUWL201-OriT-*jum*	Apr^r, *jum* BGC was constructed into pUWL201-OriT	This study
pUWL201-OriT-Svarcore1	Apr^r, *sva* BGC containing *svaA2* and *svaB* was constructed into pUWL201-OriT	This study
pUWL201-OriT-Svarcore2	Apr^r, *sva* BGC containing *svaA1, svaA2* and *svaB* was constructed into pUWL201-OriT	This study
pUWL201-OriT-Svarcore1R	Apr^r, a RBS was inserted in front of *svaB* based on plasmid pUWL201-OriT-Svarcore1	This study
pUWL201-OriT-Svarcore2R	Apr^r, a RBS was inserted in front of *svaB* based on plasmid pUWL201-OriT-Svarcore2	This study
pUWL201-OriT-*sca*	Apr^r, *sca* BGC was constructed into pUWL201-OriT	This study

pIJ10257	Hyg[r], *ermE**p, integrative expression vector in *Streptomyces*	[13]
pIJ10257-*lau*	Hyg[r], *lau* BGC was constructed into pIJ10257	This study
pUWL201-OriT-*lau*	Apr[r], *lau* BGC was constructed into pUWL201-OriT	This study
pUWL201-OriT-*lauA*+*lauB1*	Apr[r], *lauA and lauB1* was constructed into pUWL201-OriT	This study
pUWL201-OriT-*lauA*+*lauB2*	Apr[r], *lauA and lauB1* was constructed into pUWL201-OriT	This study

DSMZ[a]: Deutsche Sammlung von Mikroorganismen und Zellkulturen.

Table S8. Primers used in this study

Primers	Sequences (5' to 3')
**For amplifying *jum* BGC**	
jumo_fwd	gaggcttgatATGAAGGTTCTCTTTGCCATTCGGCACAAGGTCAC
jumo_rev	ggaattcgatTCAGCGGACCCGGGCCGC
**For deletion of bases in pUWL-201-OriT**	
pUWL_del_fwd	CTT GAT ATC GAA TTC CTG C
pUWL_del_rev	CCT CCT GTT CTA GAC GAT C
**For linearizing pUWL201-OriT-deletion to insert *jum* BGC**	
jumo_bb_fwd	ggtccgctgaATCGAATTCCTGCAGCCC
jumo_bb_rev	gaaccttcatATCAAGCCTCCTGTTCTAG
**For amplyifing *sva* BGC**	
Sva_fwd	TCTAGAACAGGAGGCCCCATATGGAGGAATTTATGAAGCTGGTTCAC CTG
Sva_2_rev	TCGATATCAAGCTTATCGATTCACGGGCGCACCATCCG
**For linearizing pUWL201-oriT to insert *sva* BGC**	
pUWL_OriT_fwd	ATCGATAAGCTTGATATCGAATTCCTGCAGC
pUWL_RBS_rev	ATGGGGCCTCCTGTTCTAGACGATC
**For inserting RBS into pUWL201-OriT (*sva* cloning)**	
Svar_RBS_CYP450_Fwd	AGGAGGTCACCCATGCCAATGCATCGC
Svar_CYP450_Rev	ACTCCGTTGCTGGGCTGCCC
**For amplifying *sca* BGC**	
sca-pp-F	GTCTAGAACAGGAGGCCCCATGTGATCAAGATCGTCAACTC
sca-pp-R	CAGGAATTCGATATCAAGCTTTCACCTCCCCGAGGCGAGG
**For linearizing the pUWL201-oriT to insert *sca* BGC**	
pUWL201-OriT-F	aagcttgatatcgaattcc
pUWL201-OriT-R	atggggcctcctgttctag
**For amplifying *lau* BGC**	
TrypLaurentii_Fwd_NdeI	CGCCATATGATGAAGCTTCTCTTCGCCATTCGC
TrypLaurentii_RV_XhoI	CCGCTCGAGTCAGTGTAGGCGGACCGGGAGG
**For reversely amplifying plasmid containing *lauA* and *lauB1***	
lauAB1-F	ATCGATaagcttgatatcgaattcctgcag

lauAB1-R	TCAGACAGTGGCGGCACGCC
**For amplifying lauA fragment**	
lauA-F	gtctagaacaggaggccccatATGATGAAGCTTCTCTTCGC
lauA-R	GACAGTGGCGGCACGGCAAGTCTCCGGGAGGCCGAG
**For amplifying lauB2 fragment**	
lauB2-F	GCCTCCCGGAGACTTGCCGTGCCGCCACTGTCTGAAC
lauB2-R	ttcgatatcaagcttATCGATTCAGTGTAGGCGGACCGGGAG
**For linearizing pUWL201-OriT to coexpress *lauA* and *lauB2***	
pUWL-LF	ATCGATaagcttgatatcg
pUWL-LR	atggggcctcctgttctag
